# Colorado Burden of Disease, Injuries and Risk Factors, 1990–2019: A Sub-Analysis of the Global Burden of Disease Study

**DOI:** 10.3390/ijerph19010288

**Published:** 2021-12-28

**Authors:** Jen Roux, David Rojas-Rueda

**Affiliations:** 1 Colorado School of Public Health, Colorado State University, 1601 Campus Delivery, Fort Collins, CO 80523, USA; Jen.Roux@colostate.edu; 2 Department of Environmental and Radiological Health Sciences, Colorado State University, Fort Collins, CO 80523, USA

**Keywords:** Colorado, burden of disease, injuries, risk factors, DALY

## Abstract

(1) Background: Health disparities across the United States (U.S.) are increasing. Large variations in risk factors and health outcomes have been described among states from the U.S. (2) AIM. This study aims to describe health trends in morbidity, mortality, and risk factors from 1990 to 2019 in the State of Colorado. (3) Methods: We describe the measures of health loss for 286 causes of death, 369 diseases and injuries, and 87 risk factors for the state of Colorado from the Global Burden of Disease project estimates between 1990 to 2019. (4) Results: We found that 21,171 and 40,724 deaths were estimated in 1990 and 2019, respectively, in Colorado. The leading cause of death, in both sexes, in 1990 and 2019 was ischemic heart disease (IHD). The top leading disability-adjusted life years (DALY) diagnoses were IHD, followed by low back pain, chronic obstructive pulmonary disease, and opioid use disorder. In 2019, the top risk factors by DALYs in Colorado were smoking, drug use, high body mass index (BMI), alcohol use, high fasting plasma glucose, and high systolic blood pressure. (5) Conclusion: Non-communicable diseases and their related risk factors are the top leading causes of DALYs in Colorado. Findings support the need for policies to prevent non-communicable diseases, with special attention to musculoskeletal disorders and interventions to reduce tobacco, alcohol, and drug use.

## 1. Introduction

Health disparities in morbidity, mortality, and other measures of well-being across the United States (U.S.) are increasing [1]. Large variation in risk factors and, consequently, health outcomes have been described among states in the U.S. [2]. In 2016, the U.S. burden of disease assessment highlighted the need for state-specific reporting to fully understand the significant differences in health outcomes and risk factors [3]. Measuring disease burden across geographic areas provides a comprehensive epidemiological assessment that can inform health policy decision making, resource allocation, and prioritization [2].

The Global Burden of Disease (GBD) project provides an annual assessment of the health of populations, broken down by age, sex, cause-specific mortality and morbidity, risk factor exposure, and mortality and morbidity attributable to those risks [3]. The GBD project provides descriptive and analytical tools to align health systems to the populations they serve [4]. Understanding the difference in national and state health outcomes and burden enables more effective prevention and social policy by catering to specific population needs. Moreover, while state to national health trends might be similar, closer geographic-specific analysis of those differences allows a better grasp on the nature of change [3].

Colorado is a state in the Western United States located in the Rocky Mountain region with a population of 5,758,736 as of 2019 [5,6]. Comparatively, Colorado had 3,294,394 inhabitants in 1990. In 2019, 22% of the population was under 18 years old, 14% was 65 years and older, and 49% was female [5]. From 2017 to 2018, Colorado saw a 1.4% increase in population, which ranked it as the seventh highest growth rate in the country [6]. In 2019, 87% of the population was white, and 22% Hispanic or Latino [5]. In 2019, 92% of the population had a high school degree or higher, and 41% had a bachelor’s degree or higher [5]. The median income household was USD 72,331 per year, and 9% of the population lived in poverty (vs. 13% in the whole U.S.) [5,6]. In 2019, 9% of the population under age 65 did not have health insurance [5]. In 2010, the population per square mile was 48.5 (vs. 87.4 in the whole U.S.), and the land area in square miles was 103,6471 [5]. The top industries based on employment in the state of Colorado are health services, professional and technological services, retail trade and accommodation and food services [6]. Colorado has a decentralized health system structure where its 64 counties are required to operate a local public health agency or participate in a district public health agency.

Between 1990 and 2016, the U.S. has reported at national increased impacts on disability-adjusted life years (DALY) from chronic obstructive pulmonary diseases (COPD) and opioid use disorders (OUD), and reductions in lung cancer, motor vehicle road injuries, and ischemic stroke [3]. At the global scale, despite population growth and shifts in age structure, health continues to improve [6]. However, the absolute burden of disease and its impact on health systems remains constant [6]. Health trend monitoring and policy evaluation can be utilized to counteract the effects of disease burden [6]. There are no previous studies on the burden of disease, injuries, and risk factors for Colorado. This study aims to report state-level data for the State of Colorado using the GBD 2019 estimates and describe health trends in morbidity, mortality, and risk factors from 1990 to 2019.

## 2. Methods

### 2.1. Overview

The GBD project quantified measures of health loss for 286 causes of death, 369 diseases and injuries, and 87 risk factors for 204 countries and territories, for both sexes, from 1990 to 2019 [7,8]. GBD 2019 analyses were completed with Python version 3.6.2, Stata version 13, and R version 3.5.0 and complies with the Guidelines for Accurate and Transparent Health Estimates Reporting (GATHER) (Supplementary Material Table S7) [7]. Cause-specific death rates and cause fractions were calculated using the Cause of Death Ensemble model and spatiotemporal Gaussian process regression [7]. Cause-specific deaths were adjusted to match the total all-cause deaths calculated as part of the GBD population, fertility, and mortality estimates [7]. Deaths were multiplied by standard life expectancy at each age to calculate years of life lost (YLL). Prevalence estimates were multiplied by disability weights for mutually exclusive sequelae of diseases and injuries to calculate years lived with disability (YLD) [7]. This study was based on publicly available anonymized databases and, thus, exempt from ethical compliance. Lastly, the table and figures in the present text provide an overview of our main results, while also referring to analyses in the supplemental material to provide greater insight and disaggregated data. A full list of tables and figures in the supplemental material can be found at the end of the main text.

### 2.2. Geographical Unit, Age Groups, Time Periods and Cause Levels

For the present analysis, the geographical unit was at the U.S. state level. In addition, we compared the state of Colorado with the U.S. and West states, including Arizona, California, Idaho, Montana, Nevada, New Mexico, Oregon, Utah, Washington, and Wyoming [3,9]. The GBD diseases and injuries analytical framework generated estimates for each year from 1990 to 2019 [8]. Detailed methods for the GBD 2019 study can be found in the summary publications (Supplemental Material Table S8) [7,8]. Here, we briefly describe our methods for the present analysis, including deaths, YLL, YLD, and DALY; 364 total causes are non-fatal, and 286 are fatal. In this study, we assigned Level 4 causes of DALYs and risk factors in our analysis (the most specific level available from the GBD).

### 2.3. Data

The GBD estimation process uses multiple relevant data sources for each disease or injury, including censuses, household surveys, civil registration and vital statistics, disease registries, health service use, air pollution monitors, satellite imaging, disease notifications, and other sources [7]. For Colorado, the GBD includes 406 data sources from 1921 to 2018, some being the National Health and Nutrition Examination Surveys, state inpatient databases, the National Ambulatory Medical Care Survey, National Hospital Ambulatory Medical Care Survey, Medical Expenditure Panel Survey, National Comorbidity Survey, National Epidemiology Survey On Alcohol And Related Conditions, National Survey On Drug Use And Health, Department Of Agriculture Continuing Survey Food Intakes, National Health Interview Survey, Behavioral Risk Factor Surveillance System, and Centers for Disease Control and Prevention Disease Surveillance Reports [7]. For this sub-analysis, we used data and figures from the 2019 GBD project. Additionally, data and figures were generated by the Institute for Health Metrics and Evaluation (IHME) and are freely available from the IHME website (http://vizhub.healthdata.org/gbd-compare) (accessed on 23 September 2021).

### 2.4. Modelling

Diseases and injuries processed data are modelled using three standardized tools to generate estimates of each quantity of interest by age, sex, location, and year [7]. The first tool, Cause of Death Ensemble model (CODEm), analyzes causes of death data using an ensemble of different modelling methods for rates or cause fractions with varying choices of covariates that perform best with out-of-sample predictive validity [7]. The second tool, spatiotemporal Gaussian process regression (ST-GPR), is a set of regression methods that borrow strength between locations over time for single metrics of interest, such as risk factor exposure or mortality rates [7]. DisMod-MR, the last tool, is a Bayesian meta-regression tool that allows evaluation of all available data on incidence, prevalence, remission, and mortality for a disease, enforcing consistency between epidemiological parameters [7]. GBD 2019 uncertainty intervals (UIs) were generated for every metric using the 25th and 75th ordered draw values of the posterior distribution [7]. Additional GBD 2019 methods and modelling descriptions are in the summary publications and previous studies (Supplemental Material Table S8) [7,10,11,12].

### 2.5. Comparative Risk Assessment

A comparative risk assessment (CRA) approach was included in GBD to attribute health outcomes to risk factors. GBD 2019 includes 87 risk factors grouped in four levels. The CRA in the GBD 2019 is divided into six steps: inclusion of risk–outcome pairs in the analysis; estimation of relative risk as a function of exposure; estimation of exposure levels and distributions; determination of the counterfactual level of exposure, the level of exposure with minimum risk, called the theoretical minimum risk exposure level (TMREL); computation of population attributable fractions and attributable burden; and estimation of mediation of different risk factors through other risk factors [8]. Uncertainty in each step of the GBD 2019 analysis was propagated into the final estimates of attributable burden [7].

## 3. Results

### 3.1. Mortality

**Figure 1:**
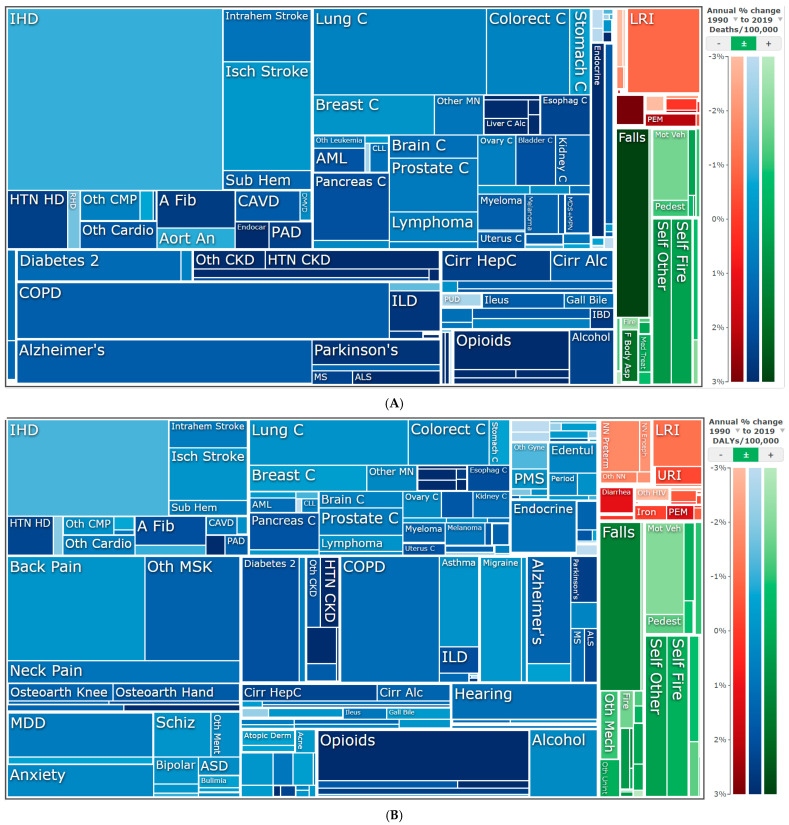
Tree maps of causes of (**A**) death and (**B**) disability-adjusted life years (DALYs) in Colorado, both sexes, all ages, in 2019. Blue rectangles are non-communicable disease; red rectangles are communicable; and green rectangles are injuries. Darker colors represent an increase in disease and the lighter colors show a decrease. The size of the boxes represents overall disease burden (the larger the rectangle, the greater the burden of that disease regardless of increase/decrease). Institute for Health Metrics and Evaluation (IHME). GBD Compare Data Visualization. Seattle, WA: IHME, University of Washington, 2020. Available from http://vizhub.healthdata.org/gbd-compare (accessed on 23 September 2021).

**Figure 2:**
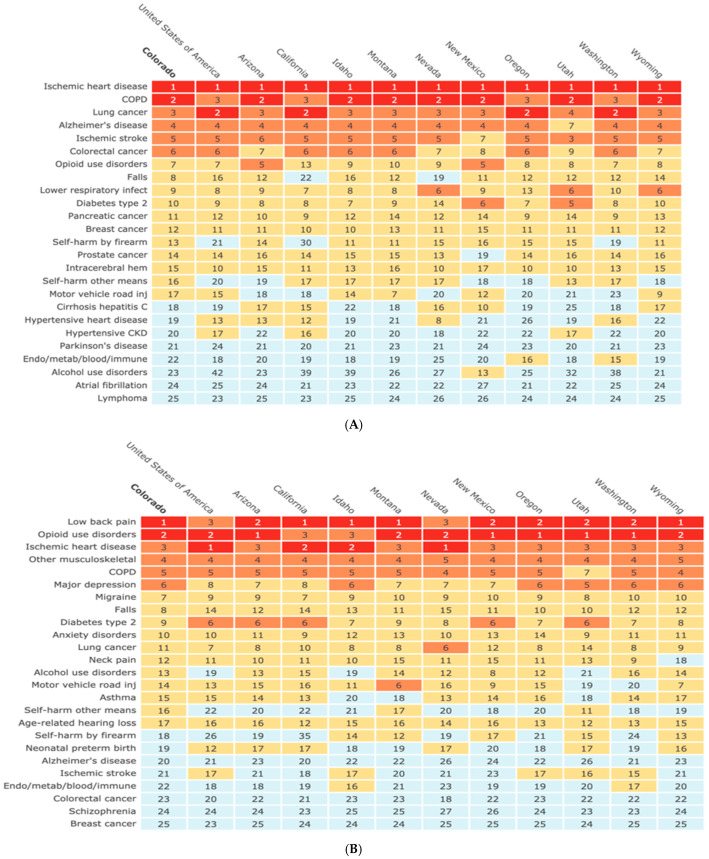
Top-ranked causes of (**A**) death and (**B**) disability-adjusted life years (DALYs) per 100,000, Level 4, age-standardized, in Colorado, West Census States, and the United States, for both sexes, 2019. Numbers are rankings, with colors indicating the scale from low (blue: below 17th) to high (red: first and second) rankings. Institute for Health Metrics and Evaluation (IHME). GBD Compare Data Visualization. Seattle, WA: IHME, University of Washington, 2020. Available from http://vizhub.healthdata.org/gbd-compare (accessed on 23 September 2021).

In 2019, 40,724 (95% uncertainty intervals (UI) 35,134–46,743) deaths were estimated in Colorado (Supplementary Material Table S2 and S6). Estimated deaths among females were 18,806, and 21,919 for males in 2019. Those aged 70 and older accounted for 65% of deaths (Supplementary Material Table S1). Non-communicable diseases accounted for 35,675 deaths, injuries accounted for 3448 deaths, and communicable, maternal, neonatal, and nutritional diseases accounted for 1601 deaths (Figure 1 and Supplementary Material Table S6). From non-communicable diseases, cardiovascular disease represented 28% of total deaths (11,472 deaths), followed by neoplasms with 26% of total deaths (10,462 deaths) (Supplementary Material Table S6). In 2019, the top leading cause of death among all diagnoses in Colorado in both sexes was ischemic heart disease (IHD) (6136 deaths) (Supplementary Material Table S6). Compared with the U.S. and western states, in 2019, Colorado shared the top 5 causes of death—IHD, chronic obstructive pulmonary disease (COPD), lung cancer, Alzheimer’s disease, and Ischemic stroke (Figure 2). Comparative to previous years, when mortality rates are adjusted by age, a reduction in mortality was estimated since 1990 (596 age-standardized mortality per 100,000 in 1990 vs. 494 in 2019).

### 3.2. Disability Adjusted Life Years, Years of Life Lost, and Year Live with Disability

In 2019, 1,676,491 DALYs were lost in Colorado (Supplementary Material Tables S3, S4 and S6). Females reported 804,867 DALYs and males 871,624 DALYs (Supplemental Material Table S4). Those aged 50–69 years accounted for 34% of the total DALYs. Non-communicable diseases accounted for 1,425,347 DALYs (85%), injuries accounted for 183,306 DALYs (11%), and communicable, maternal, neonatal, and nutritional diseases accounted for 67,838 DALYs (4%) (Supplementary Material Table S6). From non-communicable diseases, musculoskeletal disorders represented 14% of total DALYs, followed by neoplasms (Supplementary Material Table S6). From the total DALYs, 49% represented YLL and 51% YLD. Compared with the U.S., in 2019, Colorado ranked low back pain as the number 1 DALYs diagnosis (being 3rd in the U.S.), falls as number 8 (being 14th in the U.S.), and diabetes type 2 as number 9 (being sixth in the U.S. and four other states) (Figure 2). Compared to previous years’ DALY rates adjusted by age, a decrease was estimated since 1990 (27,050 DALYs per 100,000 in 1990 vs. 24,726 in 2019) (Supplemental Material Figure S5). Comparing the percentage of change between 1990 and 2019 of the top 24 causes of DALYs, in both sexes and all ages in Colorado, opioid use disorders showed the largest increase (756%), followed by Diabetes Type 2 (188%), other musculoskeletal disorders (140%), Alzheimer’s disease (142%), and falls (138%); in contrast, motor vehicle road injuries showed a 10% decrease in DALYs during the same period (Table 1).

### 3.3. Risk Factors

**Figure 3:**
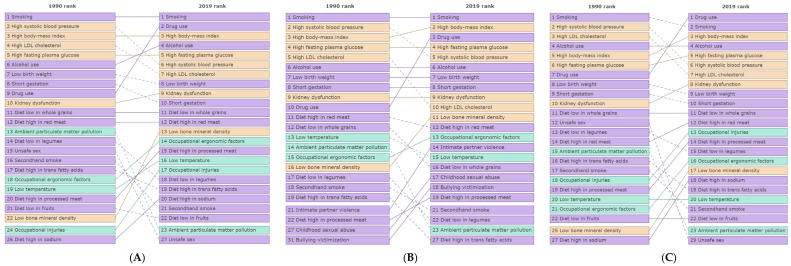
Colorado top risk factors by disability-adjusted life years (DALYs) in (**A**) Both sexes, (**B**) Females, (**C**) Males, Level 4, 1990–2019. Purple rectangles represent behavioral risks; green rectangles represent environmental/occupational risks; and orange rectangles represent metabolic risks. Continous lines represents increase or no change in the rank bewteen years. Dashed line represents decrease in the rank bewteen years. Institute for Health Metrics and Evaluation (IHME). GBD Compare Data Visualization. Seattle, WA: IHME, University of Washington, 2020. Available from http://vizhub.healthdata.org/gbd-compare (accessed on 23 September 2021).

In 2019, the top 6 risk factors by DALYs (age-standardized per 100,000) were smoking, drug use, high BMI, alcohol use, high fasting plasma glucose, and high systolic blood pressure (Figure 3). Comparatively, drug use ranked first for males and third for females (Figure 3, Supplemental Material Table S5 and Figure S19). Alcohol use also ranked fourth for males but sixth for females. In comparison to the U.S., drug use ranked as the number 2 risk factor in Colorado (being third in the U.S.). Compared with western states, alcohol use was ranked fourth in Colorado, while in the rest of the states, it was ranked fifth or sixth. Compared with previous years, drug use is ranked second in 2019 (being 9 in 1990) (DALYs age-standardized per 100,000), high systolic blood pressure is ranked sixth (being 2 in 1990), high LDL cholesterol is ranked seventh (being 4 in 1990), and alcohol use is ranked fourth (being 6 in 1990) (Figure 3).

## 4. Discussion

This is the first study describing health trends from 1990 to 2019 on 286 causes of death, 369 diseases and injuries, and 87 risk factors in Colorado. We found that in 2019, low back pain and IHD were the main causes of health loss, and smoking was the top risk factor. In the last 29 years, Colorado has seen a significant increase in DALYs attributable to opioid use disorder (OUD) and drug use. Overall, most of the mortality and DALYs have resulted from non-communicable diseases. Compared to the U.S. and West States, Colorado has lower rates of mortality and DALY rates than all except California and Washington. Colorado’s population has almost doubled in the last 29 years [5]. Between 1990 and 2000, Colorado experienced an increase in employment, with critical infrastructures built during those years (e.g., Denver international airport, Colorado convention center, Denver light rail) [6]. Between 200 and 2010, similarly to the US, the economic resection impacted Colorado’s economic growth [6]. Finally, between 2010 and 2019, employment increased to pre-recession levels. During those years, natural disasters were registered (flood, wildfires, and drought), especially during 2013, and in 2014, marijuana was legalized for recreational use [6]. In Colorado, health data are available from multiple sources and years. For example, the Colorado Department of Health and Environment (CDPHE) provides multiple datasets on state- and local-level health indicators. For instance, the Center Health and Environment Data (CHED) manages the Colorado Health Information Dataset (CoHID) [13,14]. However, the majority of reports focus on a single health indicator or outcome and only use data collected between 2015 and 2016. Notably, these reports also lack information regarding risk factors attributable to most of the outcomes. Our study describes more up-to-date health information, with greater specificity, a higher number of conditions, and risk factors attributable to health outcomes. Comparatively, the Colorado Health Foundation produced a Colorado Health Report Card measuring 38 health indicators [15]. This report is published annually from 2006 to 2016. The Colorado Health Report Card describes health risks and outcomes across five stages of life (early life, children, adolescents, adults, and elderly), describing indicators such as access to health services, healthy lifestyle (i.e., smoking, physical activity), and a few health outcomes (fertility, mental health, obesity, diabetes, and high blood pressure) [15]. However, this report lacks the inclusion of a broader list of diseases, injuries, risk factors, and health units such as YLL, YLD, or DALYs.

The top three causes of mortality in 2019—IHD, COPD, and lung cancer—have remained the same since 1990. Although, the age-standardized mortality rates in Colorado have steadily decreased since 1990 (Supplemental Material Figure S5). The most notable improvements in mortality among the top 10 causes of deaths since 1990 are in lower respiratory infections (ranked 5th in 1990 and 9th in 2019) and motor vehicle road injuries (ranked 9th in 1990 and 17th in 2019) (Supplemental Material Figure S1). However, there were significant increases in mortality related to OUD (ranked 76th in 1990 and ranked 7th in 2019) and falls (ranked 17th in 1990 and 8th in 2019). The Centers for Disease Control and Prevention suggested a set of tools to reduce opioid exposure and promote OUD prevention. For instance, increased prescription drug monitoring programs, state prescription laws, insurance opioid management programs, education and implementation of opioid guidelines, patient education, and community awareness [16].

Comparatively, both sexes share the top two causes of death—IHD and COPD (Supplemental Material Figure S1). In females, Alzheimer’s ranked 3rd and lung cancer 4th, while in males, it is the opposite. Males have higher mortality related to OUD (ranked 6th in males and 10 in females) (Supplemental Material Figure S1). Likewise, in males, self-harm by firearm ranked 7th as a cause of death, while in females, it was not ranked among the top 25. Females have higher mortality related to ischemic stroke (ranked 5th in females and 9th in males) (Supplemental Material Figure S1). Overall, non-communicable diseases are the top causes of mortality in Colorado, and primary prevention should focus on population- and individual-level interventions [17]. Population-level interventions should focus on tobacco control, support of healthy diets (based on fruits, vegetables, legumes, and nuts), a reduction in unhealthy foods (such as saturated fats, trans fats, refined carbohydrates, excessive salt, and alcohol), promotion of physical activity (leisure, transport, and work), and control of air pollution [17]. At the individual level, identifying those with multifactorial risks and implementing guideline-driven management of hypertension, low-density lipoproteins cholesterol, and diabetes should be prioritized [17].

In Colorado, between 1990 and 2012, DALY rates steadily decreased. However, since 2012, DALYs gradually increased (23,685 DALYs per 100,000 in 2012 vs. 24,726 in 2019), with a similar pattern across the country (Supplemental Material Figure S5). The five leading diseases in 2019 were low back pain, OUD, IHD, other musculoskeletal disorders, and COPD (Supplemental Material Figure S4). There were significant improvements in lung cancer (ranked 4th in 1990 and 11 in 2019) and motor vehicle road injuries (ranked 5th in 1990 and 14 in 2019) (Supplemental Material Figure S4). In contrast, since 1990, low back pain and IHD remained in the top two causes of disability (Supplemental Material Figure S4). Notably, DALYs attributable to OUD have significantly increased over time (ranked 28th in 1990 and 2 in 2019), following U.S.-wide trends (Supplemental Material Figure S4) [3]. Broken down by sex, low back pain is ranked first in females (being 3 in males), and IHD is ranked first in males (being 7 in females) (Supplemental Material Figure S4). As with mortality, self-harm by firearm is also ranked 7th in DALYS among males, mainly driven by YLL, but among females remains outside of the top 25 causes of DALYs (Supplemental Material Figure S4). Likewise, anxiety disorder is ranked 8th in females, mainly derived by YLD, and ranked 18th in males (Supplemental Material Figure S4). These findings allude to the potential benefits of using sex-specific health interventions and policy. The top 5 burdens attributable to YLDs are low back pain, other musculoskeletal disorders, major depression, OUD, and migraine (Supplemental Material Figure S13). In contrast, the top 6 burdens attributable to YLL are IHD, COPD, lung cancer, OUD, and self-harm (Supplemental Material Figure S13). These findings highlight the need for policies and services targeting cardiorespiratory, musculoskeletal, and drug use disorders in Colorado. To reduce DALYs in Colorado, interventions should focus on those populations and personal interventions to reduce cardiovascular, metabolic, and respiratory diseases, as mentioned before. In addition, musculoskeletal disorders, such as low back pain, require special attention because they are very prevalent in Colorado. Despite many clinical guidelines for managing low back pain, there is still a gap between evidence and practice [18]. The promotion of fundamental principles to prevent and reduce the musculoskeletal-related health burden needs to increase across interventions—for instance, prescribing specific exercises to strengthen the entire back, reducing excess weight and obesity, supporting diets rich in calcium and vitamin D, and increasing daily physical activity [19]. However, without the collaborative efforts of people with low back pain, policymakers, clinicians, and researchers necessary to develop and implement effective solutions, disability rates and expenditure for low back pain will continue to rise [18].

The top 6 leading risk factors in Colorado in 2019 were smoking, drug use, high BMI, alcohol use, high fasting plasma glucose, and high blood pressure, with similar rankings in males and females (Supplemental Material Figure S22). Among risk factors attributable to DALYs, broken down by sex, smoking has ranked first in females since 1990, whereas it has decreased to second in males (Supplemental Material Figure S19). Notably, drug use has significantly increased in both sexes, where it ranked first in males in 2019 (being 7 in 1990) and third in females (being 10 in 1990). Physicians play a fundamental role in substance use prevention and addiction control by providing counseling and education for patients who have been prescribed medication with addictive properties [3]. Furthermore, increasing drug checking (to detect fentanyl) and harm reduction services could help prevent individuals from an overdose [20]. High BMI, high fasting plasma glucose, and high blood pressure pose considerable risks to health. Strategies to mitigate these risks include providing access to effective programs that enhance physical activity, modifying dietary behaviors and increasing taxation on sugary drinks to reduce consumption [3]. For prevention interventions targeting smoking, there has been success in implementing increased access to community-based cessation programs, health promotion campaigns, and peer education [21]. Lastly, when designing prevention programs, a better understanding of smoking behavior and social circumstance and social determinants of health should be considered [21].

### Limitations

This study has several limitations to be considered. A major limitation of the GBD analysis is the availability and accuracy of primary data by time and state [3,7]. When primary data are unavailable, results depend on the out-of-sample predictive validity of the modelling efforts [7]. The GBD 2019 statistical modelling was designed to capture uncertainty; however, it remains a challenge to fully represent UIs around estimates in locations where data are sparse [7]. Overall, the assessment of burden attributable to all risks combined and risk-deleted mortality is limited by several potentially important risk factors not included in this analysis. Generally, relative risks are assumed as a function of exposure and apply in all locations and periods, with the exceptions of temperature and high BMI for breast cancer [8]. This study did not include an analysis of the impact COVID-19 related to our findings as data are not yet available for consideration. However, we expect that the Colorado burden of disease will change in the coming years due to the pandemic. To better manage the consequences of the pandemic, we recommend using the baseline health outcome and risk factor data presented in this paper to inform future interventions. Lastly, the scope of this study did not include intrastate level health trends due to the lack of available and up-to-date spatially disaggregated data.

## 5. Conclusions

Non-communicable diseases and their risk factors are the top leading causes of DALYs in Colorado. DALYs attributable to opioid use disorder have significantly increased between 1990 and 2019, while low back pain and IHD have also persisted as the top three causes of DALYs among those years. While mortality rates in Colorado have decreased over time, IHD remains the leading cause of death among both sexes. Findings support the need for policies to prevent non-communicable diseases, with particular attention to musculoskeletal disorders and interventions to reduce tobacco, alcohol, and drug use. Continuous collaboration between public health practitioners, physicians, and other health personnel is needed to support health promotion, prevention, and care services. Furthermore, health practitioners should increase collaboration with non-health sectors to implement a “health in all policies” approach to reduce the disease burden in Colorado.

## Figures and Tables

**Table 1:** Top 24 causes of disability-adjusted life years (DALYs) in both sexes, females and males, all ages, in Colorado, 1990 and 2019.

Cause	DALYs 1990			DALYs 2019			Percentage of Change 1990 vs. 2019 (%)
	Both Sexes	Females	Males	Both Sexes	Females	Males	
Age-related hearing loss	15,423 (10,751–21,602)	7218 (5041–9888)	8205 (5713–11,701)	34,146 (23,592–47,584)	15,221 (10,629–21,000)	18,925 (12,943–26,941)	121
Alcohol use disorders	17,103 (13,025–22,501)	6187 (4522–8251)	10,916 (8358–14,256)	29,805 (23,794–36,652)	11,261 (8630–14,351)	18,545 (14,608–22,913)	74
Alzheimer’s disease	12,448 (5902–26,406)	8394 (4002–17,638)	4054 (1946–8687)	30,160 (14,435–62,129)	18,381 (8852–38,254)	11,780 (5638–24,702)	142
Anxiety disorders	17,734 (12,395–24,048)	11,366 (7818–15,395)	6369 (4420–8832)	31,254 (22,185–42,289)	19,764 (13,991–27,078)	11,491 (8026–15,834)	76
Asthma	14,251 (9920–20,180)	8050 (5575–11,259)	6202 (4260–8860)	23,132 (15,879–32,359)	13,049 (8979–18,395)	10,084 (6862–14,199)	62
Breast cancer	13,768 (12,769–14,822)	13,678 (12,678–14,731)	91 (77–106)	19,741 (15,783–24,428)	19,530 (15,599–24,175)	212 (154–280)	43
Colorectal cancer	13,071 (12,364–13,832)	6390 (5923–6865)	6682 (6261–7130)	23,701 (19,899–28,208)	10,329 (8237–12,819)	13,373 (10,429–17,165)	81
COPD	35,939 (33,068–38,442)	16,441 (14,409–18,231)	19,498 (18,028–20,944)	80,742 (68,865–91,749)	39,393 (30,426–46,855)	41,349 (33,941–49,782)	125
Diabetes type 2	16,629 (13,688–20,125)	8435 (6963–10,200)	8195 (6613–9949)	47,861 (37,370–60,056)	20,503 (15,612–26,109)	27,359 (21,089–34,658)	188
Endocrine/metabolic/blood/immune disorders	10,234 (7684–13,297)	6668 (4843–8801)	3566 (2803–4685)	21,792 (17,183–27,070)	12,571 (9587–15,793)	9221 (6991–11,940)	113
Falls	19,299 (15,030–24,756)	9130 (6917–11,974)	10,169 (8081–12,997)	46,008 (37,093–57,843)	23,613 (18,709–29,851)	22,396 (17,734–27,830)	138
Ischemic heart disease	87,902 (82,994–91,558)	33,578 (30,555–35,838)	54,325 (51,631–56,598)	100,107 (83,064–119,454)	33,271 (26,829–41,178)	66,836 (52,135–84,824)	14
Ischemic stroke	17,453 (15,535- 19,348)	11,052 (9647–12,408)	6401 (5777–7025)	27,625 (23,057–32,013)	16,371 (13,447–19,280)	11,255 (9089–13,461)	58
Low back pain	58,862 (41,350–79,287)	32,837 (23,060–44,165)	26,025 (18,112–35,301)	92,305 (66,704–121,026)	51,220 (37,086–66,925)	41,086 (29,329–54,217)	57
Lung cancer	28,840 (27,591–30,128)	10,872 (10,151–11,629)	17,969 (17,006–18,938)	46,615 (38,977–55,043)	21,560 (16,811–27,006)	25,055 (19,244–32,111)	62
Major depression	21,482 (14,589–29,856)	13,463 (9110–18,590)	8020 (5466–11,072)	42,761 (29,523–58,544)	27,620 (19,080–37,775)	15,142 (10,364–21,070)	99
Migraine	22,590 (3284–51,015)	15,465 (1952–36,015)	7125 (1336–15,917)	34,619 (5618–78,169)	23,615 (3305–54,196)	11,004 (2269–24,536)	53
Motor vehicle road injuries	27,170 (25,683–28,908)	9899 (9195–10,735)	17,272 (16,240–18,415)	24,449 (20,578–28,670)	8991 (7326–10,895)	15,458 (12,366–18,882)	-10
Neck pain	15,719 (10,409–22,598)	9226 (6199–13,353)	6494 (4237–9281)	33,263 (22,564–47,117)	19,796 (13,344–28,136)	13,468 (9167–19,026)	112
Opioid use disorders	8400 (6290–11,046)	3597 (2593–4999)	4804 (3648–6208)	71,887 (57,703–88,063)	28,515 (21,488–36,371)	43,373 (34,325–53,513)	756
Other musculoskeletal disorders	25,844 (18,288–35,284)	16,267 (11,550–21,728)	9578 (6554–13,415)	64,338 (45,677–85,903)	39,225 (27,818–51,968)	25,114 (17,785–33,945)	149
Prostate cancer	7417 (5319–8675)	0	7417 (5319–8675)	14,765 (11,147–22,461)	0	14,765 (11,147–22,461)	99
Self-harm by firearm	14,986 (13,945–17,635)	2194 (2005–2395)	12,793 (11,791–15,469)	23,009 (18,001–29,923)	2912 (2158–3745)	20,098 (15,187–26,985)	54
Self-harm by other means	13,508 (10,725–14,560)	4028 (3713–4345)	9480 (6969–10,436)	24,207 (17,702–29,322)	8178 (6240–10,383)	16,030 (10,224–20,888)	79

## Data Availability

Data and figures were generated by the Institute for Health Metrics and Evaluation (IHME) and are freely available from the IHME website (http://vizhub.healthdata.org/gbd-compare, accessed on 23 September 2021).

## Supplementary Materials

The following supporting information can be downloaded at: https://www.mdpi.com/article/10.3390/ijerph19010288/s1, Table S1: Main causes of death in Colorado in, both sexes, Level 4, by age group, in 2019; Table S2: All-cause mortality estimates for Colorado and the United States, in 1990, 2000, 2010, 2019; Figure S1: Arrow diagram of top ranked causes of death, in Colorado, in (A) both sexes (B) females and (C) males, Level 4, age standardized, 1990–2019; Figure S2: Heat map of top causes of death in Colorado, West States, and the United States, for both sexes, Level 4, age-standardized, in 1990. Numbers are rankings, with colors indicating the scale from low (blue: below 17th) to high (red: first and second) rankings; Figure S3. Heat map of top causes of death in Colorado, West States, and the United States, for (A) females and (B) males, Level 4, age-standardized, in 2019. Numbers are rankings, with colors indicating the scale from low (blue: below 17th) to high (red: first and second) rankings; Table S3: Colorado disability adjusted life years (DALYs), by age group and in all ages, in 2019; Table S4: Main causes of disability-adjusted life-years (DALYs) for Colorado in, females and males, all ages, in 2019; Figure S4: Arrow diagram of top ranked causes of disability adjusted life years (DALYs), in Colorado, in (A) both sexes (B) females and (C) males, Level 4, age standardized, 1990–2019; Figure S5: Line graph of age-standardized rates of (A) death and (B) disability adjusted life years (DALYs), per 100,000 people, in Colorado, West States, and the United States, in both sexes, 1990-2019; Figure S6: Line graph of disability adjusted life years (DALYs), in Colorado, West States, and the United States by (A) females and (B) males, Level 4, age standardized, 1990–2019; Figure S7: Heat map of top ranked causes of disability-adjusted life years (DALYS) in Colorado, West States, and the United States for (A) females (B) males, Level 4, age standardized, in 2019.Numbers are rankings, with colors indicating the scale from low (blue: below 17th) to high (red: first and second) rankings.; Figure S8: Heat map of top ranked causes of disability-adjusted life years (DALYS) in colorado, West States, and the United States, for both sexes, Level 4, age standardized, in 1990.Numbers are rankings, with colors indicating the scale from low (blue: below 17th) to high (red: first and second) rankings.; Figure S9: Pyramid of disability adjusted life years (DALYs) in Colorado and the United States, by sex and all ages in 2019; Figure S10: Pyramid of disability adjusted life years (DALYs) in Colorado, Earth and the United States, by sex and all ages in 2019; Figure S11: Heat map of top ranked years of life lost (YLL) in Colorado, West states, and the United States in (A) both sexes (B) females and (C) males, Level 4, age standardized, in 2019.Numbers are rankings, with colors indicating the scale from low (blue: below 17th) to high (red: first and second) rankings; Figure S12: Heat map of top ranked years of life lost (YLL) in Colorado, West States, and the United States, both sexes, Level 4, age standardized in 1990.Numbers are rankings, with colors indicating the scale from low (blue: below 17th) to high (red: first and second) rankings; Figure S13: Tree map of top causes of (A) years of healthy life lost due to disability (YLDs) and (B) years of life lost (YLLs) in Colorado, for both sexes, Level 4, all ages, in 2019.Blue rectangles are non-communicable disease; red rectangles are communicable; and green rectangles are injuries; Figure S14: Heat map of top ranked causes of years of healthy life lost due to disability (YLDs) per 100,000 people, in Colorado, West States, and the United States for (A) both sexes, (B) females and (C) males, Level 4, age-standardized, in 2019. Numbers are rankings, with colors indicating the scale from low (blue: below 17th) to high (red: first and second) rankings; Figure S15: Heat of top ranked years lived with disability (YLD) in Colorado, West States, and the United States, both sexes, Level 4, age standardized in 1990. Numbers are rankings, with colors indicating the scale from low (blue: below 17th) to high (red: first and second) rankings; Figure S16: Top ranked deaths per 100,000 people attributable to major risk factors in Colorado, both sexes, Level 4, age-standardized, in 2019; Figure S17: Arrow diagram of top risk factors for (A) deaths and (B) disability-adjusted life years (DALYs) per 100,000 people in Colorado, both sexes, Level 4, age-standardized, 1990-2019; Figure S18: Arrow diagram of top ranked risk factors for deaths in Colorado, in (A) females and (B) males, Level 4, age-standardized, in 2019; Figure S19: Arrow diagram of top ranked risk factors contributing to disability adjusted life years (DALYs) in Colorado, in (A) females and (B) males, Level 4, age-standardized, 2019; Figure S20: Top ranked risk factors contributing to disability adjusted life years (DALYs) in percent in Colorado by (A) both sexes (B) female (C) males, Level 4, age-standardized, in 2019; Figure S21: Top ranked risk factors contributing to disability-adjusted life years (DALYs) per 100,000 people, attributable to major risk factors in Colorado in (A) females and (B) males, Level 4, age standardized, in 2019; Table S5: Colorado age-standardized summary exposure values (SEV), for both sexes, in 1990, 2000, 2010, 2019; Figure S22: Heat map of top ranked risk factors in Colorado, West States, and the United States in (A) both sexes (B) females and (C) males, Level 4, age standardized, in 2019. *Numbers are rankings, with colors indicating the scale from low (blue: below 17th) to high (red: first and second) rankings; Figure S23: Heat map of top ranked risk factors in Colorado, West States, and the United States, both sexes, Level 4, age standardized, in 1990. Numbers are rankings, with colors indicating the scale from low (blue: below 17th) to high (red: first and second) rankings; Figure S24: Arrow diagram of top risk factors for years of life lived with disability (YLDs) per 100,000 people, in Colorado in (A) both, (B) females, (C) males, Level 4, 1990–2019; Figure S25: Arrow diagram of top risk factors for years of life lost (YLLs) per 100,000 people in Colorado in (A) both sexes, (B) females, and (C) males, Level 4, 1990–2019; Table S6: Main causes of death and disability-adjusted life-years (DALYs), both sexes and all ages, Colorado, 2019; Table S7: GATHER checklist; Table S8: Key Global Burden of Disease (GBD) papers and sources used as a reference in this analysis.

## Abbreviation

A Fib	Atrial fibrillation and flutter
ALS	Motor neuron disease
AML	Acute myeloid leukemia
Aort An	Aortic aneurysm
Bladder C	Bladder cancer
Brain C	Brain and central nervous system cancer
Breast C	Breast Cancer
CAVD	Non-rheumatic calcific aortic valve disease
CDPHE	Colorado Department of Public Health and Environment
CHED	Center Health and Environment Data
CLL	Chronic lymphoid leukemia
CODEm	Cause of Death Ensemble model
CoHID	Colorado Health Information Dataset
Colorect C	Colon and rectum cancer
COPD	Chronic obstructive pulmonary disease
CRA	Comparative risk assessment
DALY	Disability-adjusted life year
Endocar	Endocarditis
Esophag C	Esophageal cancer
GATHER	Guidelines for Accurate and Transparent Health Estimates Reporting
GBD	Global Burden of Disease
HTN CKD	Chronic kidney disease due to hypertension
HTN HD	Hypertensive heart disease
IHD	Ischemic heart disease
IHME	Institute for Health Metrics and Evaluation
Intrahem Stroke	Intracerebral Stroke
Isch stroke	Ischemic Stroke
Kidney C	Kidney cancer
LRI	Lower respiratory infections
Lung C	Tracheal, bronchus, and lung cancer
Mot Veh	Motor vehicle road injuries
MS	Multiple sclerosis
Oth Cardio	Other cardiovascular and circulatory diseases
Oth CKD	Chronic kidney disease due to other and unspecified causes
Oth Neuro	Other neurological disorders
Other CMP	Other cardiomyopathy
Other MN	Other malignant neoplasms
OUD	Opioid Use Disorder
Ovary C	Ovarian cancer
PAD	Peripheral artery disease
Pancreas C	Pancreatic cancer
Self-Fire	Self-harm by firearm
Self-Other	Self-harm by other specified means
ST-GPR	Spatiotemporal Gaussian process regression
Stomach C	Stomach Cancer
Sub Hem	Subarachnoid hemorrhage
TMREL	Theoretical minimum risk exposure level
UI	Uncertainty Intervals
US	United States
YLD	Years of life lived with disability
YLL	Years of life lost
