# Supplementary material for: Colorado Burden of Disease, Injuries and Risk Factors, 1990–2019: A Sub-Analysis of the Global Burden of Disease Study
Source: Int J Environ Res Public Health. 2021 Dec 28;19(1):288. doi: 10.3390/ijerph19010288 (PMC8744865; doi:10.3390/ijerph19010288)
Supplement: Supplementary file 1 [file ijerph-19-00288-s001.zip › ijerph-1294865-supplementary.pdf]

SUPPLEMENTAL MATERIAL

# Colorado Burden of Disease, Injuries and Risk Factors, 1990-2019: a sub-analysis of the Global Burden of Disease Study

Jen Roux MPH [a,b], David Rojas-Rueda PhD [a,b]

[a] Colorado School of Public Health, Colorado State University, Fort Collins, U.S.A.

[b] Department of Environmental and Radiological Health Sciences, Colorado State University, Fort Collins, U.S.A.

# CONTENTS

**Table S1.** Main causes of death in Colorado in, both sexes, Level 4, by age group, in 2019 ....4

**Table S2.** All-cause mortality estimates for Colorado and the United States, in 1990, 2000, 2010, 2019 ....10

**Figure S1.** Arrow diagram of top ranked causes of death, in Colorado, in (A) both sexes (B) females and (C) males, Level 4, age standardized, 1990 - 2019 ....11

**Figure S2.** Heat map of top causes of death in Colorado, West States, and the United States, for both sexes, Level 4, age-standardized, in 1990. *Numbers are rankings, with colors indicating the scale from low (blue: below 17th) to high (red: first and second) rankings. ....13

**Figure S3.** Heat map of top causes of death in Colorado, West States, and the United States, for (A) females and (B) males, Level 4, age-standardized, in 2019. *Numbers are rankings, with colors indicating the scale from low (blue: below 17th) to high (red: first and second) rankings....14

**Table S3.** Colorado disability adjusted life years (DALYs), by age group and in all ages, in 2019 ....15

**Table S4.** Main causes of disability-adjusted life-years (DALYs) for Colorado in, females and males, all ages, in 2019 .16

**Figure S4.** Arrow diagram of top ranked causes of disability adjusted life years (DALYs), in Colorado, in (A) both sexes (B) females and (C) males, Level 4, age standardized, 1990 - 2019 ....20

**Figure S5.** Line graph of age-standardized rates of (A) death and (B) disability adjusted life years (DALYs), per 100,000 people, in Colorado, West States, and the United States, in both sexes, 1990-2019 ....22

**Figure S6.** Line graph of disability adjusted life years (DALYs), in Colorado, West States, and the United States by (A) females and (B) males, Level 4, age standardized, 1990 - 2019 ....23

**Figure S7.** Heat map of top ranked causes of disability-adjusted life years (DALYS) in Colorado, West States, and the United States for (A) females (B) males, Level 4, age standardized, in 2019.*Numbers are rankings, with colors indicating the scale from low (blue: below 17th) to high (red: first and second) rankings. ....24

**Figure S8.** Heat map of top ranked causes of disability-adjusted life years (DALYS) in Colorado, West States, and the United States, for both sexes, Level 4, age standardized, in 1990.*Numbers are rankings, with colors indicating the scale from low (blue: below 17th) to high (red: first and second) rankings....25

**Figure S9.** Pyramid of disability adjusted life years (DALYs) in Colorado and the United States, by sex and all ages in 2019....26

**Figure S10.** Pyramid of disability adjusted life years (DALYs) in Colorado, Earth and the United States, by sex and all ages in 2019 ....27

**Figure S11.** Heat map of top ranked years of life lost (YLL) in Colorado, West states, and the United States in (A) both sexes (B) females and (C) males, Level 4, age standardized, in 2019.*Numbers are rankings, with colors indicating the scale from low (blue: below 17th) to high (red: first and second) rankings....28

**Figure S12.** Heat map of top ranked years of life lost (YLL) in Colorado, West States, and the United States, both sexes, Level 4, age standardized in 1990.*Numbers are rankings, with colors indicating the scale from low (blue: below 17th) to high (red: first and second) rankings....30

**Figure S13.** Tree map of top causes of (A) years of healthy life lost due to disability (YLDs) and (B) years of life lost (YLLs) in Colorado, for both sexes, Level 4, all ages, in 2019.*Blue rectangles are non-communicable disease; red rectangles are communicable; and green rectangles are injuries. ....31

**Figure S14.** Heat map of top ranked causes of years of healthy life lost due to disability (YLDs) per 100,000 people, in Colorado, West States, and the United States for (A) both sexes, (B) females and (C) males, Level 4, age-standardized,

in 2019. *Numbers are rankings, with colors indicating the scale from low (blue: below $17^{th}$) to high (red: first and second) rankings. ....32

**Figure S15.** Heat of top ranked years lived with disability (YLD) in Colorado, West States, and the United States, both sexes, Level 4, age standardized in 1990. *Numbers are rankings, with colors indicating the scale from low (blue: below $17^{th}$) to high (red: first and second) rankings. ....34

**Figure S16.** Top ranked deaths per 100,000 people attributable to major risk factors in Colorado, both sexes, Level 4, age-standardized, in 2019 ....35

**Figure S17.** Arrow diagram of top risk factors for (A) deaths and (B) disability-adjusted life years (DALYs) per 100,000 people in Colorado, both sexes, Level 4, age-standardized, 1990-2019 ....36

**Figure S18.** Arrow diagram of top ranked risk factors for deaths in Colorado, in (A) females and (B) males, Level 4, age-standardized, in 2019 ....37

**Figure S19.** Arrow diagram of top ranked risk factors contributing to disability adjusted life years (DALYs) in Colorado, in (A) females and (B) males, Level 4, age-standardized, 2019 ....38

**Figure S20.** Top ranked risk factors contributing to disability adjusted life years (DALYs) in percent in Colorado by (A) both sexes (B) female (C) males, Level 4, age-standardized, in 2019 ....39

**Figure S21.** Top ranked risk factors contributing to disability-adjusted life years (DALYs) per 100,000 people, attributable to major risk factors in Colorado in (A) females and (B) males, Level 4, age standardized, in 2019 ....41

**Table S5.** Colorado age-standardized summary exposure values (SEV), for both sexes, in 1990, 2000, 2010, 2019 ....42

**Figure S22.** Heat map of top ranked risk factors in Colorado, West States, and the United States in (A) both sexes (B) females and (C) males, Level 4, age standardized, in 2019. *Numbers are rankings, with colors indicating the scale from low (blue: below $17^{th}$) to high (red: first and second) rankings. ....44

**Figure S23.** Heat map of top ranked risk factors in Colorado, West States, and the United States, both sexes, Level 4, age standardized, in 1990. *Numbers are rankings, with colors indicating the scale from low (blue: below $17^{th}$) to high (red: first and second) rankings. ....46

**Figure S24.** Arrow diagram of top risk factors for years of life lived with disability (YLDs) per 100,000 people, in Colorado in (A) both, (B) females, (C) males, Level 4, 1990 - 2019 ....47

**Figure S25.** Arrow diagram of top risk factors for years of life lost (YLLs) per 100,000 people in Colorado in (A) both sexes, (B) females, and (C) males, Level 4, 1990 – 2019 ....49

**Table S6.** Main causes of death and disability-adjusted life-years (DALYs), both sexes and all ages, Colorado, 2019. ..51

**Table S7.** GATHER checklist ....57

**Table S8.** Key Global Burden of Disease (GBD) papers and sources used as a reference in this analysis ....58

**Table S1.** Main causes of death in Colorado in, both sexes, Level 4, by age group, in 2019

| Cause | Deaths | | | | | | | | | | | | | | |
|---|---|---|---|---|---|---|---|---|---|---|---|---|---|---|---|
| | Under 5 | | | 5-14 years | | | 15-49 years | | | 50-69 years | | | 70+ years | | |
| | Central Value | Lower Uncertainty Level | Upper Uncertainty Level | Central Value | Lower Uncertainty Level | Upper Uncertainty Level | Central Value | Lower Uncertainty Level | Upper Uncertainty Level | Central Value | Lower Uncertainty Level | Upper Uncertainty Level | Central Value | Lower Uncertainty Level | Upper Uncertainty Level |
| Acute hepatitis A | 0 | 0 | 0 | 0 | 0 | 0 | 0 | 0 | 0 | 0 | 0 | 0 | 0 | 0 | 0 |
| Acute hepatitis B | 0 | 0 | 0 | 0 | 0 | 0 | 0 | 0 | 0 | 0 | 0 | 0 | 0 | 0 | 0 |
| Acute hepatitis C | 0 | 0 | 0 | 0 | 0 | 0 | 0 | 0 | 0 | 0 | 0 | 0 | 0 | 0 | 0 |
| Acute hepatitis E | 0 | 0 | 0 | 0 | 0 | 0 | 0 | 0 | 0 | 0 | 0 | 0 | 0 | 0 | 0 |
| Acute lymphoid leukemia | 1 | 1 | 1 | 2 | 2 | 3 | 8 | 6 | 10 | 8 | 6 | 11 | 10 | 7 | 13 |
| Acute myeloid leukemia | 1 | 1 | 1 | 1 | 1 | 2 | 16 | 12 | 21 | 64 | 49 | 81 | 122 | 93 | 154 |
| Alcoholic cardiomyopathy | 0 | 0 | 0 | 0 | 0 | 0 | 9 | 6 | 13 | 35 | 23 | 52 | 20 | 12 | 31 |
| Amphetamine use disorders | 0 | 0 | 0 | 0 | 0 | 0 | 48 | 37 | 62 | 17 | 12 | 25 | 1 | 0 | 1 |
| Anorexia nervosa | 0 | 0 | 0 | 0 | 0 | 0 | 1 | 0 | 1 | 0 | 0 | 0 | 0 | 0 | 0 |
| Asbestosis | 0 | 0 | 0 | 0 | 0 | 0 | 0 | 0 | 0 | 1 | 1 | 2 | 10 | 6 | 13 |
| Ascariasis | 0 | 0 | 0 | 0 | 0 | 0 | 0 | 0 | 0 | 0 | 0 | 0 | 0 | 0 | 0 |
| Bulimia nervosa | 0 | 0 | 0 | 0 | 0 | 0 | 0 | 0 | 1 | 0 | 0 | 0 | 0 | 0 | 0 |
| Cellulitis | 0 | 0 | 0 | 0 | 0 | 0 | 3 | 1 | 5 | 12 | 5 | 18 | 34 | 15 | 54 |
| Chlamydial infection | 0 | 0 | 0 | 0 | 0 | 0 | 0 | 0 | 0 | 0 | 0 | 0 | 0 | 0 | 0 |
| Chronic kidney disease | 1 | 0 | 1 | 0 | 0 | 0 | 35 | 27 | 44 | 250 | 196 | 315 | 976 | 785 | 1160 |
| Chronic kidney disease due to diabetes mellitus type 1 | 0 | 0 | 0 | 0 | 0 | 0 | 3 | 1 | 5 | 10 | 4 | 20 | 11 | 5 | 20 |
| Chronic kidney disease due to diabetes mellitus type 2 | 0 | 0 | 0 | 0 | 0 | 0 | 5 | 2 | 8 | 78 | 47 | 113 | 258 | 176 | 358 |
| Chronic kidney disease due to glomerulonephritis | 0 | 0 | 0 | 0 | 0 | 0 | 12 | 8 | 17 | 39 | 22 | 62 | 77 | 48 | 118 |
| Chronic kidney disease due to hypertension | 0 | 0 | 0 | 0 | 0 | 0 | 6 | 4 | 9 | 76 | 52 | 107 | 462 | 346 | 594 |
| Chronic kidney disease due to other and unspecified causes | 0 | 0 | 1 | 0 | 0 | 0 | 9 | 6 | 14 | 47 | 26 | 73 | 169 | 108 | 240 |
| Chronic lymphoid leukemia | 0 | 0 | 0 | 0 | 0 | 0 | 1 | 1 | 1 | 19 | 13 | 26 | 69 | 52 | 90 |
| Chronic myeloid leukemia | 0 | 0 | 0 | 0 | 0 | 0 | 2 | 2 | 3 | 6 | 4 | 9 | 16 | 12 | 21 |

| | | | | | | | | | | | | | | | |
|---|---|---|---|---|---|---|---|---|---|---|---|---|---|---|---|
| Cirrhosis and other chronic liver diseases due to alcohol use | 0 | 0 | 0 | 0 | 0 | 0 | 41 | 29 | 56 | 191 | 135 | 260 | 79 | 58 | 104 |
| Cirrhosis and other chronic liver diseases due to hepatitis B | 0 | 0 | 0 | 0 | 0 | 0 | 6 | 4 | 8 | 16 | 11 | 22 | 8 | 6 | 11 |
| Cirrhosis and other chronic liver diseases due to hepatitis C | 0 | 0 | 0 | 0 | 0 | 0 | 99 | 77 | 127 | 305 | 226 | 404 | 138 | 105 | 173 |
| Cirrhosis and other chronic liver diseases due to NAFLD | 0 | 0 | 0 | 0 | 0 | 0 | 15 | 8 | 24 | 55 | 31 | 89 | 38 | 23 | 56 |
| Cirrhosis and other chronic liver diseases due to other causes | 0 | 0 | 0 | 0 | 0 | 0 | 25 | 16 | 38 | 91 | 56 | 138 | 82 | 53 | 116 |
| Coal workers pneumoconiosis | 0 | 0 | 0 | 0 | 0 | 0 | 0 | 0 | 0 | 1 | 0 | 1 | 3 | 2 | 5 |
| Cocaine use disorders | 0 | 0 | 0 | 0 | 0 | 0 | 83 | 65 | 106 | 37 | 26 | 51 | 2 | 1 | 2 |
| Congenital heart anomalies | 18 | 14 | 25 | 2 | 2 | 3 | 13 | 10 | 16 | 7 | 5 | 10 | 0 | 0 | 0 |
| Congenital musculoskeletal and limb anomalies | 3 | 2 | 4 | 0 | 0 | 0 | 1 | 0 | 1 | 1 | 0 | 1 | 0 | 0 | 0 |
| Cyclist road injuries | 0 | 0 | 0 | 2 | 1 | 2 | 8 | 6 | 10 | 8 | 6 | 11 | 2 | 2 | 3 |
| Diabetes mellitus type 1 | 0 | 0 | 0 | 0 | 0 | 1 | 24 | 19 | 30 | 20 | 14 | 29 | 13 | 9 | 20 |
| Diabetes mellitus type 2 | 0 | 0 | 0 | 0 | 0 | 0 | 32 | 24 | 41 | 265 | 212 | 326 | 544 | 446 | 640 |
| Digestive congenital anomalies | 5 | 3 | 7 | 0 | 0 | 0 | 0 | 0 | 1 | 0 | 0 | 1 | 0 | 0 | 0 |
| Down syndrome | 1 | 1 | 2 | 0 | 0 | 0 | 3 | 2 | 5 | 11 | 8 | 14 | 0 | 0 | 0 |
| Drug-susceptible tuberculosis | 0 | 0 | 0 | 0 | 0 | 0 | 1 | 1 | 2 | 4 | 3 | 5 | 7 | 5 | 8 |
| Ectopic pregnancy | | | | 0 | 0 | 0 | 0 | 0 | 0 | 0 | 0 | 0 | 0 | 0 | 0 |
| Endometriosis | 0 | 0 | 0 | 0 | 0 | 0 | 0 | 0 | 0 | 0 | 0 | 0 | 0 | 0 | 0 |
| Extensively drug-resistant tuberculosis | 0 | 0 | 0 | 0 | 0 | 0 | 0 | 0 | 0 | 0 | 0 | 0 | 0 | 0 | 0 |
| Foreign body in other body part | 0 | 0 | 0 | 0 | 0 | 0 | 0 | 0 | 0 | 0 | 0 | 0 | 1 | 0 | 1 |
| G6PD deficiency | 0 | 0 | 0 | 0 | 0 | 0 | 3 | 2 | 4 | 2 | 1 | 3 | 1 | 0 | 1 |
| Gastritis and duodenitis | 0 | 0 | 0 | 0 | 0 | 0 | 1 | 1 | 2 | 3 | 2 | 4 | 7 | 5 | 9 |
| Genital prolapse | 0 | 0 | 0 | 0 | 0 | 0 | 0 | 0 | 0 | 0 | 0 | 0 | 0 | 0 | 0 |
| Gonococcal infection | 0 | 0 | 0 | 0 | 0 | 0 | 0 | 0 | 0 | 0 | 0 | 0 | 1 | 0 | 1 |

| | | | | | | | | | | | | | | | |
|---|---|---|---|---|---|---|---|---|---|---|---|---|---|---|---|
| Hemolytic disease and other neonatal jaundice | 0 | 0 | 0 | 0 | 0 | 0 | 0 | 0 | 0 | 0 | 0 | 0 | 0 | 0 | 0 |
| HIV/AIDS - Drug-susceptible Tuberculosis | 0 | 0 | 0 | 0 | 0 | 0 | 1 | 0 | 1 | 1 | 0 | 1 | 0 | 0 | 0 |
| HIV/AIDS - Extensively drug-resistant Tuberculosis | 0 | 0 | 0 | 0 | 0 | 0 | 0 | 0 | 0 | 0 | 0 | 0 | 0 | 0 | 0 |
| HIV/AIDS - Multidrug-resistant Tuberculosis without extensive drug resistance | 0 | 0 | 0 | 0 | 0 | 0 | 0 | 0 | 0 | 0 | 0 | 0 | 0 | 0 | 0 |
| HIV/AIDS resulting in other diseases | 0 | 0 | 0 | 0 | 0 | 0 | 29 | 27 | 31 | 26 | 24 | 28 | 2 | 2 | 2 |
| Indirect maternal deaths | 0 | 0 | 0 | 0 | 0 | 0 | 1 | 1 | 2 | 0 | 0 | 0 | 0 | 0 | 0 |
| Intracerebral hemorrhage | 0 | 0 | 0 | 0 | 0 | 0 | 30 | 23 | 39 | 196 | 151 | 251 | 514 | 400 | 620 |
| Ischemic stroke | 0 | 0 | 0 | 0 | 0 | 0 | 6 | 5 | 8 | 80 | 60 | 103 | 1441 | 1113 | 1728 |
| Late maternal deaths | 0 | 0 | 0 | 0 | 0 | 0 | 2 | 1 | 3 | 0 | 0 | 0 | 0 | 0 | 0 |
| Liver cancer due to alcohol use | 0 | 0 | 0 | 0 | 0 | 0 | 5 | 3 | 7 | 67 | 46 | 93 | 47 | 35 | 64 |
| Liver cancer due to hepatitis B | 0 | 0 | 0 | 0 | 0 | 0 | 4 | 3 | 6 | 23 | 16 | 33 | 14 | 10 | 19 |
| Liver cancer due to hepatitis C | 0 | 0 | 0 | 0 | 0 | 0 | 3 | 2 | 4 | 66 | 48 | 91 | 67 | 53 | 84 |
| Liver cancer due to NASH | 0 | 0 | 0 | 0 | 0 | 0 | 1 | 1 | 2 | 14 | 10 | 20 | 21 | 16 | 28 |
| Liver cancer due to other causes | 0 | 0 | 0 | 0 | 0 | 1 | 2 | 2 | 3 | 14 | 10 | 19 | 12 | 9 | 16 |
| Maternal abortion and miscarriage | 0 | 0 | 0 | 0 | 0 | 0 | 0 | 0 | 0 | 0 | 0 | 0 | 0 | 0 | 0 |
| Maternal deaths aggravated by HIV/AIDS | 0 | 0 | 0 | 0 | 0 | 0 | 0 | 0 | 0 | 0 | 0 | 0 | 0 | 0 | 0 |
| Maternal hemorrhage | 0 | 0 | 0 | 0 | 0 | 0 | 0 | 0 | 1 | 0 | 0 | 0 | 0 | 0 | 0 |
| Maternal hypertensive disorders | 0 | 0 | 0 | 0 | 0 | 0 | 1 | 1 | 1 | 0 | 0 | 0 | 0 | 0 | 0 |
| Maternal obstructed labor and uterine rupture | 0 | 0 | 0 | 0 | 0 | 0 | 0 | 0 | 0 | 0 | 0 | 0 | 0 | 0 | 0 |
| Maternal sepsis and other maternal infections | 0 | 0 | 0 | 0 | 0 | 0 | 0 | 0 | 0 | 0 | 0 | 0 | 0 | 0 | 0 |
| Motor vehicle road injuries | 4 | 3 | 5 | 9 | 7 | 10 | 221 | 179 | 265 | 112 | 89 | 139 | 84 | 69 | 99 |
| Motorcyclist road injuries | 0 | 0 | 0 | 0 | 0 | 1 | 51 | 40 | 64 | 28 | 21 | 37 | 4 | 3 | 5 |

| | | | | | | | | | | | | | | | |
|---|---|---|---|---|---|---|---|---|---|---|---|---|---|---|---|
| Multidrug-resistant tuberculosis without extensive drug resistance | 0 | 0 | 0 | 0 | 0 | 0 | 0 | 0 | 0 | 0 | 0 | 0 | 0 | 0 | 1 |
| Myelodysplastic, myeloproliferative, and other hematopoietic neoplasms | 0 | 0 | 0 | 0 | 0 | 0 | 2 | 1 | 3 | 24 | 17 | 38 | 131 | 93 | 177 |
| Myocarditis | 1 | 1 | 1 | 0 | 0 | 1 | 6 | 4 | 9 | 4 | 2 | 7 | 5 | 2 | 8 |
| Neonatal encephalopathy due to birth asphyxia and trauma | 28 | 23 | 33 | 0 | 0 | 0 | 0 | 0 | 0 | 0 | 0 | 0 | 0 | 0 | 0 |
| Neonatal preterm birth | 75 | 64 | 85 | 0 | 0 | 0 | 0 | 0 | 0 | 0 | 0 | 0 | 0 | 0 | 0 |
| Neonatal sepsis and other neonatal infections | 9 | 7 | 11 | 0 | 0 | 0 | 0 | 0 | 0 | 0 | 0 | 0 | 0 | 0 | 0 |
| Neural tube defects | 8 | 6 | 11 | 0 | 0 | 0 | 1 | 1 | 2 | 0 | 0 | 0 | 0 | 0 | 0 |
| Non-rheumatic calcific aortic valve disease | 0 | 0 | 0 | 0 | 0 | 0 | 4 | 3 | 5 | 25 | 19 | 33 | 296 | 227 | 356 |
| Non-rheumatic degenerative mitral valve disease | 0 | 0 | 0 | 0 | 0 | 0 | 2 | 2 | 3 | 8 | 6 | 12 | 43 | 31 | 66 |
| Non-rheumatic valvular heart disease | 0 | 0 | 0 | 0 | 0 | 0 | 6 | 5 | 8 | 34 | 27 | 43 | 339 | 261 | 405 |
| Non-venomous animal contact | 0 | 0 | 0 | 0 | 0 | 0 | 1 | 1 | 1 | 1 | 1 | 2 | 1 | 1 | 1 |
| Opioid use disorders | 0 | 0 | 0 | 0 | 0 | 0 | 542 | 441 | 659 | 174 | 127 | 234 | 22 | 17 | 28 |
| Orofacial clefts | 0 | 0 | 0 | 0 | 0 | 0 | 0 | 0 | 0 | 0 | 0 | 0 | 0 | 0 | 0 |
| Other cardiomyopathy | 1 | 1 | 2 | 1 | 0 | 1 | 24 | 17 | 36 | 71 | 50 | 119 | 200 | 154 | 272 |
| Other chromosomal abnormalities | 16 | 13 | 22 | 1 | 1 | 1 | 2 | 1 | 3 | 1 | 1 | 1 | 0 | 0 | 0 |
| Other congenital birth defects | 18 | 15 | 23 | 2 | 2 | 3 | 5 | 4 | 7 | 2 | 1 | 3 | 0 | 0 | 0 |
| Other drug use disorders | 0 | 0 | 0 | 0 | 0 | 0 | 57 | 45 | 72 | 18 | 13 | 25 | 6 | 4 | 7 |
| Other exposure to mechanical forces | 1 | 1 | 1 | 1 | 1 | 1 | 12 | 9 | 15 | 12 | 9 | 16 | 9 | 7 | 11 |
| Other gynecological diseases | 0 | 0 | 0 | 0 | 0 | 0 | 0 | 0 | 0 | 0 | 0 | 1 | 1 | 1 | 1 |
| Other hemoglobinopathies and hemolytic anemias | 0 | 0 | 0 | 0 | 0 | 0 | 1 | 1 | 2 | 4 | 3 | 5 | 15 | 11 | 18 |
| Other leukemia | 0 | 0 | 0 | 1 | 0 | 1 | 6 | 4 | 7 | 23 | 17 | 30 | 75 | 57 | 97 |

| | | | | | | | | | | | | | | | |
|---|---|---|---|---|---|---|---|---|---|---|---|---|---|---|---|
| Other malignant neoplasms | 3 | 2 | 3 | 5 | 4 | 6 | 40 | 32 | 50 | 106 | 85 | 131 | 158 | 127 | 189 |
| Other maternal disorders | 0 | 0 | 0 | 0 | 0 | 0 | 3 | 2 | 5 | 0 | 0 | 0 | 0 | 0 | 0 |
| Other neonatal disorders | 30 | 23 | 38 | 0 | 0 | 0 | 0 | 0 | 0 | 0 | 0 | 0 | 0 | 0 | 0 |
| Other non-rheumatic valve diseases | 0 | 0 | 0 | 0 | 0 | 0 | 0 | 0 | 0 | 0 | 0 | 0 | 0 | 0 | 1 |
| Other pneumoconiosis | 0 | 0 | 0 | 0 | 0 | 0 | 0 | 0 | 0 | 0 | 0 | 1 | 1 | 0 | 1 |
| Other road injuries | 0 | 0 | 0 | 0 | 0 | 0 | 2 | 1 | 2 | 1 | 1 | 2 | 1 | 1 | 1 |
| Other sexually transmitted infections | 0 | 0 | 0 | 0 | 0 | 0 | 0 | 0 | 0 | 0 | 0 | 0 | 0 | 0 | 0 |
| Other urinary diseases | 0 | 0 | 0 | 0 | 0 | 0 | 1 | 0 | 1 | 3 | 2 | 5 | 9 | 6 | 13 |
| Paratyphoid fever | 0 | 0 | 0 | 0 | 0 | 0 | 0 | 0 | 0 | 0 | 0 | 0 | 0 | 0 | 0 |
| Pedestrian road injuries | 2 | 2 | 3 | 3 | 3 | 4 | 39 | 31 | 47 | 33 | 25 | 42 | 23 | 18 | 27 |
| Peptic ulcer disease | 0 | 0 | 0 | 0 | 0 | 0 | 4 | 3 | 5 | 21 | 14 | 29 | 61 | 46 | 78 |
| Physical violence by firearm | 1 | 1 | 1 | 2 | 2 | 3 | 98 | 79 | 120 | 18 | 14 | 23 | 4 | 3 | 5 |
| Physical violence by other means | 6 | 5 | 7 | 2 | 1 | 2 | 18 | 15 | 22 | 10 | 8 | 12 | 4 | 3 | 5 |
| Physical violence by sharp object | 0 | 0 | 0 | 0 | 0 | 0 | 18 | 14 | 22 | 6 | 5 | 8 | 2 | 1 | 2 |
| Poisoning by carbon monoxide | 0 | 0 | 1 | 1 | 0 | 1 | 14 | 10 | 17 | 7 | 5 | 9 | 3 | 3 | 4 |
| Poisoning by other means | 1 | 0 | 1 | 1 | 0 | 1 | 4 | 3 | 5 | 3 | 2 | 4 | 2 | 1 | 2 |
| Pulmonary aspiration and foreign body in airway | 15 | 11 | 18 | 2 | 1 | 2 | 15 | 12 | 18 | 29 | 23 | 36 | 88 | 70 | 105 |
| Pyoderma | 0 | 0 | 0 | 0 | 0 | 0 | 1 | 1 | 3 | 8 | 4 | 14 | 25 | 13 | 48 |
| Self-harm by firearm | 0 | 0 | 0 | 4 | 2 | 6 | 269 | 212 | 351 | 204 | 153 | 276 | 102 | 80 | 130 |
| Self-harm by other specified means | 0 | 0 | 0 | 7 | 4 | 9 | 304 | 220 | 372 | 167 | 115 | 213 | 41 | 31 | 51 |
| Sickle cell disorders | 0 | 0 | 0 | 0 | 0 | 0 | 1 | 1 | 2 | 1 | 0 | 1 | 0 | 0 | 0 |
| Silicosis | 0 | 0 | 0 | 0 | 0 | 0 | 0 | 0 | 0 | 1 | 1 | 2 | 3 | 2 | 5 |
| Subarachnoid hemorrhage | 0 | 0 | 1 | 0 | 0 | 1 | 27 | 21 | 35 | 93 | 72 | 120 | 151 | 116 | 183 |
| Syphilis | 0 | 0 | 0 | 0 | 0 | 0 | 0 | 0 | 0 | 0 | 0 | 0 | 0 | 0 | 1 |
| Thalassemias | 0 | 0 | 0 | 0 | 0 | 0 | 1 | 0 | 1 | 0 | 0 | 0 | 0 | 0 | 0 |
| Typhoid fever | 0 | 0 | 0 | 0 | 0 | 0 | 0 | 0 | 0 | 0 | 0 | 0 | 0 | 0 | 0 |
| Unintentional firearm injuries | 0 | 0 | 0 | 1 | 0 | 1 | 5 | 4 | 7 | 2 | 1 | 3 | 1 | 1 | 1 |
| Urinary tract infections and interstitial nephritis | 0 | 0 | 0 | 0 | 0 | 0 | 5 | 4 | 7 | 35 | 27 | 43 | 247 | 195 | 306 |

| | | | | | | | | | | | | | | | |
|---|---|---|---|---|---|---|---|---|---|---|---|---|---|---|---|
| Urogenital congenital anomalies | 3 | 2 | 5 | 0 | 0 | 0 | 0 | 0 | 0 | 0 | 0 | 0 | 0 | 0 | 0 |
| Urolithiasis | 0 | 0 | 0 | 0 | 0 | 0 | 0 | 0 | 1 | 2 | 1 | 2 | 6 | 4 | 8 |
| Uterine fibroids | 0 | 0 | 0 | 0 | 0 | 0 | 0 | 0 | 0 | 0 | 0 | 0 | 0 | 0 | 0 |
| Venomous animal contact | 0 | 0 | 0 | 0 | 0 | 0 | 0 | 0 | 0 | 0 | 0 | 1 | 0 | 0 | 0 |
| Visceral leishmaniasis | 0 | 0 | 0 | 0 | 0 | 0 | 0 | 0 | 0 | 0 | 0 | 0 | 0 | 0 | 0 |
| **Communicable, maternal, neonatal, and nutritional diseases** | 156 | 140 | 173 | 4 | 4 | 5 | 102 | 88 | 118 | 259 | 210 | 317 | 1,080 | 863 | 1,279 |
| **Injuries** | 39 | 34 | 44 | 43 | 39 | 47 | 1,218 | 1,006 | 1,453 | 856 | 686 | 1,055 | 1,292 | 1,058 | 1,503 |
| **Non-communicable diseases** | 120 | 107 | 136 | 40 | 37 | 43 | 2,276 | 1,908 | 2,701 | 9,240 | 7,641 | 11,105 | 23,998 | 21,023 | 27,055 |
| **All causes** | **315** | **288** | **343** | **88** | **83** | **93** | **3,595** | **3,022** | **4,247** | **10,356** | **8,569** | **12,455** | **26,370** | **23,129** | **29,697** |

Table S2. All-cause mortality estimates for Colorado and the United States, in 1990, 2000, 2010, 2019

| | Number of deaths | Mortality, per 100,000 people population | Age-standardized mortality, per 100,000 people |
|---|---|---|---|
| **Colorado** | | | |
| Year | | | |
| 1990 | 21171<br>(20748 - 21603) | 629.47<br>(642.3 - 616.88) | 596.92<br>(608.99 - 585.08) |
| 2000 | 26898<br>(26408 - 27388) | 628.36<br>(639.8 - 616.9) | 567.62<br>(577.87 - 557.17) |
| 2010 | 30905<br>(30371 - 31480) | 614.83<br>(626.27 - 604.21) | 489.76<br>(498.6 - 481.4) |
| 2019 | 40724<br>(35134 - 46743) | 754<br>(865.45 - 650.5) | 494.26<br>(567.25 - 426.48) |
| **United States** | | | |
| Year | | | |
| 1990 | 2132451<br>(2128722 - 2136181) | 840.85<br>(842.32 - 839.38) | 668.88<br>(670.02 - 667.7) |
| 2000 | 2388367<br>(2383994- 2392982) | 850.92<br>(852.56 - 849.36) | 618.62<br>(619.85 - 617.48) |
| 2010 | 2474654<br>(2470219 - 2479225) | 800.95<br>(802.43 - 799.52) | 531.79<br>(532.83 - 530.83) |
| 2019 | 2946456<br>(2924325 - 2968832) | 898.37<br>(905.19 - 891.62) | 528.24<br>(532.56 - 524.04) |

Data are for all ages, unless otherwise specified, and data in parentheses are 95% uncertainty intervals

**Figure S1.** Arrow diagram of top ranked causes of death, in Colorado, in (A) both sexes (B) females and (C) males, Level 4, age standardized, 1990 - 2019

A. Both Sexes

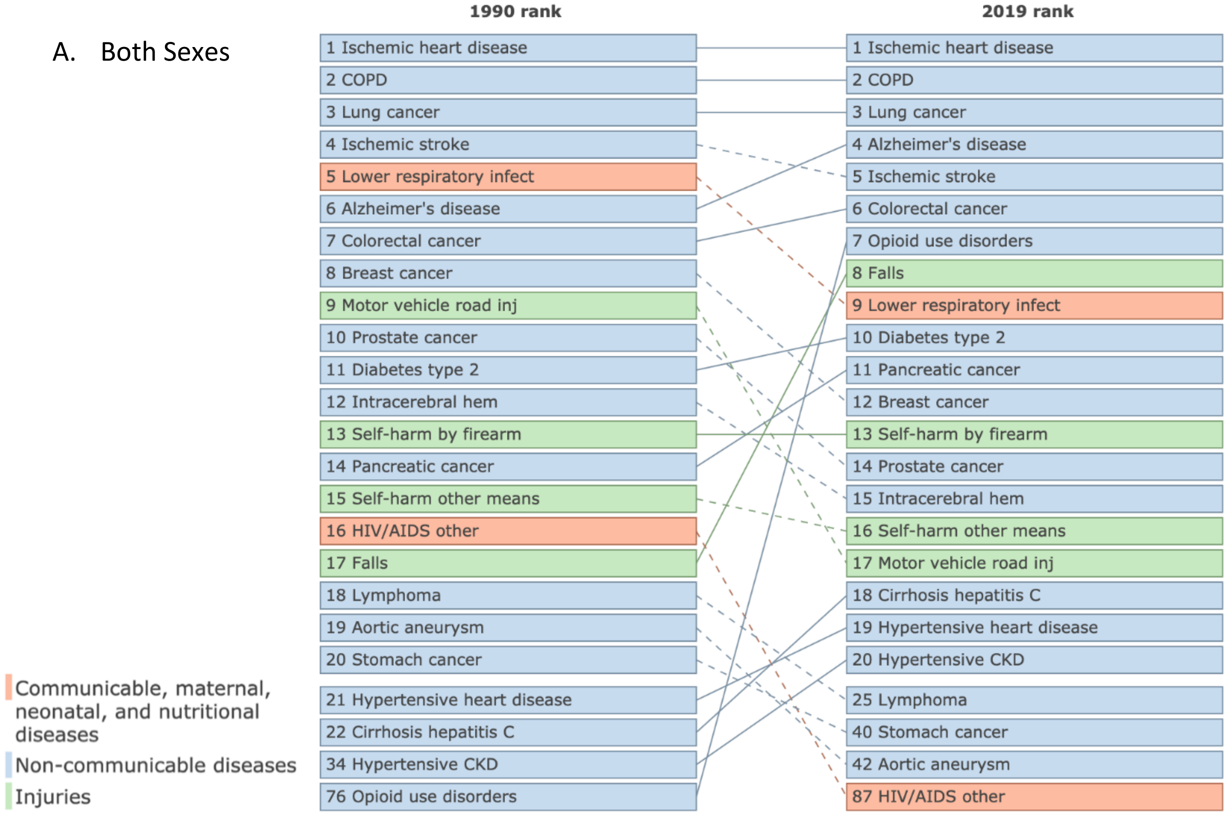


B. Females

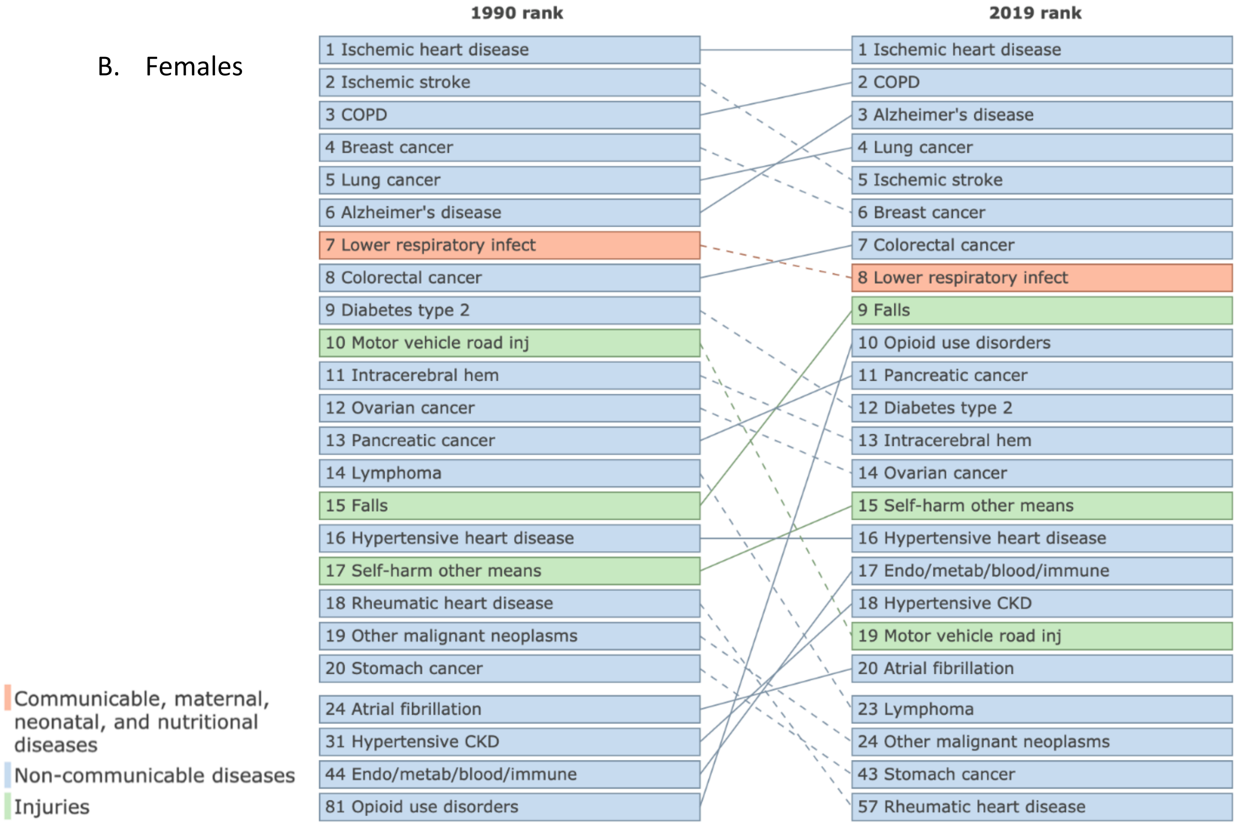

C. Males

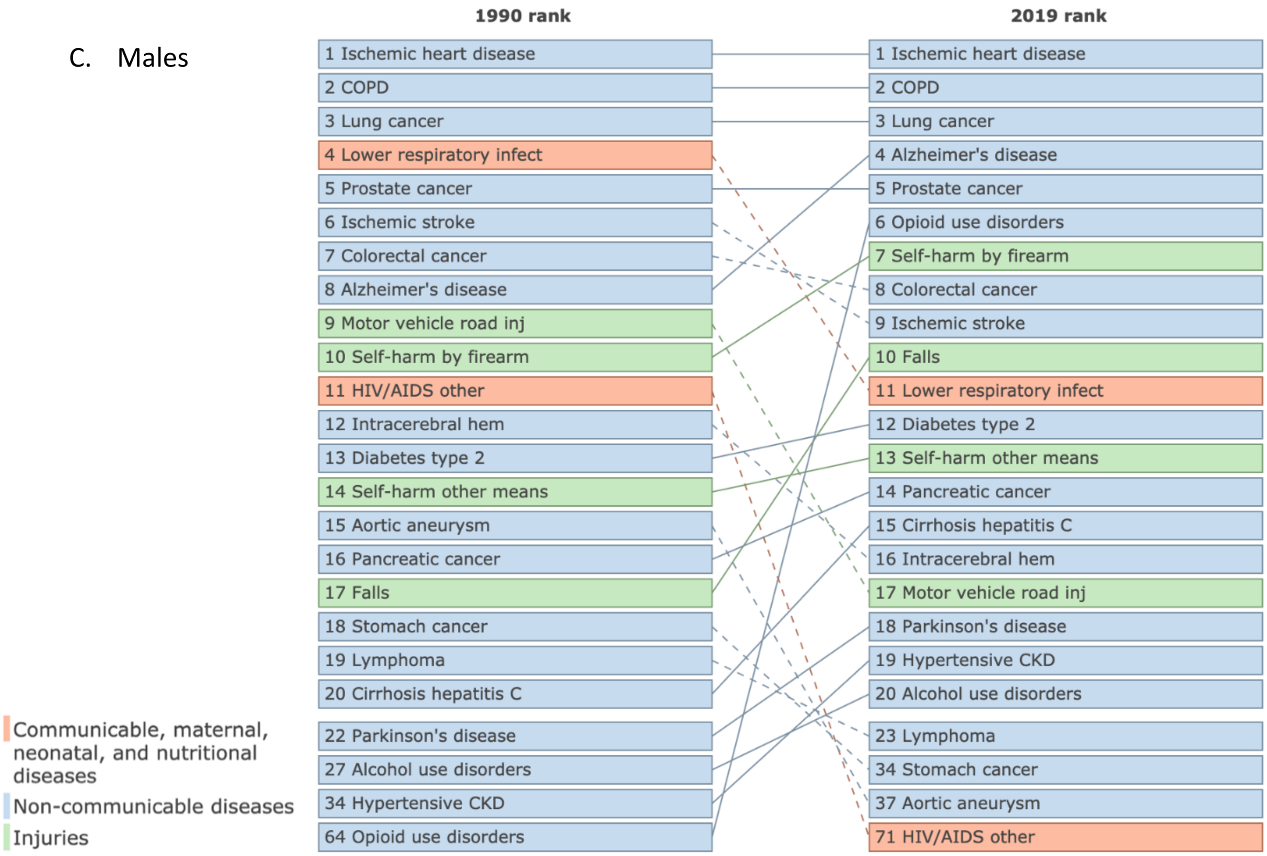
1990 rank
2019 rank
1 Ischemic heart disease
2 COPD
3 Lung cancer
4 Lower respiratory infect
5 Prostate cancer
6 Ischemic stroke
7 Colorectal cancer
8 Alzheimer's disease
9 Motor vehicle road inj
10 Self-harm by firearm
11 HIV/AIDS other
12 Intracerebral hem
13 Diabetes type 2
14 Self-harm other means
15 Aortic aneurysm
16 Pancreatic cancer
17 Falls
18 Stomach cancer
19 Lymphoma
20 Cirrhosis hepatitis C
22 Parkinson's disease
27 Alcohol use disorders
34 Hypertensive CKD
64 Opioid use disorders
1 Ischemic heart disease
2 COPD
3 Lung cancer
4 Alzheimer's disease
5 Prostate cancer
6 Opioid use disorders
7 Self-harm by firearm
8 Colorectal cancer
9 Ischemic stroke
10 Falls
11 Lower respiratory infect
12 Diabetes type 2
13 Self-harm other means
14 Pancreatic cancer
15 Cirrhosis hepatitis C
16 Intracerebral hem
17 Motor vehicle road inj
18 Parkinson's disease
19 Hypertensive CKD
20 Alcohol use disorders
23 Lymphoma
34 Stomach cancer
37 Aortic aneurysm
71 HIV/AIDS other
Communicable, maternal, neonatal, and nutritional diseases
Non-communicable diseases
Injuries

**Figure S2.** Heat map of top causes of death in Colorado, West States, and the United States, for both sexes, Level 4, age-standardized, in 1990. *Numbers are rankings, with colors indicating the scale from low (blue: below 17th) to high (red: first and second) rankings.

| | **Colorado** | United States of America | Arizona | California | Idaho | Montana | Nevada | New Mexico | Oregon | Utah | Washington | Wyoming |
|---|---|---|---|---|---|---|---|---|---|---|---|---|
| Ischemic heart disease | 1 | 1 | 1 | 1 | 1 | 1 | 1 | 1 | 1 | 1 | 1 | 1 |
| COPD | 2 | 4 | 3 | 4 | 4 | 3 | 3 | 3 | 4 | 3 | 4 | 3 |
| Lung cancer | 3 | 2 | 2 | 2 | 2 | 2 | 2 | 2 | 2 | 5 | 2 | 2 |
| Ischemic stroke | 4 | 3 | 4 | 3 | 3 | 4 | 4 | 4 | 3 | 2 | 3 | 4 |
| Lower respiratory infect | 5 | 5 | 5 | 5 | 5 | 6 | 5 | 6 | 6 | 4 | 5 | 5 |
| Alzheimer's disease | 6 | 6 | 6 | 6 | 6 | 5 | 7 | 5 | 5 | 6 | 6 | 6 |
| Colorectal cancer | 7 | 7 | 7 | 7 | 8 | 8 | 6 | 9 | 7 | 8 | 7 | 8 |
| Breast cancer | 8 | 8 | 9 | 9 | 9 | 9 | 9 | 10 | 9 | 9 | 8 | 9 |
| Motor vehicle road inj | 9 | 10 | 8 | 10 | 7 | 7 | 11 | 7 | 8 | 10 | 9 | 7 |
| Prostate cancer | 10 | 12 | 12 | 13 | 11 | 11 | 13 | 12 | 11 | 11 | 12 | 12 |
| Diabetes type 2 | 11 | 9 | 10 | 12 | 10 | 10 | 14 | 8 | 10 | 7 | 10 | 10 |
| Intracerebral hem | 12 | 11 | 13 | 11 | 13 | 13 | 12 | 13 | 12 | 12 | 11 | 13 |
| Self-harm by firearm | 13 | 16 | 11 | 18 | 12 | 12 | 10 | 11 | 13 | 13 | 14 | 11 |
| Pancreatic cancer | 14 | 14 | 14 | 14 | 14 | 14 | 15 | 14 | 14 | 15 | 13 | 14 |
| Self-harm other means | 15 | 22 | 17 | 22 | 19 | 16 | 17 | 16 | 16 | 14 | 15 | 19 |
| HIV/AIDS other | 16 | 13 | 16 | 8 | 63 | 70 | 21 | 28 | 21 | 44 | 17 | 76 |
| Falls | 17 | 25 | 21 | 34 | 18 | 17 | 27 | 17 | 20 | 17 | 23 | 17 |
| Lymphoma | 18 | 17 | 15 | 19 | 15 | 15 | 18 | 22 | 15 | 18 | 16 | 15 |
| Aortic aneurysm | 19 | 23 | 26 | 24 | 16 | 18 | 23 | 27 | 18 | 23 | 21 | 16 |
| Stomach cancer | 20 | 21 | 19 | 20 | 17 | 19 | 22 | 19 | 19 | 19 | 20 | 18 |
| Hypertensive heart disease | 21 | 15 | 25 | 15 | 20 | 22 | 8 | 20 | 22 | 16 | 18 | 21 |
| Cirrhosis hepatitis C | 22 | 24 | 18 | 21 | 29 | 24 | 19 | 15 | 28 | 28 | 27 | 20 |
| Other cardiomyopathy | 23 | 19 | 20 | 17 | 22 | 20 | 16 | 25 | 25 | 20 | 22 | 22 |
| Ovarian cancer | 24 | 26 | 28 | 25 | 23 | 23 | 33 | 32 | 23 | 25 | 24 | 24 |
| Neonatal preterm birth | 25 | 20 | 22 | 23 | 25 | 30 | 41 | 24 | 34 | 42 | 33 | 28 |

**Figure S3.** Heat map of top causes of death in Colorado, West States, and the United States, for (A) females and (B) males, Level 4, age-standardized, in 2019. *Numbers are rankings, with colors indicating the scale from low (blue: below 17th) to high (red: first and second) rankings.

A. Females

| | Colorado | United States of America | Arizona | California | Idaho | Montana | Nevada | New Mexico | Oregon | Utah | Washington | Wyoming |
|---|---|---|---|---|---|---|---|---|---|---|---|---|
| Ischemic heart disease | 1 | 1 | 1 | 1 | 1 | 1 | 1 | 1 | 1 | 1 | 1 | 1 |
| COPD | 2 | 3 | 2 | 3 | 2 | 2 | 2 | 2 | 3 | 2 | 3 | 2 |
| Alzheimer's disease | 3 | 4 | 4 | 4 | 4 | 4 | 5 | 3 | 4 | 5 | 4 | 4 |
| Lung cancer | 4 | 2 | 3 | 2 | 3 | 3 | 3 | 4 | 2 | 6 | 2 | 3 |
| Ischemic stroke | 5 | 6 | 6 | 6 | 5 | 5 | 4 | 6 | 5 | 3 | 5 | 6 |
| Breast cancer | 6 | 5 | 5 | 5 | 6 | 6 | 6 | 5 | 6 | 4 | 6 | 5 |
| Colorectal cancer | 7 | 7 | 7 | 7 | 8 | 7 | 8 | 8 | 7 | 9 | 7 | 8 |
| Lower respiratory infect | 8 | 8 | 9 | 8 | 7 | 8 | 7 | 10 | 11 | 7 | 9 | 7 |
| Falls | 9 | 16 | 13 | 25 | 12 | 12 | 17 | 11 | 12 | 12 | 13 | 14 |
| Opioid use disorders | 10 | 12 | 10 | 14 | 14 | 14 | 11 | 9 | 15 | 13 | 11 | 10 |
| Pancreatic cancer | 11 | 11 | 11 | 10 | 11 | 11 | 12 | 12 | 9 | 11 | 10 | 12 |
| Diabetes type 2 | 12 | 9 | 8 | 9 | 9 | 9 | 13 | 7 | 8 | 8 | 8 | 9 |
| Intracerebral hem | 13 | 10 | 14 | 11 | 10 | 13 | 10 | 14 | 10 | 10 | 12 | 13 |
| Ovarian cancer | 14 | 14 | 15 | 13 | 13 | 15 | 14 | 16 | 13 | 15 | 14 | 15 |
| Self-harm other means | 15 | 25 | 19 | 23 | 21 | 16 | 16 | 19 | 20 | 14 | 20 | 23 |
| Hypertensive heart disease | 16 | 13 | 12 | 12 | 16 | 18 | 9 | 18 | 18 | 16 | 15 | 18 |
| Endo/metab/blood/immune | 17 | 15 | 16 | 16 | 17 | 17 | 22 | 17 | 16 | 17 | 16 | 16 |
| Hypertensive CKD | 18 | 17 | 20 | 15 | 20 | 20 | 15 | 20 | 19 | 18 | 19 | 17 |
| Motor vehicle road inj | 19 | 18 | 17 | 21 | 15 | 10 | 21 | 13 | 21 | 22 | 25 | 11 |
| Atrial fibrillation | 20 | 20 | 21 | 17 | 19 | 19 | 19 | 26 | 17 | 19 | 18 | 19 |
| Cirrhosis hepatitis C | 21 | 27 | 18 | 19 | 26 | 22 | 18 | 15 | 23 | 29 | 23 | 20 |
| Other cardiovascular | 22 | 28 | 28 | 34 | 28 | 31 | 30 | 30 | 30 | 25 | 29 | 22 |
| Lymphoma | 23 | 21 | 23 | 22 | 22 | 23 | 25 | 25 | 22 | 23 | 21 | 21 |
| Other malignant neoplasms | 24 | 22 | 24 | 20 | 27 | 24 | 28 | 24 | 24 | 26 | 22 | 26 |
| Parkinson's disease | 25 | 30 | 25 | 27 | 25 | 27 | 29 | 27 | 27 | 27 | 27 | 28 |

B. Males

| | Colorado | United States of America | Arizona | California | Idaho | Montana | Nevada | New Mexico | Oregon | Utah | Washington | Wyoming |
|---|---|---|---|---|---|---|---|---|---|---|---|---|
| Ischemic heart disease | 1 | 1 | 1 | 1 | 1 | 1 | 1 | 1 | 1 | 1 | 1 | 1 |
| COPD | 2 | 3 | 2 | 3 | 2 | 2 | 2 | 2 | 3 | 2 | 3 | 2 |
| Lung cancer | 3 | 2 | 3 | 2 | 3 | 3 | 3 | 3 | 2 | 3 | 2 | 3 |
| Alzheimer's disease | 4 | 6 | 6 | 5 | 6 | 5 | 9 | 6 | 5 | 11 | 6 | 8 |
| Prostate cancer | 5 | 5 | 5 | 4 | 4 | 4 | 4 | 7 | 4 | 4 | 4 | 6 |
| Opioid use disorders | 6 | 4 | 4 | 10 | 11 | 10 | 6 | 4 | 7 | 5 | 5 | 5 |
| Self-harm by firearm | 7 | 14 | 9 | 20 | 7 | 6 | 11 | 10 | 10 | 9 | 13 | 4 |
| Colorectal cancer | 8 | 7 | 8 | 6 | 8 | 8 | 7 | 8 | 9 | 10 | 9 | 10 |
| Ischemic stroke | 9 | 9 | 10 | 9 | 5 | 9 | 10 | 13 | 6 | 7 | 7 | 11 |
| Falls | 10 | 16 | 13 | 22 | 15 | 13 | 18 | 15 | 13 | 13 | 10 | 13 |
| Lower respiratory infect | 11 | 8 | 11 | 7 | 10 | 12 | 5 | 11 | 14 | 8 | 11 | 9 |
| Diabetes type 2 | 12 | 10 | 7 | 8 | 9 | 11 | 14 | 5 | 8 | 6 | 8 | 12 |
| Self-harm other means | 13 | 19 | 18 | 15 | 17 | 15 | 16 | 17 | 16 | 12 | 15 | 15 |
| Pancreatic cancer | 14 | 12 | 12 | 11 | 13 | 14 | 13 | 16 | 11 | 15 | 12 | 14 |
| Cirrhosis hepatitis C | 15 | 18 | 14 | 14 | 18 | 17 | 15 | 9 | 15 | 20 | 17 | 16 |
| Intracerebral hem | 16 | 11 | 17 | 12 | 14 | 16 | 12 | 18 | 12 | 14 | 14 | 17 |
| Motor vehicle road inj | 17 | 15 | 15 | 18 | 12 | 7 | 20 | 14 | 17 | 19 | 21 | 7 |
| Parkinson's disease | 18 | 20 | 19 | 16 | 16 | 18 | 19 | 20 | 19 | 16 | 16 | 19 |
| Hypertensive CKD | 19 | 17 | 22 | 17 | 19 | 20 | 17 | 23 | 22 | 17 | 22 | 20 |
| Alcohol use disorders | 20 | 32 | 20 | 33 | 31 | 19 | 23 | 12 | 21 | 25 | 33 | 18 |
| Hypertensive heart disease | 21 | 13 | 16 | 13 | 25 | 26 | 8 | 22 | 31 | 22 | 19 | 23 |
| Esophageal cancer | 22 | 22 | 24 | 27 | 23 | 23 | 22 | 25 | 23 | 29 | 20 | 21 |
| Lymphoma | 23 | 24 | 27 | 24 | 21 | 24 | 25 | 27 | 24 | 21 | 24 | 26 |
| Atrial fibrillation | 24 | 28 | 28 | 25 | 22 | 25 | 28 | 31 | 26 | 23 | 26 | 25 |
| Endo/metab/blood/immune | 25 | 21 | 21 | 21 | 20 | 21 | 30 | 21 | 18 | 18 | 18 | 22 |

Table S3. Colorado disability adjusted life years (DALYs), by age group and in all ages, in 2019

| | DALYs 2019 | |
|---|---|---|
| | Count | Age-standardized rate (per 100,000 people) |
| 0 – 9 years | 48,694 (42,940 – 55,550) | 3,804 (3,354.5 – 4,340.5) |
| 10 – 24 years | 141,830 (11,4279 – 17,2473) | 13,127 (10,577.3 – 15,964.5) |
| 25 – 49 years | 424,962 (350,912 – 511,536) | 23,281 (19,224.6 – 28,024.3) |
| 50 – 74 years | 725,882 (608,019 – 845,493) | 25,150 (21,066.4 – 29,294.3) |
| ≥ 75 years | 335,123 (297,650 – 374,010) | 68,854 (61,154.3 – 7,6843.3) |
| All ages | 1,676,491 (1,427,654 – 1,953,173) | 31,040 (26,432.8 – 36,162.7) |

DALY = disability-adjusted life-year

**Table S4.** Main causes of disability-adjusted life-years (DALYs) for Colorado in, females and males, all ages, in 2019

| Cause | Disability adjusted life years (DALYs) | | | | | |
|---|---|---|---|---|---|---|
| | Females | | | Males | | |
| | Central Value | Lower Uncertainty Level | Upper Uncertainty Level | Central Value | Lower Uncertainty Level | Upper Uncertainty Level |
| Acute hepatitis A | 68 | 45 | 100 | 76 | 50 | 109 |
| Acute hepatitis B | 10 | 7 | 15 | 19 | 13 | 28 |
| Acute hepatitis C | 5 | 3 | 8 | 9 | 5 | 15 |
| Acute hepatitis E | 6 | 4 | 9 | 7 | 4 | 10 |
| Acute lymphoid leukemia | 415 | 320 | 545 | 661 | 482 | 849 |
| Acute myeloid leukemia | 1,847 | 1,407 | 2,385 | 2,670 | 1,950 | 3,498 |
| Alcoholic cardiomyopathy | 239 | 172 | 382 | 1,692 | 1,143 | 2,462 |
| Amphetamine use disorders | 1,367 | 969 | 1,813 | 2,560 | 1,969 | 3,251 |
| Anorexia nervosa | 1,072 | 644 | 1,661 | 246 | 137 | 395 |
| Asbestosis | 10 | 6 | 14 | 169 | 122 | 228 |
| Ascariasis | 0 | 0 | 0 | 0 | 0 | 0 |
| Bulimia nervosa | 2,383 | 1,383 | 3,776 | 1,001 | 567 | 1,596 |
| Cellulitis | 599 | 333 | 810 | 629 | 296 | 1,033 |
| Chlamydial infection | 28 | 19 | 41 | 5 | 3 | 9 |
| Chronic kidney disease | 12,595 | 10,226 | 15,179 | 14,622 | 11,935 | 17,720 |
| Chronic kidney disease due to diabetes mellitus type 1 | 391 | 252 | 579 | 531 | 330 | 815 |
| Chronic kidney disease due to diabetes mellitus type 2 | 3,557 | 2,691 | 4,604 | 3,717 | 2,678 | 4,991 |
| Chronic kidney disease due to glomerulonephritis | 1,595 | 1,191 | 2,088 | 2,022 | 1,474 | 2,719 |
| Chronic kidney disease due to hypertension | 3,920 | 3,017 | 4,962 | 5,034 | 3,841 | 6,404 |
| Chronic kidney disease due to other and unspecified causes | 3,130 | 2,368 | 3,968 | 3,319 | 2,476 | 4,215 |
| Chronic lymphoid leukemia | 491 | 360 | 648 | 1,127 | 811 | 1,553 |
| Chronic myeloid leukemia | 204 | 151 | 274 | 336 | 239 | 456 |
| Cirrhosis and other chronic liver diseases due to alcohol use | 2,522 | 1,787 | 3,438 | 6,256 | 4,467 | 8,489 |
| Cirrhosis and other chronic liver diseases due to hepatitis B | 257 | 184 | 349 | 622 | 448 | 841 |
| Cirrhosis and other chronic liver diseases due to hepatitis C | 4,761 | 3,552 | 6,289 | 11,093 | 8,230 | 14,853 |
| Cirrhosis and other chronic liver diseases due to NAFLD | 1,488 | 933 | 2,245 | 1,341 | 823 | 2,065 |
| Cirrhosis and other chronic liver diseases due to other causes | 2,723 | 1,829 | 3,906 | 2,355 | 1,504 | 3,499 |
| Coal workers pneumoconiosis | 3 | 2 | 5 | 63 | 43 | 91 |
| Cocaine use disorders | 3,048 | 2,246 | 3,972 | 6,144 | 4,823 | 7,804 |
| Congenital heart anomalies | 1,290 | 1,011 | 1,814 | 1,751 | 1,339 | 2,219 |
| Congenital musculoskeletal and limb anomalies | 1,159 | 795 | 1,595 | 930 | 636 | 1,281 |
| Cyclist road injuries | 511 | 392 | 650 | 1,574 | 1,256 | 1,944 |
| Diabetes mellitus type 1 | 2,100 | 1,531 | 2,786 | 3,046 | 2,233 | 4,035 |

| | | | | | | |
|---|---|---|---|---|---|---|
| Diabetes mellitus type 2 | 20,503 | 15,612 | 26,109 | 27,358 | 21,089 | 34,658 |
| Digestive congenital anomalies | 277 | 192 | 432 | 366 | 245 | 487 |
| Down syndrome | 326 | 244 | 418 | 477 | 363 | 622 |
| Drug-susceptible tuberculosis | 127 | 97 | 159 | 172 | 129 | 219 |
| Ectopic pregnancy | 15 | 10 | 21 | 0 | 0 | 0 |
| Endometriosis | 903 | 550 | 1,414 | 0 | 0 | 0 |
| Extensively drug-resistant tuberculosis | 1 | 0 | 4 | 1 | 0 | 5 |
| Foreign body in other body part | 455 | 329 | 605 | 470 | 338 | 638 |
| G6PD deficiency | 92 | 62 | 135 | 172 | 126 | 226 |
| Gastritis and duodenitis | 485 | 338 | 674 | 400 | 289 | 533 |
| Genital prolapse | 318 | 155 | 592 | 0 | 0 | 0 |
| Gonococcal infection | 32 | 25 | 40 | 5 | 4 | 8 |
| Hemolytic disease and other neonatal jaundice | 53 | 40 | 68 | 57 | 44 | 73 |
| HIV/AIDS - Drug-susceptible Tuberculosis | 10 | 6 | 15 | 42 | 26 | 66 |
| HIV/AIDS - Extensively drug-resistant Tuberculosis | 0 | 0 | 0 | 0 | 0 | 2 |
| HIV/AIDS - Multidrug-resistant Tuberculosis without extensive drug resistance | 0 | 0 | 1 | 2 | 0 | 6 |
| HIV/AIDS resulting in other diseases | 641 | 515 | 799 | 2,809 | 2,261 | 3,550 |
| Indirect maternal deaths | 86 | 58 | 120 | 0 | 0 | 0 |
| Intracerebral hemorrhage | 6,707 | 5,402 | 8,354 | 7,682 | 5,797 | 9,945 |
| Ischemic stroke | 16,370 | 13,447 | 19,280 | 11,254 | 9,088 | 13,460 |
| Late maternal deaths | 115 | 65 | 187 | 0 | 0 | 0 |
| Liver cancer due to alcohol use | 328 | 236 | 446 | 2,538 | 1,775 | 3,470 |
| Liver cancer due to hepatitis B | 247 | 182 | 331 | 859 | 588 | 1,222 |
| Liver cancer due to hepatitis C | 1,138 | 858 | 1,478 | 1,819 | 1,257 | 2,560 |
| Liver cancer due to NASH | 290 | 215 | 389 | 467 | 319 | 662 |
| Liver cancer due to other causes | 301 | 229 | 387 | 457 | 317 | 630 |
| Maternal abortion and miscarriage | 15 | 10 | 21 | 0 | 0 | 0 |
| Maternal deaths aggravated by HIV/AIDS | 0 | 0 | 1 | 0 | 0 | 0 |
| Maternal hemorrhage | 108 | 68 | 168 | 0 | 0 | 0 |
| Maternal hypertensive disorders | 149 | 102 | 211 | 0 | 0 | 0 |
| Maternal obstructed labor and uterine rupture | 34 | 15 | 59 | 0 | 0 | 0 |
| Maternal sepsis and other maternal infections | 63 | 32 | 112 | 0 | 0 | 0 |
| Motor vehicle road injuries | 8,991 | 7,325 | 10,895 | 15,458 | 12,365 | 18,881 |
| Motorcyclist road injuries | 829 | 657 | 1,006 | 4,872 | 3,962 | 5,930 |
| Multidrug-resistant tuberculosis without extensive drug resistance | 4 | 0 | 15 | 5 | 0 | 19 |
| Myelodysplastic, myeloproliferative, and other hematopoietic neoplasms | 1,080 | 845 | 1,361 | 1,618 | 1,111 | 2,557 |
| Myocarditis | 321 | 202 | 442 | 408 | 242 | 561 |
| Neonatal encephalopathy due to birth asphyxia and trauma | 1,890 | 1,569 | 2,244 | 2,130 | 1,701 | 2,654 |
| Neonatal preterm birth | 6,701 | 5,689 | 7,861 | 7,520 | 6,385 | 8,876 |
| Neonatal sepsis and other neonatal infections | 479 | 381 | 582 | 574 | 453 | 703 |
| Neural tube defects | 463 | 332 | 620 | 392 | 267 | 536 |
| Non-rheumatic calcific aortic valve disease | 1,914 | 1,484 | 2,410 | 1,981 | 1,553 | 2,507 |

| | | | | | | |
|---|---|---|---|---|---|---|
| Non-rheumatic degenerative mitral valve disease | 730 | 541 | 1,058 | 548 | 402 | 767 |
| Non-rheumatic valvular heart disease | 2,657 | 2,111 | 3,305 | 2,540 | 2,020 | 3,199 |
| Non-venomous animal contact | 294 | 185 | 463 | 322 | 215 | 496 |
| Opioid use disorders | 28,514 | 21,487 | 36,370 | 43,372 | 34,325 | 53,513 |
| Orofacial clefts | 30 | 19 | 44 | 32 | 20 | 48 |
| Other cardiomyopathy | 2,648 | 2,049 | 3,639 | 3,802 | 2,739 | 6,185 |
| Other chromosomal abnormalities | 1,004 | 758 | 1,439 | 999 | 756 | 1,326 |
| Other congenital birth defects | 1,095 | 822 | 1,477 | 1,417 | 1,090 | 1,855 |
| Other drug use disorders | 2,994 | 2,206 | 3,898 | 4,033 | 3,083 | 5,191 |
| Other exposure to mechanical forces | 2,734 | 1,857 | 3,947 | 5,704 | 3,974 | 8,183 |
| Other gynecological diseases | 6,416 | 4,298 | 8,944 | 0 | 0 | 0 |
| Other hemoglobinopathies and hemolytic anemias | 375 | 273 | 510 | 217 | 164 | 289 |
| Other leukemia | 757 | 574 | 992 | 1,257 | 918 | 1,664 |
| Other malignant neoplasms | 4,065 | 3,234 | 5,079 | 4,426 | 3,464 | 5,543 |
| Other maternal disorders | 239 | 175 | 313 | 0 | 0 | 0 |
| Other neonatal disorders | 1,238 | 945 | 1,577 | 1,679 | 1,316 | 2,097 |
| Other non-rheumatic valve diseases | 13 | 8 | 20 | 11 | 7 | 16 |
| Other pneumoconiosis | 7 | 5 | 11 | 30 | 17 | 42 |
| Other road injuries | 277 | 210 | 359 | 172 | 137 | 212 |
| Other sexually transmitted infections | 123 | 78 | 186 | 7 | 5 | 11 |
| Other urinary diseases | 115 | 85 | 154 | 223 | 126 | 311 |
| Paratyphoid fever | 1 | 0 | 1 | 0 | 0 | 1 |
| Pedestrian road injuries | 1,434 | 1,184 | 1,717 | 3,702 | 3,016 | 4,501 |
| Peptic ulcer disease | 786 | 629 | 973 | 963 | 713 | 1,290 |
| Physical violence by firearm | 1,524 | 1,196 | 1,919 | 5,867 | 4,606 | 7,288 |
| Physical violence by other means | 1,435 | 1,192 | 1,716 | 2,418 | 1,938 | 3,004 |
| Physical violence by sharp object | 440 | 348 | 551 | 1,250 | 1,000 | 1,530 |
| Poisoning by carbon monoxide | 319 | 248 | 407 | 816 | 616 | 1,053 |
| Poisoning by other means | 513 | 380 | 670 | 540 | 417 | 694 |
| Pulmonary aspiration and foreign body in airway | 1,533 | 1,287 | 1,834 | 2,557 | 2,136 | 3,029 |
| Pyoderma | 287 | 147 | 508 | 306 | 100 | 699 |
| Self-harm by firearm | 2,912 | 2,157 | 3,745 | 20,097 | 15,187 | 26,984 |
| Self-harm by other specified means | 8,178 | 6,240 | 10,383 | 16,029 | 10,224 | 20,888 |
| Sickle cell disorders | 59 | 39 | 90 | 42 | 30 | 57 |
| Silicosis | 7 | 4 | 10 | 95 | 68 | 132 |
| Subarachnoid hemorrhage | 4,548 | 3,701 | 5,505 | 3,199 | 2,461 | 4,090 |
| Syphilis | 17 | 12 | 23 | 48 | 36 | 61 |
| Thalassemias | 58 | 42 | 81 | 33 | 25 | 42 |
| Thyroid cancer | 472 | 365 | 591 | 528 | 375 | 677 |
| Unintentional firearm injuries | 150 | 115 | 197 | 520 | 390 | 683 |
| Urinary tract infections and interstitial nephritis | 2,518 | 1,980 | 3,179 | 1,509 | 1,134 | 2,002 |
| Urogenital congenital anomalies | 152 | 105 | 231 | 270 | 175 | 395 |
| Urolithiasis | 155 | 118 | 198 | 140 | 102 | 185 |
| Uterine fibroids | 821 | 395 | 1,585 | 0 | 0 | 0 |

| | | | | | | |
|---|---|---|---|---|---|---|
| Venomous animal contact | 134 | 91 | 186 | 145 | 103 | 197 |
| Visceral leishmaniasis | 0 | 0 | 0 | 0 | 0 | 0 |
| **Communicable, maternal, neonatal, and nutritional diseases** | 32,970 | 28,077 | 38,789 | 34,868 | 30,262 | 39,992 |
| **Injuries** | 65,411 | 53,186 | 79,692 | 117,894 | 96,840 | 142,776 |
| **Non-communicable diseases** | 706,486 | 581,790 | 833,644 | 718,861 | 591,060 | 855,254 |
| **All causes** | **804,867** | **666,040** | **950,057** | **871,624** | **719,839** | **1,037,227** |

Figure S4. Arrow diagram of top ranked causes of disability adjusted life years (DALYs), in Colorado, in (A) both sexes (B) females and (C) males, Level 4, age standardized, 1990 - 2019

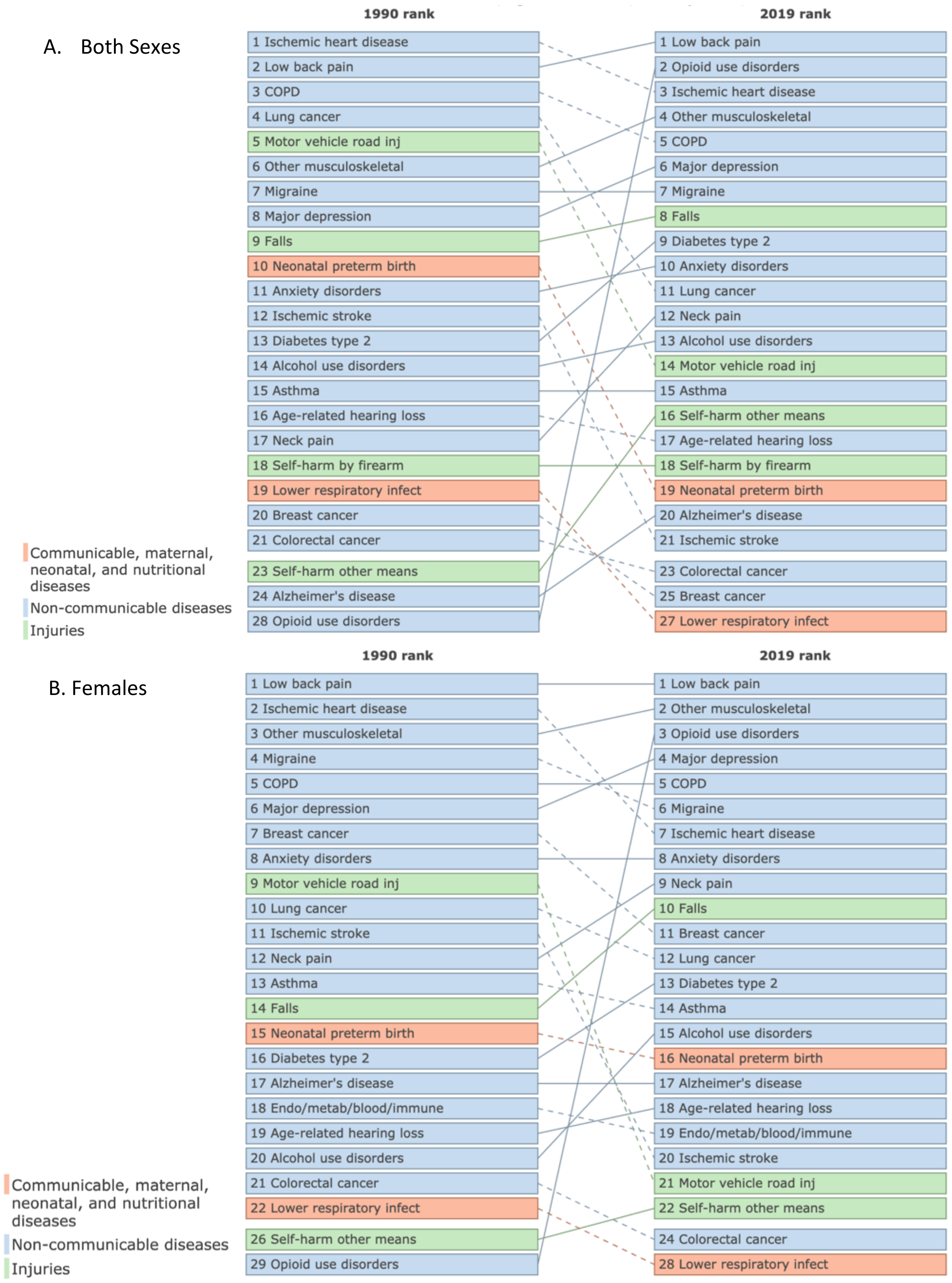

C. Males

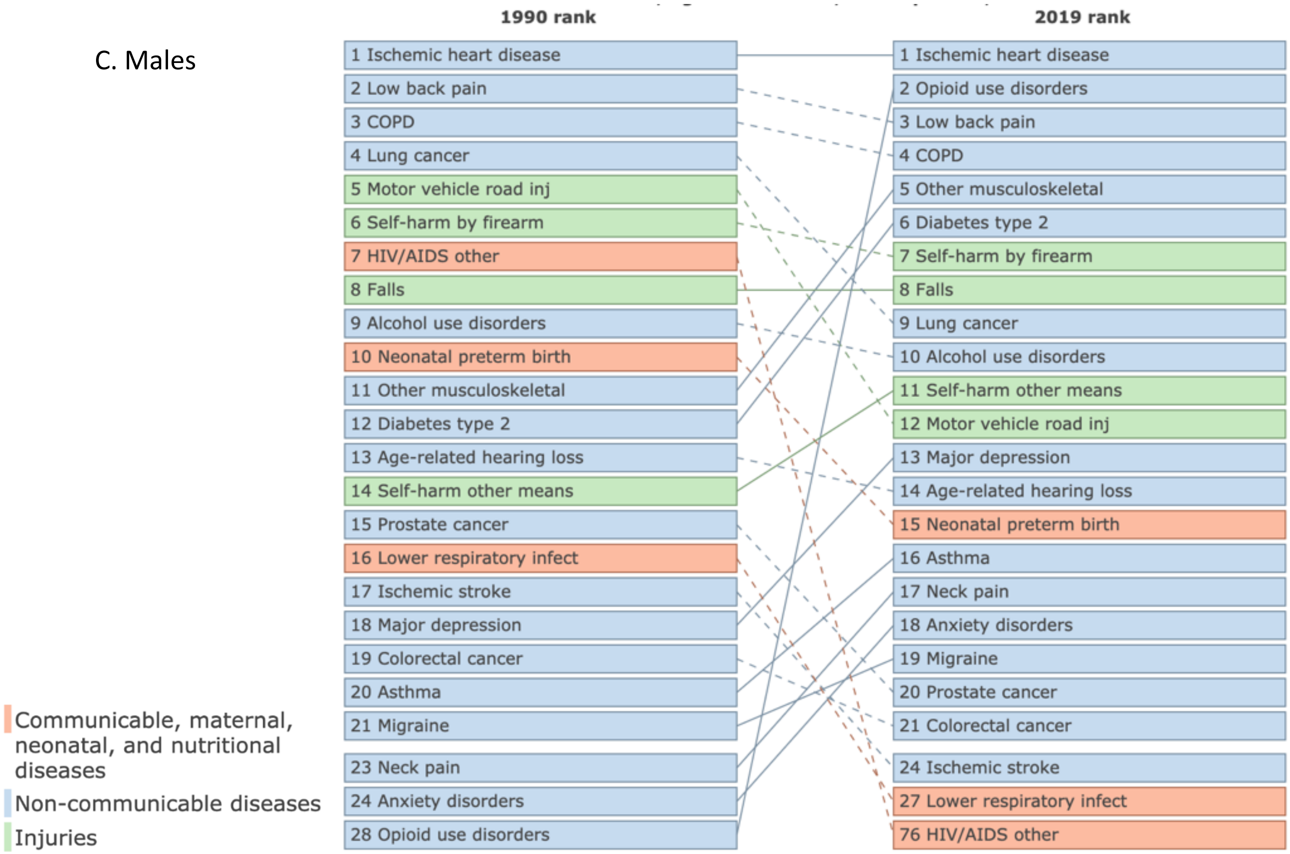
1990 rank
2019 rank
1 Ischemic heart disease
2 Low back pain
3 COPD
4 Lung cancer
5 Motor vehicle road inj
6 Self-harm by firearm
7 HIV/AIDS other
8 Falls
9 Alcohol use disorders
10 Neonatal preterm birth
11 Other musculoskeletal
12 Diabetes type 2
13 Age-related hearing loss
14 Self-harm other means
15 Prostate cancer
16 Lower respiratory infect
17 Ischemic stroke
18 Major depression
19 Colorectal cancer
20 Asthma
21 Migraine
23 Neck pain
24 Anxiety disorders
28 Opioid use disorders
1 Ischemic heart disease
2 Opioid use disorders
3 Low back pain
4 COPD
5 Other musculoskeletal
6 Diabetes type 2
7 Self-harm by firearm
8 Falls
9 Lung cancer
10 Alcohol use disorders
11 Self-harm other means
12 Motor vehicle road inj
13 Major depression
14 Age-related hearing loss
15 Neonatal preterm birth
16 Asthma
17 Neck pain
18 Anxiety disorders
19 Migraine
20 Prostate cancer
21 Colorectal cancer
24 Ischemic stroke
27 Lower respiratory infect
76 HIV/AIDS other
Communicable, maternal, neonatal, and nutritional diseases
Non-communicable diseases
Injuries

**Figure S5.** Line graph of age-standardized rates of (A) death and (B) disability adjusted life years (DALYs), per 100,000 people, in Colorado, West States, and the United States, in both sexes, 1990-2019

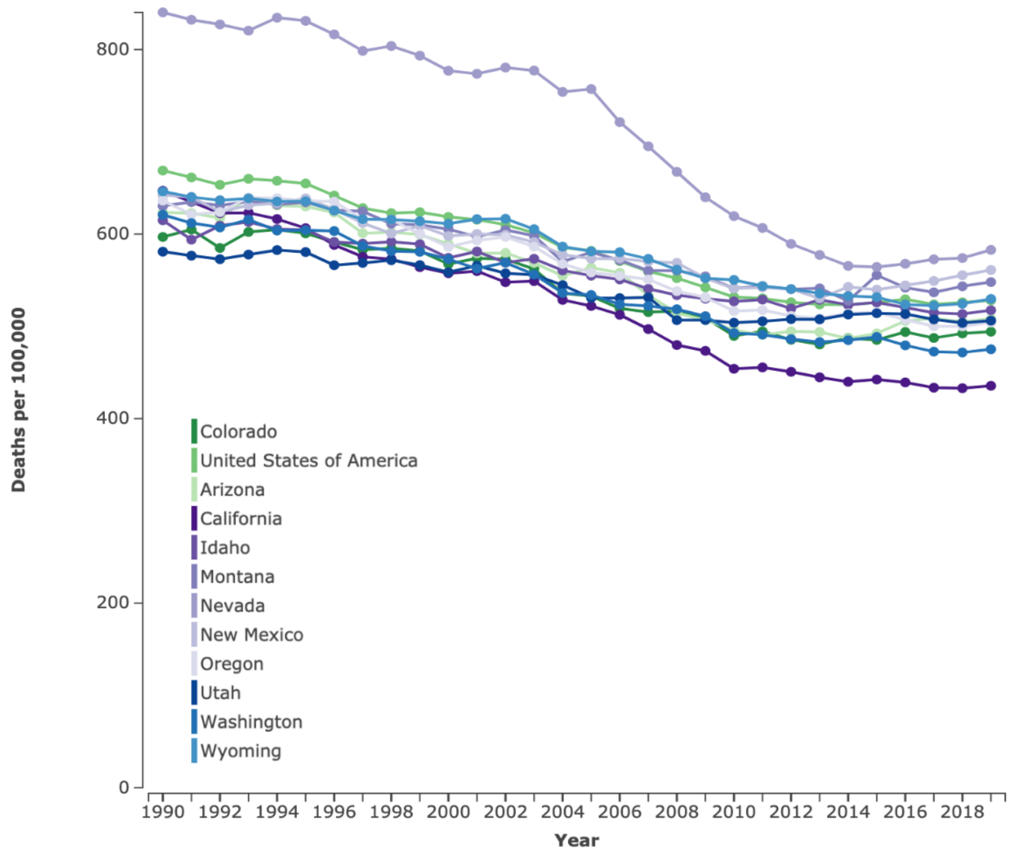


A. Deaths

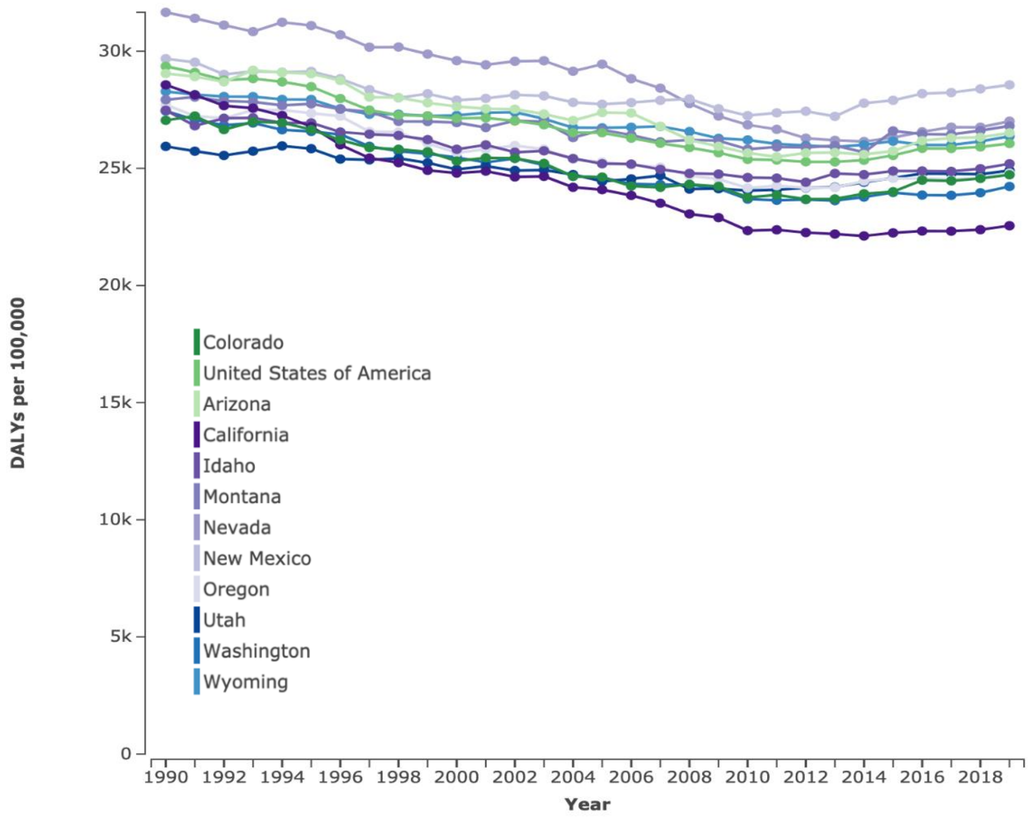


B. DALYs

**Figure S6.** Line graph of disability adjusted life years (DALYs), in Colorado, West States, and the United States by (A) females and (B) males, Level 4, age standardized, 1990 - 2019

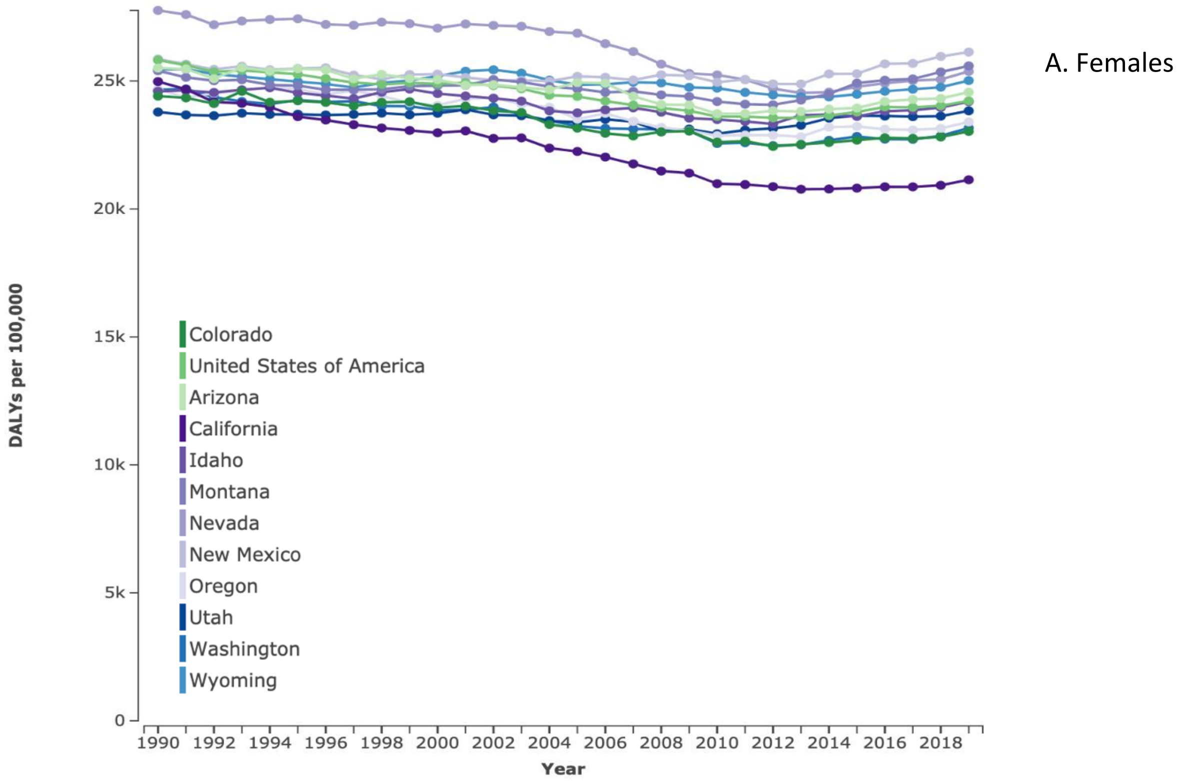


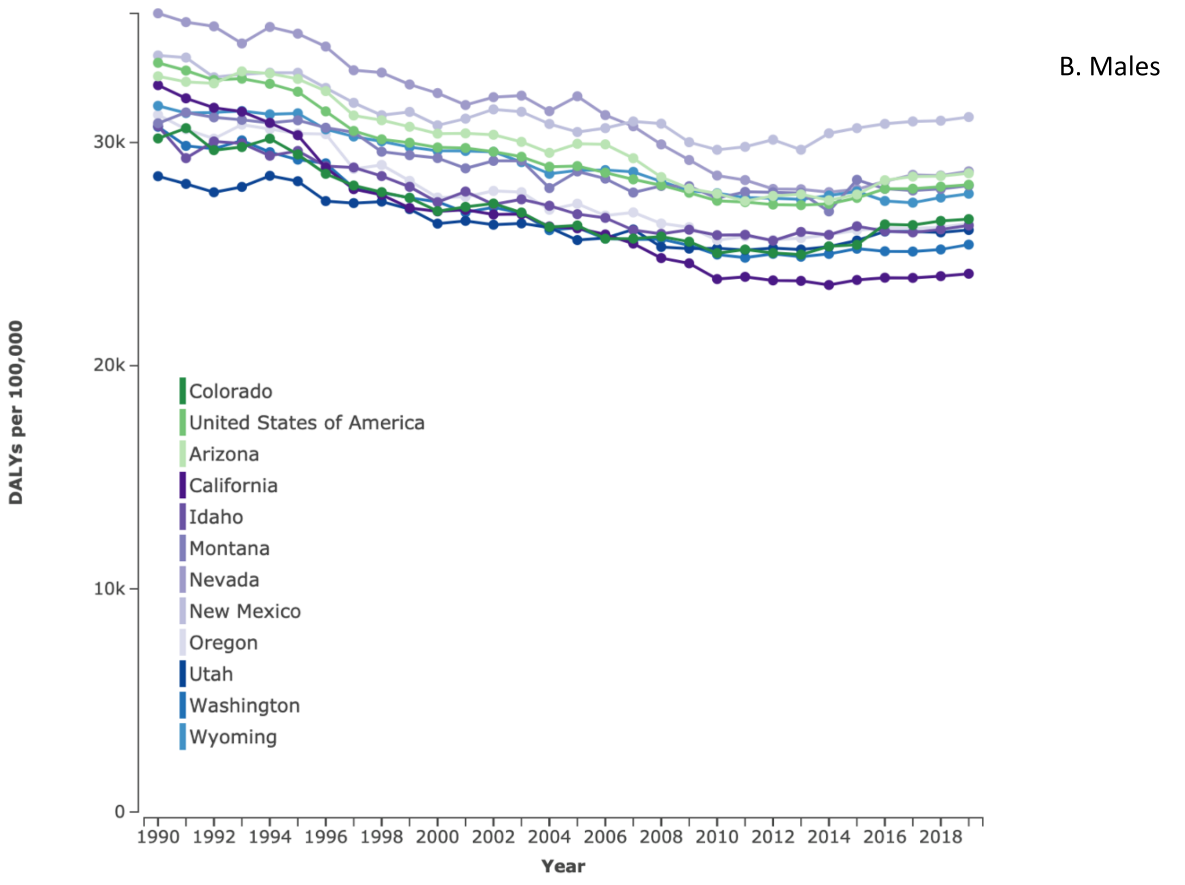

**Figure S7.** Heat map of top ranked causes of disability-adjusted life years (DALYS) in Colorado, West States, and the United States for (A) females (B) males, Level 4, age standardized, in 2019.*Numbers are rankings, with colors indicating the scale from low (blue: below 17th) to high (red: first and second) rankings.

A. Females

| | Colorado | United States of America | Arizona | California | Idaho | Montana | Nevada | New Mexico | Oregon | Utah | Washington | Wyoming |
|---|---|---|---|---|---|---|---|---|---|---|---|---|
| Low back pain | 1 | 1 | 1 | 1 | 1 | 1 | 1 | 1 | 1 | 1 | 1 | 1 |
| Other musculoskeletal | 2 | 2 | 3 | 2 | 2 | 2 | 4 | 3 | 2 | 4 | 2 | 2 |
| Opioid use disorders | 3 | 3 | 2 | 5 | 3 | 3 | 2 | 2 | 3 | 2 | 3 | 3 |
| Major depression | 4 | 5 | 4 | 6 | 4 | 5 | 6 | 4 | 4 | 3 | 4 | 5 |
| COPD | 5 | 6 | 6 | 8 | 5 | 4 | 5 | 6 | 6 | 8 | 6 | 4 |
| Migraine | 6 | 7 | 7 | 3 | 7 | 7 | 7 | 7 | 5 | 5 | 5 | 7 |
| Ischemic heart disease | 7 | 4 | 5 | 4 | 6 | 6 | 3 | 5 | 7 | 6 | 8 | 6 |
| Anxiety disorders | 8 | 8 | 8 | 7 | 9 | 9 | 9 | 9 | 11 | 9 | 9 | 8 |
| Neck pain | 9 | 11 | 10 | 9 | 8 | 10 | 10 | 10 | 9 | 10 | 7 | 15 |
| Falls | 10 | 14 | 14 | 14 | 13 | 14 | 14 | 11 | 13 | 11 | 12 | 13 |
| Breast cancer | 11 | 12 | 13 | 11 | 12 | 13 | 12 | 12 | 12 | 12 | 13 | 12 |
| Lung cancer | 12 | 10 | 12 | 13 | 11 | 8 | 8 | 15 | 8 | 18 | 10 | 9 |
| Diabetes type 2 | 13 | 9 | 9 | 10 | 10 | 12 | 11 | 8 | 10 | 7 | 11 | 10 |
| Asthma | 14 | 13 | 11 | 12 | 17 | 15 | 13 | 14 | 14 | 14 | 14 | 14 |
| Alcohol use disorders | 15 | 23 | 20 | 22 | 22 | 21 | 20 | 17 | 21 | 26 | 23 | 18 |
| Neonatal preterm birth | 16 | 15 | 16 | 18 | 19 | 18 | 16 | 18 | 20 | 19 | 19 | 21 |
| Alzheimer's disease | 17 | 20 | 21 | 19 | 20 | 20 | 22 | 20 | 18 | 22 | 18 | 20 |
| Age-related hearing loss | 18 | 18 | 18 | 15 | 18 | 19 | 18 | 19 | 17 | 16 | 17 | 19 |
| Endo/metab/blood/immune | 19 | 16 | 15 | 16 | 15 | 17 | 17 | 16 | 15 | 15 | 16 | 16 |
| Ischemic stroke | 20 | 17 | 19 | 17 | 16 | 16 | 15 | 21 | 16 | 13 | 15 | 17 |
| Motor vehicle road inj | 21 | 19 | 17 | 21 | 14 | 11 | 19 | 13 | 19 | 20 | 22 | 11 |
| Self-harm other means | 22 | 28 | 24 | 29 | 24 | 22 | 25 | 22 | 25 | 17 | 26 | 26 |
| Schizophrenia | 23 | 22 | 22 | 20 | 21 | 24 | 26 | 25 | 22 | 21 | 21 | 24 |
| Colorectal cancer | 24 | 21 | 23 | 23 | 23 | 23 | 21 | 23 | 23 | 24 | 24 | 22 |
| Osteoarthritis hand | 25 | 24 | 26 | 25 | 25 | 25 | 28 | 24 | 24 | 25 | 20 | 23 |

B. Males

| | Colorado | United States of America | Arizona | California | Idaho | Montana | Nevada | New Mexico | Oregon | Utah | Washington | Wyoming |
|---|---|---|---|---|---|---|---|---|---|---|---|---|
| Ischemic heart disease | 1 | 1 | 1 | 1 | 1 | 1 | 1 | 2 | 2 | 2 | 1 | 1 |
| Opioid use disorders | 2 | 2 | 2 | 3 | 2 | 2 | 2 | 1 | 1 | 1 | 2 | 2 |
| Low back pain | 3 | 3 | 3 | 2 | 3 | 3 | 3 | 3 | 3 | 3 | 3 | 3 |
| COPD | 4 | 4 | 5 | 5 | 4 | 4 | 4 | 5 | 4 | 5 | 6 | 4 |
| Other musculoskeletal | 5 | 7 | 6 | 6 | 5 | 7 | 7 | 7 | 6 | 6 | 4 | 6 |
| Diabetes type 2 | 6 | 5 | 4 | 4 | 6 | 8 | 6 | 4 | 5 | 4 | 5 | 8 |
| Self-harm by firearm | 7 | 14 | 9 | 28 | 7 | 6 | 8 | 9 | 9 | 7 | 13 | 7 |
| Falls | 8 | 11 | 13 | 14 | 12 | 11 | 16 | 12 | 10 | 12 | 8 | 11 |
| Lung cancer | 9 | 6 | 7 | 7 | 8 | 9 | 5 | 10 | 7 | 11 | 7 | 9 |
| Alcohol use disorders | 10 | 13 | 8 | 9 | 14 | 10 | 9 | 6 | 8 | 13 | 14 | 10 |
| Self-harm other means | 11 | 18 | 15 | 18 | 15 | 13 | 13 | 14 | 14 | 9 | 12 | 14 |
| Motor vehicle road inj | 12 | 8 | 10 | 10 | 9 | 5 | 12 | 8 | 13 | 15 | 16 | 5 |
| Major depression | 13 | 12 | 11 | 12 | 10 | 12 | 10 | 11 | 11 | 8 | 9 | 12 |
| Age-related hearing loss | 14 | 9 | 12 | 8 | 11 | 14 | 11 | 15 | 12 | 10 | 11 | 13 |
| Neonatal preterm birth | 15 | 10 | 19 | 19 | 18 | 18 | 20 | 16 | 15 | 14 | 18 | 15 |
| Asthma | 16 | 19 | 14 | 15 | 23 | 20 | 18 | 18 | 20 | 22 | 20 | 21 |
| Neck pain | 17 | 16 | 16 | 13 | 13 | 26 | 17 | 26 | 17 | 25 | 10 | 23 |
| Anxiety disorders | 18 | 20 | 18 | 16 | 16 | 15 | 19 | 20 | 26 | 16 | 15 | 16 |
| Migraine | 19 | 21 | 20 | 17 | 17 | 16 | 22 | 21 | 16 | 17 | 17 | 17 |
| Prostate cancer | 20 | 22 | 24 | 21 | 19 | 19 | 23 | 23 | 18 | 18 | 19 | 19 |
| Colorectal cancer | 21 | 17 | 21 | 20 | 21 | 17 | 15 | 17 | 21 | 20 | 21 | 18 |
| Alzheimer's disease | 22 | 25 | 26 | 26 | 25 | 23 | 29 | 27 | 24 | 29 | 25 | 28 |
| Cirrhosis hepatitis C | 23 | 30 | 23 | 23 | 27 | 24 | 25 | 13 | 23 | 31 | 26 | 25 |
| Ischemic stroke | 24 | 23 | 25 | 22 | 20 | 21 | 26 | 24 | 19 | 19 | 22 | 20 |
| Schizophrenia | 25 | 27 | 27 | 24 | 24 | 25 | 28 | 30 | 25 | 23 | 24 | 26 |

**Figure S8.** Heat map of top ranked causes of disability-adjusted life years (DALYS) in Colorado, West States, and the United States, for both sexes, Level 4, age standardized, in 1990.*Numbers are rankings, with colors indicating the scale from low (blue: below 17th) to high (red: first and second) rankings.

| | **Colorado** | United States of America | Arizona | California | Idaho | Montana | Nevada | New Mexico | Oregon | Utah | Washington | Wyoming |
|---|---|---|---|---|---|---|---|---|---|---|---|---|
| Ischemic heart disease | 1 | 1 | 1 | 1 | 1 | 1 | 1 | 1 | 1 | 1 | 1 | 1 |
| Low back pain | 2 | 2 | 2 | 2 | 2 | 2 | 2 | 2 | 2 | 2 | 2 | 2 |
| COPD | 3 | 4 | 4 | 4 | 4 | 4 | 4 | 4 | 4 | 3 | 4 | 4 |
| Lung cancer | 4 | 3 | 3 | 3 | 5 | 5 | 3 | 5 | 3 | 10 | 3 | 5 |
| Motor vehicle road inj | 5 | 5 | 5 | 6 | 3 | 3 | 5 | 3 | 5 | 4 | 5 | 3 |
| Other musculoskeletal | 6 | 7 | 6 | 7 | 6 | 6 | 7 | 7 | 6 | 6 | 6 | 6 |
| Migraine | 7 | 8 | 7 | 8 | 7 | 7 | 9 | 9 | 7 | 8 | 7 | 7 |
| Major depression | 8 | 12 | 8 | 21 | 8 | 8 | 10 | 10 | 9 | 7 | 8 | 9 |
| Falls | 9 | 16 | 16 | 19 | 15 | 14 | 20 | 13 | 11 | 12 | 12 | 14 |
| Neonatal preterm birth | 10 | 6 | 9 | 9 | 13 | 17 | 15 | 11 | 16 | 18 | 15 | 15 |
| Anxiety disorders | 11 | 13 | 13 | 12 | 14 | 12 | 14 | 15 | 19 | 11 | 13 | 12 |
| Ischemic stroke | 12 | 10 | 14 | 10 | 9 | 9 | 8 | 14 | 8 | 9 | 9 | 10 |
| Diabetes type 2 | 13 | 9 | 10 | 11 | 10 | 10 | 11 | 6 | 10 | 5 | 10 | 11 |
| Alcohol use disorders | 14 | 22 | 11 | 18 | 19 | 13 | 13 | 8 | 12 | 21 | 19 | 13 |
| Asthma | 15 | 19 | 15 | 16 | 17 | 16 | 18 | 16 | 15 | 16 | 16 | 18 |
| Age-related hearing loss | 16 | 17 | 17 | 15 | 16 | 15 | 17 | 17 | 14 | 14 | 14 | 17 |
| Neck pain | 17 | 18 | 18 | 20 | 12 | 19 | 21 | 21 | 13 | 20 | 11 | 25 |
| Self-harm by firearm | 18 | 25 | 12 | 26 | 11 | 11 | 6 | 12 | 17 | 13 | 20 | 8 |
| Lower respiratory infect | 19 | 20 | 19 | 14 | 20 | 21 | 12 | 19 | 21 | 15 | 21 | 16 |
| Breast cancer | 20 | 14 | 20 | 17 | 18 | 18 | 19 | 20 | 18 | 19 | 17 | 19 |
| Colorectal cancer | 21 | 15 | 21 | 23 | 21 | 20 | 16 | 23 | 20 | 23 | 18 | 20 |
| HIV/AIDS other | 22 | 11 | 25 | 5 | 68 | 75 | 27 | 31 | 28 | 40 | 25 | 85 |
| Self-harm other means | 23 | 29 | 28 | 29 | 27 | 23 | 22 | 22 | 26 | 17 | 22 | 27 |
| Alzheimer's disease | 24 | 23 | 24 | 24 | 23 | 22 | 29 | 25 | 22 | 24 | 23 | 22 |
| SIDS | 25 | 30 | 27 | 28 | 25 | 24 | 25 | 29 | 23 | 26 | 24 | 21 |

Figure S9. Pyramid of disability adjusted life years (DALYs) in Colorado and the United States, by sex and all ages in 2019

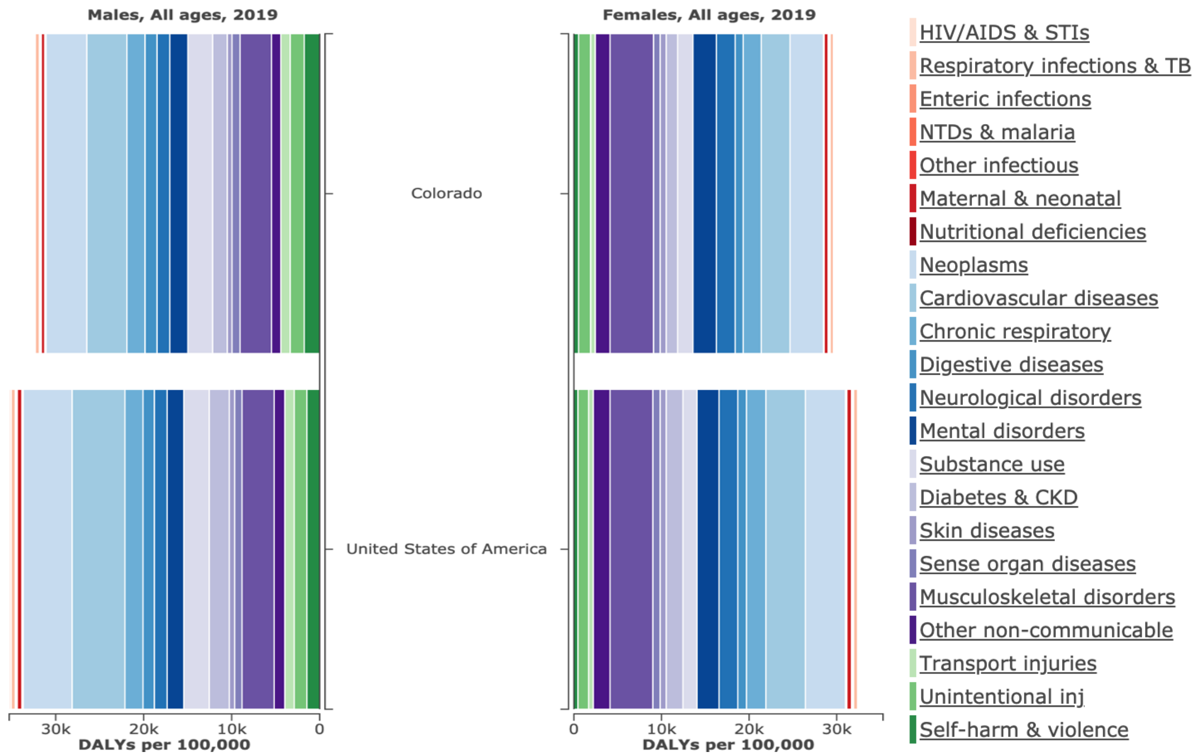

Figure S10. Pyramid of disability adjusted life years (DALYs) in Colorado, Earth and the United States, by sex and all ages in 2019

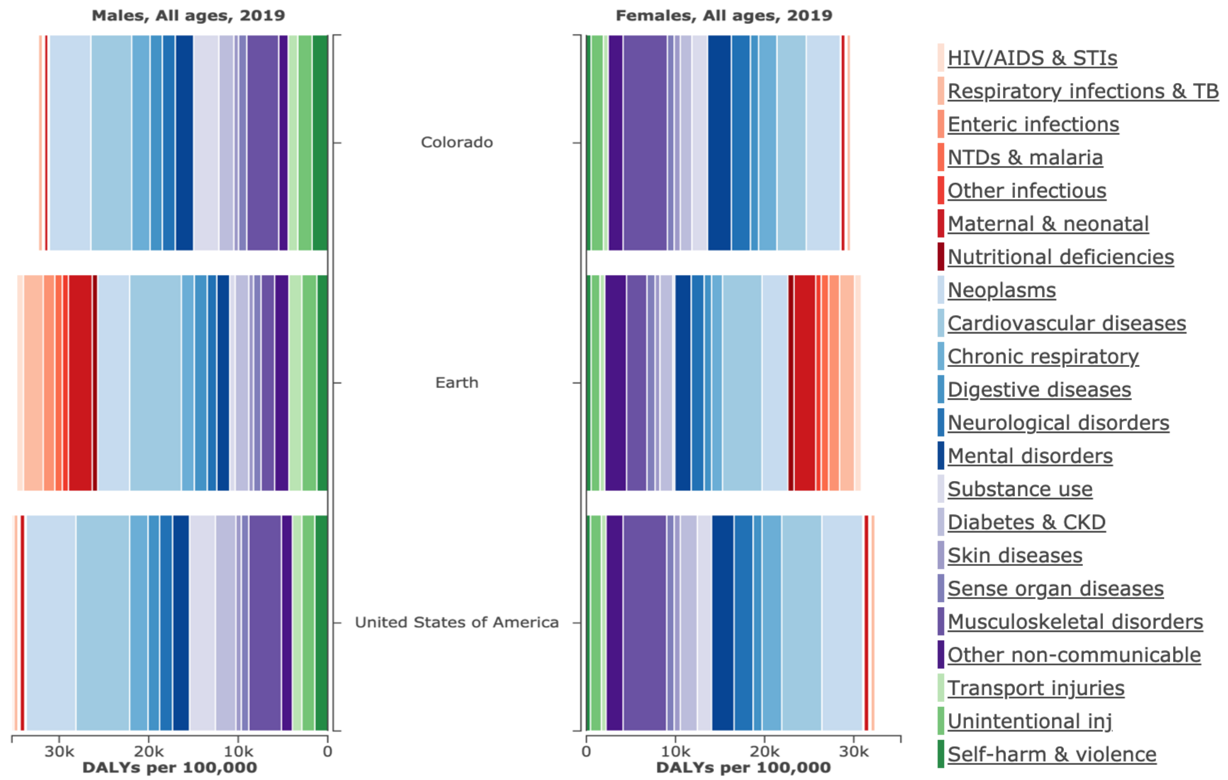

**Figure S11.** Heat map of top ranked years of life lost (YLL) in Colorado, West states, and the United States in (A) both sexes (B) females and (C) males, Level 4, age standardized, in 2019.*Numbers are rankings, with colors indicating the scale from low (blue: below 17th) to high (red: first and second) rankings.

A. Both Sexes

| | Colorado | United States of America | Arizona | California | Idaho | Montana | Nevada | New Mexico | Oregon | Utah | Washington | Wyoming |
|---|---|---|---|---|---|---|---|---|---|---|---|---|
| Ischemic heart disease | 1 | 1 | 1 | 1 | 1 | 1 | 1 | 1 | 1 | 1 | 1 | 1 |
| Opioid use disorders | 2 | 3 | 2 | 3 | 4 | 5 | 3 | 2 | 3 | 2 | 3 | 3 |
| COPD | 3 | 4 | 4 | 4 | 2 | 4 | 4 | 3 | 4 | 3 | 4 | 2 |
| Lung cancer | 4 | 2 | 3 | 2 | 3 | 2 | 2 | 4 | 2 | 6 | 2 | 5 |
| Self-harm other means | 5 | 8 | 7 | 6 | 7 | 7 | 6 | 7 | 5 | 4 | 5 | 7 |
| Self-harm by firearm | 6 | 10 | 6 | 20 | 6 | 6 | 5 | 6 | 6 | 5 | 7 | 6 |
| Motor vehicle road inj | 7 | 5 | 5 | 5 | 5 | 3 | 9 | 5 | 7 | 8 | 8 | 4 |
| Colorectal cancer | 8 | 6 | 8 | 7 | 8 | 8 | 7 | 10 | 8 | 9 | 6 | 8 |
| Neonatal preterm birth | 9 | 7 | 12 | 13 | 11 | 12 | 14 | 12 | 11 | 11 | 12 | 10 |
| Alzheimer's disease | 10 | 16 | 17 | 14 | 14 | 13 | 18 | 17 | 15 | 17 | 11 | 15 |
| Cirrhosis hepatitis C | 11 | 19 | 11 | 12 | 17 | 14 | 12 | 9 | 13 | 19 | 16 | 14 |
| Breast cancer | 12 | 11 | 13 | 9 | 10 | 9 | 11 | 15 | 10 | 12 | 10 | 11 |
| Lower respiratory infect | 13 | 13 | 14 | 11 | 13 | 11 | 10 | 13 | 19 | 10 | 17 | 9 |
| Alcohol use disorders | 14 | 25 | 15 | 24 | 23 | 17 | 20 | 8 | 17 | 20 | 24 | 12 |
| Falls | 15 | 22 | 22 | 32 | 19 | 19 | 26 | 19 | 20 | 18 | 19 | 20 |
| Pancreatic cancer | 16 | 15 | 16 | 15 | 15 | 15 | 16 | 18 | 14 | 14 | 13 | 16 |
| Ischemic stroke | 17 | 17 | 20 | 17 | 12 | 16 | 17 | 21 | 12 | 13 | 15 | 17 |
| Diabetes type 2 | 18 | 9 | 9 | 10 | 9 | 10 | 19 | 11 | 9 | 7 | 9 | 13 |
| Intracerebral hem | 19 | 14 | 21 | 16 | 18 | 20 | 15 | 22 | 18 | 16 | 18 | 19 |
| Endo/metab/blood/immune | 20 | 20 | 18 | 19 | 16 | 18 | 21 | 16 | 16 | 15 | 14 | 18 |
| Violence firearm | 21 | 12 | 10 | 8 | 43 | 29 | 13 | 14 | 26 | 45 | 25 | 31 |
| Brain cancer | 22 | 21 | 25 | 21 | 20 | 21 | 25 | 27 | 21 | 21 | 20 | 21 |
| Hypertensive heart disease | 23 | 18 | 19 | 18 | 25 | 26 | 8 | 25 | 36 | 26 | 21 | 25 |
| Prostate cancer | 24 | 24 | 26 | 23 | 21 | 22 | 22 | 28 | 22 | 22 | 23 | 22 |
| Other malignant neoplasms | 25 | 23 | 27 | 22 | 22 | 23 | 27 | 26 | 23 | 23 | 22 | 23 |

B. Females

| | Colorado | United States of America | Arizona | California | Idaho | Montana | Nevada | New Mexico | Oregon | Utah | Washington | Wyoming |
|---|---|---|---|---|---|---|---|---|---|---|---|---|
| Ischemic heart disease | 1 | 1 | 1 | 1 | 1 | 1 | 1 | 1 | 1 | 1 | 1 | 1 |
| COPD | 2 | 3 | 3 | 4 | 2 | 3 | 3 | 3 | 3 | 3 | 3 | 2 |
| Lung cancer | 3 | 2 | 2 | 2 | 3 | 2 | 2 | 4 | 2 | 6 | 2 | 3 |
| Breast cancer | 4 | 4 | 4 | 3 | 4 | 5 | 4 | 5 | 4 | 2 | 4 | 4 |
| Opioid use disorders | 5 | 5 | 5 | 6 | 6 | 6 | 5 | 2 | 5 | 4 | 5 | 6 |
| Self-harm other means | 6 | 13 | 7 | 11 | 8 | 7 | 8 | 7 | 8 | 5 | 8 | 12 |
| Alzheimer's disease | 7 | 9 | 11 | 7 | 10 | 9 | 13 | 11 | 9 | 14 | 7 | 9 |
| Motor vehicle road inj | 8 | 7 | 6 | 8 | 5 | 4 | 10 | 6 | 6 | 10 | 10 | 5 |
| Colorectal cancer | 9 | 8 | 8 | 5 | 7 | 8 | 6 | 8 | 7 | 11 | 6 | 7 |
| Neonatal preterm birth | 10 | 6 | 9 | 10 | 11 | 10 | 12 | 10 | 13 | 12 | 12 | 10 |
| Ischemic stroke | 11 | 12 | 13 | 12 | 9 | 13 | 11 | 16 | 10 | 9 | 9 | 11 |
| Lower respiratory infect | 12 | 10 | 12 | 9 | 13 | 11 | 7 | 13 | 17 | 8 | 16 | 8 |
| Pancreatic cancer | 13 | 14 | 14 | 14 | 14 | 14 | 15 | 17 | 14 | 15 | 14 | 14 |
| Ovarian cancer | 14 | 17 | 17 | 15 | 16 | 16 | 16 | 18 | 15 | 16 | 15 | 17 |
| Diabetes type 2 | 15 | 11 | 10 | 13 | 12 | 12 | 18 | 9 | 11 | 7 | 11 | 13 |
| Endo/metab/blood/immune | 16 | 16 | 15 | 17 | 15 | 15 | 20 | 15 | 12 | 13 | 13 | 15 |
| Intracerebral hem | 17 | 15 | 18 | 16 | 17 | 18 | 14 | 19 | 16 | 17 | 17 | 16 |
| Falls | 18 | 28 | 21 | 43 | 22 | 20 | 35 | 20 | 20 | 18 | 21 | 24 |
| Alcohol use disorders | 19 | 39 | 23 | 35 | 31 | 21 | 21 | 14 | 22 | 28 | 26 | 20 |
| Cirrhosis hepatitis C | 20 | 20 | 16 | 19 | 19 | 19 | 17 | 12 | 18 | 24 | 18 | 19 |
| Other malignant neoplasms | 21 | 19 | 22 | 20 | 20 | 22 | 22 | 23 | 19 | 21 | 19 | 22 |
| Brain cancer | 22 | 21 | 24 | 22 | 21 | 23 | 26 | 27 | 21 | 23 | 20 | 21 |
| Self-harm by firearm | 23 | 33 | 20 | 52 | 18 | 17 | 19 | 21 | 23 | 19 | 27 | 18 |
| Other cardiovascular | 24 | 25 | 31 | 30 | 25 | 29 | 29 | 31 | 28 | 25 | 24 | 23 |
| Hypertensive heart disease | 25 | 18 | 19 | 18 | 23 | 28 | 9 | 25 | 33 | 22 | 22 | 28 |

C. Males

| | Colorado | United States of America | Arizona | California | Idaho | Montana | Nevada | New Mexico | Oregon | Utah | Washington | Wyoming |
|---|---|---|---|---|---|---|---|---|---|---|---|---|
| Ischemic heart disease | 1 | 1 | 1 | 1 | 1 | 1 | 1 | 1 | 1 | 1 | 1 | 1 |
| Opioid use disorders | 2 | 2 | 2 | 3 | 5 | 4 | 2 | 2 | 2 | 2 | 2 | 2 |
| COPD | 3 | 4 | 4 | 4 | 4 | 6 | 4 | 5 | 4 | 4 | 4 | 5 |
| Self-harm by firearm | 4 | 6 | 5 | 12 | 2 | 2 | 5 | 3 | 5 | 3 | 5 | 3 |
| Lung cancer | 5 | 3 | 3 | 2 | 3 | 5 | 3 | 6 | 3 | 6 | 3 | 6 |
| Self-harm other means | 6 | 8 | 7 | 6 | 7 | 7 | 6 | 9 | 6 | 5 | 6 | 7 |
| Motor vehicle road inj | 7 | 5 | 6 | 7 | 6 | 3 | 9 | 4 | 7 | 8 | 7 | 4 |
| Colorectal cancer | 8 | 9 | 11 | 8 | 8 | 8 | 8 | 10 | 9 | 9 | 8 | 8 |
| Cirrhosis hepatitis C | 9 | 14 | 10 | 9 | 12 | 11 | 12 | 8 | 10 | 17 | 11 | 11 |
| Alcohol use disorders | 10 | 21 | 12 | 20 | 21 | 12 | 16 | 7 | 12 | 14 | 22 | 9 |
| Prostate cancer | 11 | 13 | 13 | 11 | 9 | 10 | 13 | 16 | 11 | 10 | 10 | 13 |
| Neonatal preterm birth | 12 | 10 | 14 | 15 | 11 | 16 | 17 | 13 | 13 | 12 | 13 | 10 |
| Falls | 13 | 22 | 19 | 27 | 19 | 14 | 23 | 17 | 18 | 19 | 16 | 16 |
| Diabetes type 2 | 14 | 11 | 9 | 10 | 10 | 9 | 18 | 11 | 8 | 7 | 9 | 14 |
| Lower respiratory infect | 15 | 12 | 15 | 13 | 14 | 13 | 11 | 14 | 20 | 11 | 17 | 12 |
| Pancreatic cancer | 16 | 16 | 16 | 17 | 13 | 15 | 15 | 18 | 14 | 13 | 12 | 15 |
| Violence firearm | 17 | 7 | 8 | 5 | 35 | 30 | 10 | 12 | 25 | 34 | 21 | 28 |
| Alzheimer's disease | 18 | 20 | 23 | 19 | 18 | 18 | 20 | 23 | 19 | 22 | 19 | 20 |
| Intracerebral hem | 19 | 15 | 20 | 14 | 17 | 20 | 14 | 22 | 16 | 18 | 15 | 18 |
| Ischemic stroke | 20 | 19 | 24 | 21 | 15 | 19 | 19 | 25 | 17 | 16 | 18 | 19 |
| Endo/metab/blood/immune | 21 | 18 | 17 | 18 | 16 | 17 | 22 | 19 | 15 | 15 | 14 | 17 |
| Cirrhosis alcohol | 22 | 26 | 21 | 22 | 25 | 24 | 21 | 15 | 21 | 32 | 25 | 23 |
| Hypertensive heart disease | 23 | 17 | 18 | 16 | 27 | 29 | 7 | 24 | 40 | 27 | 24 | 27 |
| Brain cancer | 24 | 23 | 26 | 23 | 20 | 21 | 26 | 28 | 22 | 20 | 20 | 21 |
| Esophageal cancer | 25 | 24 | 27 | 30 | 23 | 23 | 24 | 29 | 23 | 30 | 23 | 22 |

**Figure S12.** Heat map of top ranked years of life lost (YLL) in Colorado, West States, and the United States, both sexes, Level 4, age standardized in 1990.*Numbers are rankings, with colors indicating the scale from low (blue: below 17th) to high (red: first and second) rankings.

| | **Colorado** | United States of America | Arizona | California | Idaho | Montana | Nevada | New Mexico | Oregon | Utah | Washington | Wyoming |
|---|---|---|---|---|---|---|---|---|---|---|---|---|
| Ischemic heart disease | 1 | 1 | 1 | 1 | 1 | 1 | 1 | 1 | 1 | 1 | 1 | 1 |
| Lung cancer | 2 | 2 | 2 | 2 | 3 | 3 | 2 | 3 | 2 | 3 | 2 | 3 |
| Motor vehicle road inj | 3 | 3 | 3 | 4 | 2 | 2 | 4 | 2 | 3 | 2 | 3 | 2 |
| COPD | 4 | 5 | 4 | 5 | 4 | 4 | 3 | 4 | 4 | 5 | 4 | 4 |
| Self-harm by firearm | 5 | 12 | 5 | 12 | 5 | 5 | 5 | 5 | 6 | 4 | 7 | 5 |
| Lower respiratory infect | 6 | 9 | 7 | 8 | 8 | 8 | 6 | 7 | 9 | 6 | 9 | 6 |
| Neonatal preterm birth | 7 | 4 | 6 | 7 | 7 | 10 | 13 | 6 | 11 | 13 | 12 | 8 |
| Self-harm other means | 8 | 15 | 14 | 14 | 12 | 12 | 10 | 8 | 12 | 8 | 10 | 12 |
| Colorectal cancer | 9 | 7 | 9 | 11 | 10 | 7 | 8 | 11 | 7 | 11 | 8 | 9 |
| HIV/AIDS other | 10 | 6 | 11 | 3 | 44 | 47 | 15 | 20 | 13 | 23 | 13 | 52 |
| Breast cancer | 11 | 8 | 8 | 10 | 9 | 9 | 9 | 12 | 8 | 10 | 6 | 11 |
| Ischemic stroke | 12 | 10 | 10 | 9 | 6 | 6 | 7 | 10 | 5 | 7 | 5 | 7 |
| SIDS | 13 | 16 | 12 | 13 | 11 | 11 | 12 | 15 | 10 | 12 | 11 | 10 |
| Alzheimer's disease | 14 | 17 | 18 | 18 | 14 | 14 | 22 | 18 | 15 | 15 | 15 | 14 |
| Diabetes type 2 | 15 | 13 | 16 | 17 | 13 | 13 | 17 | 9 | 14 | 9 | 14 | 13 |
| Congenital heart | 16 | 18 | 17 | 16 | 15 | 17 | 24 | 19 | 19 | 16 | 17 | 16 |
| Intracerebral hem | 17 | 14 | 19 | 15 | 16 | 15 | 16 | 21 | 16 | 14 | 16 | 15 |
| Prostate cancer | 18 | 20 | 22 | 23 | 17 | 16 | 21 | 23 | 18 | 17 | 20 | 17 |
| Pancreatic cancer | 19 | 19 | 21 | 21 | 19 | 18 | 20 | 22 | 17 | 19 | 18 | 18 |
| Violence firearm | 20 | 11 | 13 | 6 | 37 | 23 | 11 | 14 | 25 | 37 | 19 | 26 |
| Lymphoma | 21 | 21 | 23 | 24 | 20 | 19 | 25 | 31 | 20 | 20 | 22 | 23 |
| Brain cancer | 22 | 25 | 26 | 28 | 21 | 20 | 29 | 34 | 21 | 21 | 21 | 22 |
| Neonatal encephalopathy | 23 | 29 | 31 | 34 | 25 | 24 | 39 | 35 | 26 | 34 | 30 | 19 |
| Cirrhosis hepatitis C | 24 | 24 | 20 | 19 | 32 | 26 | 18 | 17 | 27 | 24 | 24 | 21 |
| Other malignant neoplasms | 25 | 27 | 27 | 29 | 23 | 21 | 30 | 29 | 23 | 22 | 23 | 24 |

**Figure S13.** Tree map of top causes of (A) years of healthy life lost due to disability (YLDs) and (B) years of life lost (YLLs) in Colorado, for both sexes, Level 4, all ages, in 2019.*Blue rectangles are non-communicable disease; red rectangles are communicable; and green rectangles are injuries.

A. YLDs

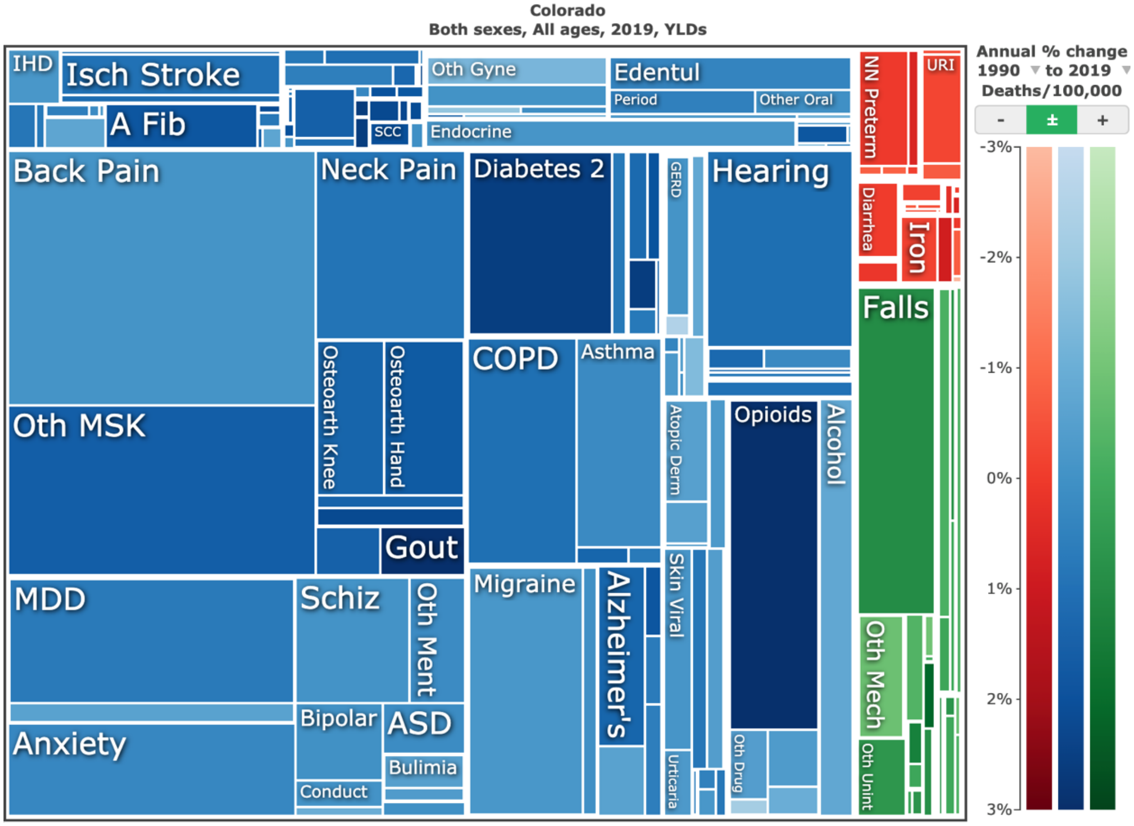


A. YLLs

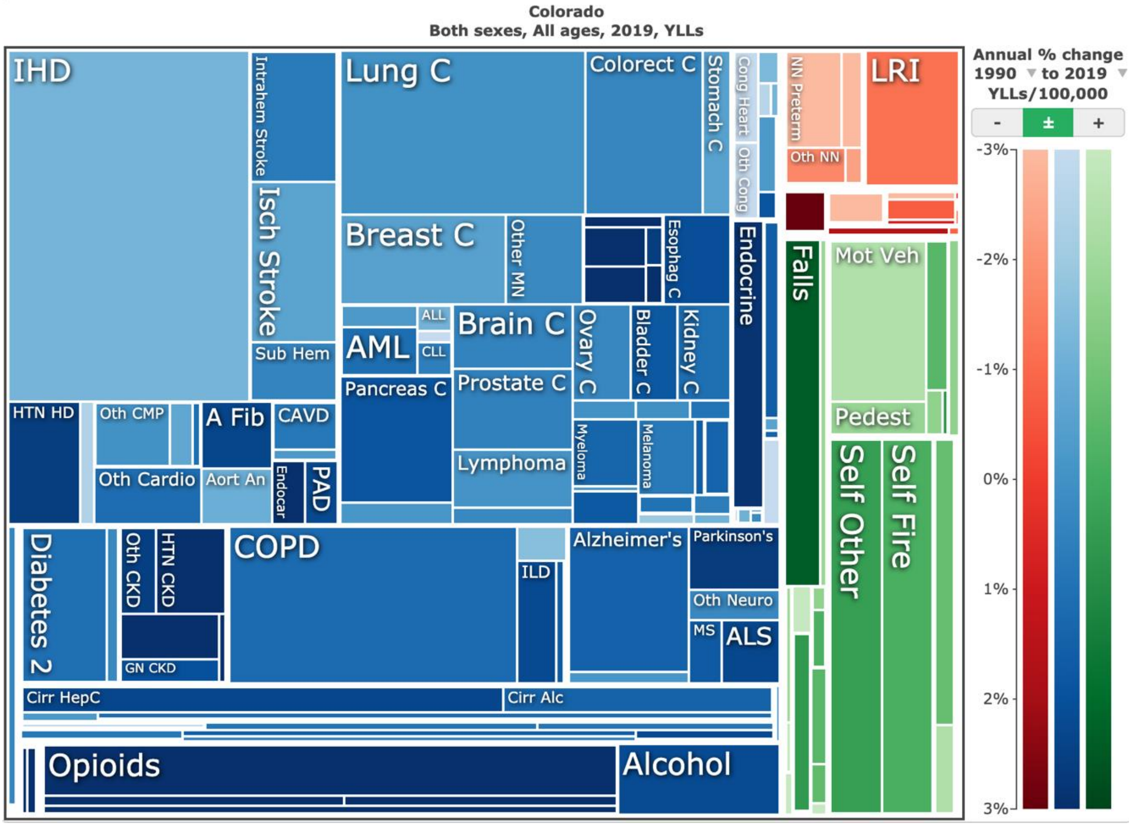

**Figure S14.** Heat map of top ranked causes of years of healthy life lost due to disability (YLDs) per 100,000 people, in Colorado, West States, and the United States for (A) both sexes, (B) females and (C) males, Level 4, age-standardized, in 2019. *Numbers are rankings, with colors indicating the scale from low (blue: below 17th) to high (red: first and second) rankings.

B. Both Sexes

| | Colorado | United States of America | Arizona | California | Idaho | Montana | Nevada | New Mexico | Oregon | Utah | Washington | Wyoming |
|---|---|---|---|---|---|---|---|---|---|---|---|---|
| Low back pain | 1 | 1 | 1 | 1 | 1 | 1 | 1 | 1 | 1 | 1 | 1 | 1 |
| Other musculoskeletal | 2 | 2 | 2 | 2 | 2 | 2 | 3 | 3 | 2 | 3 | 2 | 2 |
| Major depression | 3 | 4 | 4 | 5 | 4 | 4 | 4 | 4 | 4 | 2 | 4 | 4 |
| Opioid use disorders | 4 | 3 | 3 | 4 | 3 | 3 | 2 | 2 | 3 | 4 | 3 | 3 |
| Migraine | 5 | 5 | 5 | 3 | 5 | 5 | 5 | 5 | 5 | 5 | 6 | 5 |
| Anxiety disorders | 6 | 7 | 7 | 6 | 7 | 6 | 6 | 7 | 9 | 6 | 7 | 6 |
| Neck pain | 7 | 8 | 6 | 7 | 6 | 7 | 7 | 8 | 6 | 9 | 5 | 11 |
| Age-related hearing loss | 8 | 9 | 10 | 9 | 9 | 9 | 9 | 9 | 8 | 8 | 9 | 7 |
| Falls | 9 | 12 | 12 | 11 | 10 | 10 | 12 | 11 | 10 | 10 | 10 | 10 |
| Asthma | 10 | 10 | 9 | 10 | 12 | 12 | 10 | 10 | 11 | 11 | 11 | 12 |
| Diabetes type 2 | 11 | 6 | 8 | 8 | 8 | 8 | 8 | 6 | 7 | 7 | 8 | 8 |
| COPD | 12 | 11 | 11 | 12 | 11 | 11 | 11 | 12 | 12 | 12 | 12 | 9 |
| Alcohol use disorders | 13 | 14 | 13 | 14 | 14 | 13 | 13 | 13 | 14 | 14 | 14 | 13 |
| Schizophrenia | 14 | 13 | 14 | 13 | 13 | 14 | 14 | 14 | 13 | 13 | 13 | 14 |
| Osteoarthritis hand | 15 | 15 | 16 | 15 | 16 | 15 | 16 | 15 | 15 | 15 | 15 | 15 |
| Endo/metab/blood/immune | 16 | 16 | 15 | 17 | 15 | 17 | 15 | 16 | 17 | 16 | 17 | 16 |
| Neonatal preterm birth | 17 | 18 | 17 | 20 | 21 | 19 | 17 | 18 | 19 | 21 | 19 | 22 |
| Osteoarthritis knee | 18 | 19 | 20 | 19 | 22 | 22 | 20 | 19 | 21 | 19 | 20 | 21 |
| Ischemic stroke | 19 | 17 | 18 | 18 | 18 | 16 | 18 | 17 | 16 | 17 | 16 | 17 |
| Bipolar disorder | 20 | 24 | 21 | 24 | 17 | 18 | 27 | 22 | 24 | 20 | 23 | 26 |
| Viral skin diseases | 21 | 23 | 28 | 22 | 19 | 23 | 32 | 29 | 20 | 18 | 22 | 27 |
| Other mental disorders | 22 | 22 | 22 | 21 | 23 | 21 | 21 | 21 | 22 | 23 | 21 | 20 |
| Atopic derm | 23 | 20 | 19 | 16 | 25 | 31 | 19 | 23 | 18 | 25 | 18 | 25 |
| Edentulism | 24 | 21 | 23 | 23 | 20 | 20 | 22 | 20 | 23 | 24 | 24 | 18 |
| Alzheimer's disease | 25 | 28 | 30 | 29 | 28 | 27 | 30 | 28 | 28 | 32 | 29 | 29 |

B. Females

| | Colorado | United States of America | Arizona | California | Idaho | Montana | Nevada | New Mexico | Oregon | Utah | Washington | Wyoming |
|---|---|---|---|---|---|---|---|---|---|---|---|---|
| Low back pain | 1 | 1 | 1 | 1 | 1 | 1 | 1 | 1 | 1 | 1 | 1 | 1 |
| Other musculoskeletal | 2 | 2 | 2 | 2 | 2 | 2 | 3 | 2 | 2 | 3 | 2 | 2 |
| Major depression | 3 | 3 | 3 | 4 | 3 | 3 | 4 | 3 | 3 | 2 | 3 | 3 |
| Migraine | 4 | 4 | 5 | 3 | 5 | 5 | 5 | 5 | 4 | 5 | 4 | 4 |
| Opioid use disorders | 5 | 5 | 4 | 7 | 4 | 4 | 2 | 4 | 5 | 4 | 5 | 5 |
| Anxiety disorders | 6 | 6 | 6 | 5 | 7 | 6 | 6 | 6 | 7 | 6 | 7 | 6 |
| Neck pain | 7 | 7 | 7 | 6 | 6 | 7 | 7 | 7 | 6 | 7 | 6 | 7 |
| Asthma | 8 | 9 | 8 | 8 | 11 | 10 | 8 | 9 | 9 | 10 | 10 | 11 |
| Falls | 9 | 10 | 10 | 11 | 9 | 8 | 12 | 10 | 10 | 9 | 9 | 9 |
| Age-related hearing loss | 10 | 11 | 12 | 10 | 10 | 12 | 11 | 11 | 11 | 11 | 11 | 12 |
| COPD | 11 | 12 | 11 | 12 | 12 | 11 | 10 | 12 | 12 | 12 | 12 | 8 |
| Diabetes type 2 | 12 | 8 | 9 | 9 | 8 | 9 | 9 | 8 | 8 | 8 | 8 | 10 |
| Schizophrenia | 13 | 13 | 13 | 13 | 13 | 13 | 13 | 15 | 13 | 13 | 14 | 14 |
| Alcohol use disorders | 14 | 20 | 17 | 18 | 17 | 15 | 17 | 16 | 15 | 21 | 19 | 15 |
| Osteoarthritis hand | 15 | 14 | 16 | 15 | 15 | 14 | 16 | 13 | 14 | 14 | 13 | 13 |
| Endo/metab/blood/immune | 16 | 15 | 14 | 16 | 14 | 16 | 14 | 14 | 16 | 15 | 16 | 16 |
| Other gynecological | 17 | 16 | 15 | 14 | 16 | 19 | 15 | 17 | 17 | 16 | 15 | 18 |
| Premenstrual syndrome | 18 | 17 | 18 | 17 | 18 | 17 | 18 | 18 | 18 | 17 | 17 | 17 |
| Osteoarthritis knee | 19 | 19 | 21 | 20 | 21 | 21 | 22 | 19 | 21 | 19 | 21 | 21 |
| Neonatal preterm birth | 20 | 21 | 22 | 23 | 25 | 23 | 20 | 22 | 23 | 23 | 22 | 25 |
| Ischemic stroke | 21 | 18 | 20 | 21 | 20 | 18 | 19 | 20 | 19 | 18 | 18 | 19 |
| Viral skin diseases | 22 | 25 | 27 | 25 | 23 | 25 | 29 | 29 | 22 | 20 | 26 | 27 |
| Atopic derm | 23 | 22 | 19 | 19 | 26 | 29 | 21 | 24 | 20 | 26 | 20 | 24 |
| Bipolar disorder | 24 | 26 | 23 | 27 | 19 | 20 | 26 | 25 | 26 | 22 | 25 | 23 |
| Dysthymia | 25 | 24 | 24 | 22 | 24 | 24 | 24 | 23 | 24 | 24 | 23 | 22 |

C. Males

| | Colorado | United States of America | Arizona | California | Idaho | Montana | Nevada | New Mexico | Oregon | Utah | Washington | Wyoming |
|---|---|---|---|---|---|---|---|---|---|---|---|---|
| Low back pain | 1 | 1 | 1 | 1 | 1 | 1 | 1 | 1 | 1 | 1 | 1 | 1 |
| Other musculoskeletal | 2 | 3 | 3 | 2 | 2 | 2 | 3 | 3 | 3 | 3 | 2 | 2 |
| Opioid use disorders | 3 | 2 | 2 | 3 | 3 | 3 | 2 | 2 | 2 | 2 | 3 | 3 |
| Major depression | 4 | 6 | 5 | 6 | 4 | 4 | 5 | 5 | 5 | 4 | 5 | 4 |
| Age-related hearing loss | 5 | 5 | 6 | 5 | 6 | 6 | 6 | 6 | 6 | 6 | 7 | 5 |
| Diabetes type 2 | 6 | 4 | 4 | 4 | 5 | 5 | 4 | 4 | 4 | 5 | 4 | 6 |
| Neck pain | 7 | 7 | 7 | 7 | 7 | 14 | 7 | 13 | 8 | 13 | 6 | 12 |
| Anxiety disorders | 8 | 8 | 9 | 8 | 8 | 7 | 8 | 8 | 14 | 7 | 8 | 7 |
| Falls | 9 | 12 | 13 | 14 | 11 | 11 | 13 | 11 | 9 | 9 | 10 | 10 |
| Migraine | 10 | 9 | 11 | 9 | 9 | 8 | 9 | 9 | 7 | 8 | 9 | 9 |
| Asthma | 11 | 10 | 8 | 10 | 12 | 12 | 11 | 10 | 11 | 11 | 11 | 14 |
| COPD | 12 | 11 | 12 | 12 | 10 | 10 | 10 | 12 | 12 | 10 | 12 | 8 |
| Alcohol use disorders | 13 | 13 | 10 | 11 | 14 | 9 | 12 | 7 | 10 | 14 | 14 | 11 |
| Schizophrenia | 14 | 14 | 14 | 13 | 13 | 13 | 14 | 14 | 13 | 12 | 13 | 13 |
| Other mental disorders | 15 | 15 | 16 | 15 | 16 | 16 | 15 | 15 | 15 | 16 | 15 | 17 |
| Autism spectrum | 16 | 17 | 15 | 16 | 18 | 18 | 17 | 17 | 16 | 15 | 16 | 18 |
| Neonatal preterm birth | 17 | 16 | 17 | 20 | 19 | 19 | 16 | 18 | 20 | 19 | 19 | 21 |
| Other mechanical forces | 18 | 19 | 23 | 18 | 15 | 15 | 18 | 19 | 18 | 18 | 17 | 15 |
| Bipolar disorder | 19 | 22 | 18 | 23 | 17 | 20 | 30 | 24 | 24 | 21 | 24 | 30 |
| Viral skin diseases | 20 | 20 | 26 | 21 | 22 | 24 | 28 | 30 | 22 | 17 | 23 | 28 |
| Osteoarthritis hand | 21 | 24 | 24 | 25 | 21 | 17 | 25 | 16 | 19 | 22 | 21 | 16 |
| Osteoarthritis knee | 22 | 25 | 25 | 24 | 26 | 25 | 26 | 23 | 26 | 23 | 25 | 22 |
| Ischemic stroke | 23 | 18 | 19 | 19 | 20 | 21 | 19 | 20 | 17 | 20 | 18 | 19 |
| Atrial fibrillation | 24 | 21 | 22 | 22 | 23 | 22 | 23 | 28 | 23 | 26 | 22 | 24 |
| Atopic derm | 25 | 23 | 20 | 17 | 28 | 32 | 21 | 26 | 21 | 28 | 20 | 26 |

**Figure S15.** Heat of top ranked years lived with disability (YLD) in Colorado, West States, and the United States, both sexes, Level 4, age standardized in 1990. *Numbers are rankings, with colors indicating the scale from low (blue: below 17th) to high (red: first and second) rankings.

| | **Colorado** | United States of America | Arizona | California | Idaho | Montana | Nevada | New Mexico | Oregon | Utah | Washington | Wyoming |
|---|---|---|---|---|---|---|---|---|---|---|---|---|
| Low back pain | 1 | 1 | 1 | 1 | 1 | 1 | 1 | 1 | 1 | 1 | 1 | 1 |
| Other musculoskeletal | 2 | 2 | 2 | 3 | 2 | 2 | 3 | 4 | 2 | 4 | 2 | 2 |
| Migraine | 3 | 3 | 3 | 2 | 3 | 3 | 2 | 2 | 3 | 3 | 3 | 3 |
| Major depression | 4 | 4 | 4 | 7 | 4 | 4 | 4 | 3 | 4 | 2 | 4 | 4 |
| Anxiety disorders | 5 | 5 | 5 | 4 | 6 | 5 | 5 | 5 | 7 | 5 | 6 | 5 |
| Age-related hearing loss | 6 | 6 | 6 | 5 | 7 | 6 | 6 | 6 | 6 | 6 | 7 | 6 |
| Neck pain | 7 | 7 | 7 | 6 | 5 | 7 | 7 | 11 | 5 | 8 | 5 | 12 |
| Falls | 8 | 11 | 11 | 10 | 10 | 11 | 12 | 9 | 10 | 9 | 8 | 8 |
| Asthma | 9 | 8 | 8 | 8 | 9 | 9 | 9 | 8 | 8 | 10 | 10 | 9 |
| COPD | 10 | 9 | 10 | 9 | 8 | 8 | 8 | 12 | 9 | 11 | 9 | 7 |
| Alcohol use disorders | 11 | 12 | 9 | 11 | 12 | 10 | 10 | 7 | 11 | 13 | 12 | 10 |
| Schizophrenia | 12 | 13 | 13 | 13 | 13 | 13 | 13 | 14 | 13 | 12 | 13 | 13 |
| Diabetes type 2 | 13 | 10 | 12 | 12 | 11 | 12 | 11 | 10 | 12 | 7 | 11 | 11 |
| Endo/metab/blood/immune | 14 | 14 | 15 | 15 | 14 | 14 | 15 | 15 | 15 | 14 | 16 | 14 |
| Osteoarthritis hand | 15 | 17 | 16 | 21 | 17 | 15 | 17 | 16 | 16 | 16 | 15 | 15 |
| Opioid use disorders | 16 | 15 | 14 | 14 | 15 | 17 | 14 | 13 | 14 | 15 | 14 | 19 |
| Other mechanical forces | 17 | 19 | 21 | 17 | 16 | 16 | 19 | 18 | 17 | 21 | 18 | 16 |
| Edentulism | 18 | 18 | 18 | 19 | 20 | 19 | 18 | 17 | 19 | 19 | 19 | 18 |
| Osteoarthritis knee | 19 | 22 | 22 | 22 | 23 | 23 | 23 | 21 | 23 | 20 | 22 | 21 |
| Other gynecological | 20 | 21 | 17 | 20 | 21 | 21 | 20 | 19 | 21 | 23 | 21 | 20 |
| Ischemic stroke | 21 | 16 | 20 | 18 | 19 | 18 | 16 | 20 | 18 | 17 | 17 | 17 |
| Neonatal preterm birth | 22 | 20 | 24 | 25 | 30 | 29 | 22 | 23 | 27 | 27 | 29 | 30 |
| Bipolar disorder | 23 | 27 | 23 | 27 | 18 | 20 | 29 | 26 | 26 | 22 | 26 | 28 |
| Viral skin diseases | 24 | 25 | 29 | 26 | 22 | 25 | 30 | 30 | 22 | 18 | 24 | 27 |
| Atopic derm | 25 | 23 | 19 | 16 | 26 | 30 | 21 | 25 | 20 | 26 | 20 | 25 |

**Figure S16.** Top ranked deaths per 100,000 people attributable to major risk factors in Colorado, both sexes, Level 4, age-standardized, in 2019

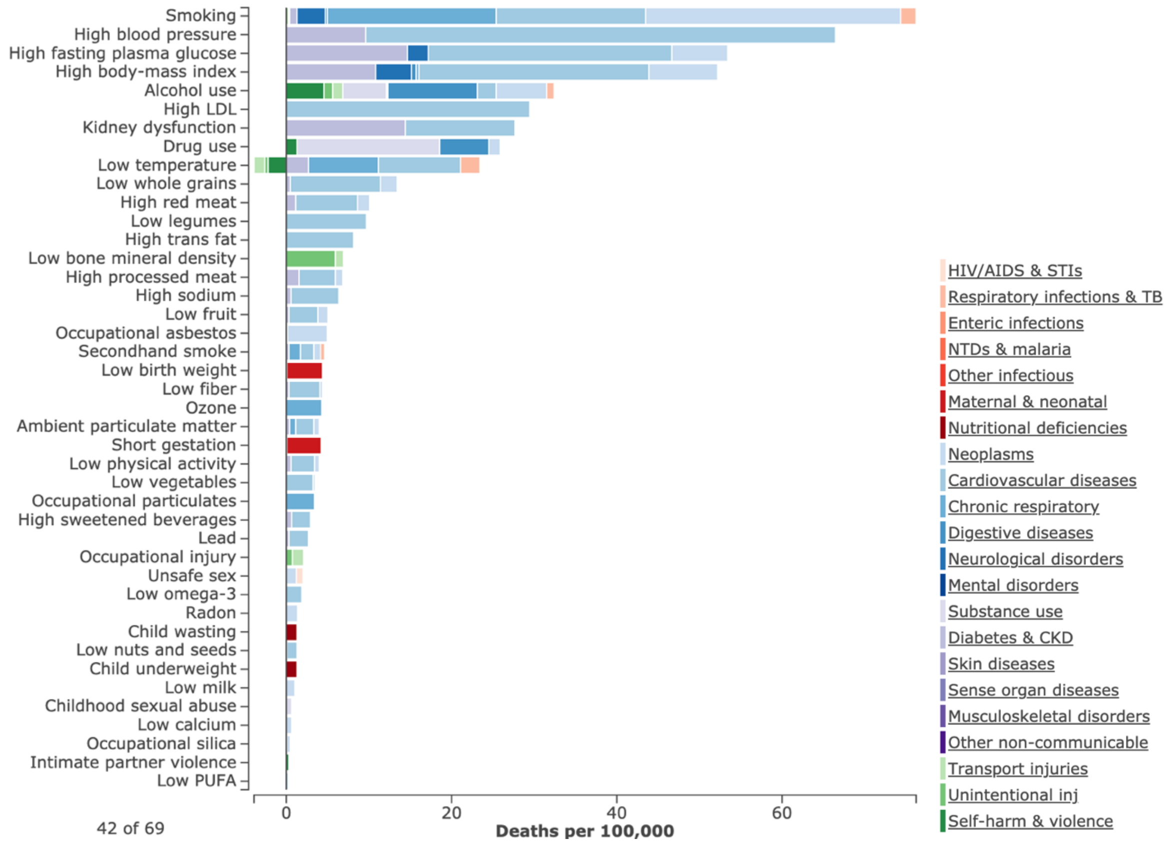

**Figure S17.** Arrow diagram of top risk factors for (A) deaths and (B) disability-adjusted life years (DALYs) per 100,000 people in Colorado, both sexes, Level 4, age-standardized, 1990-2019

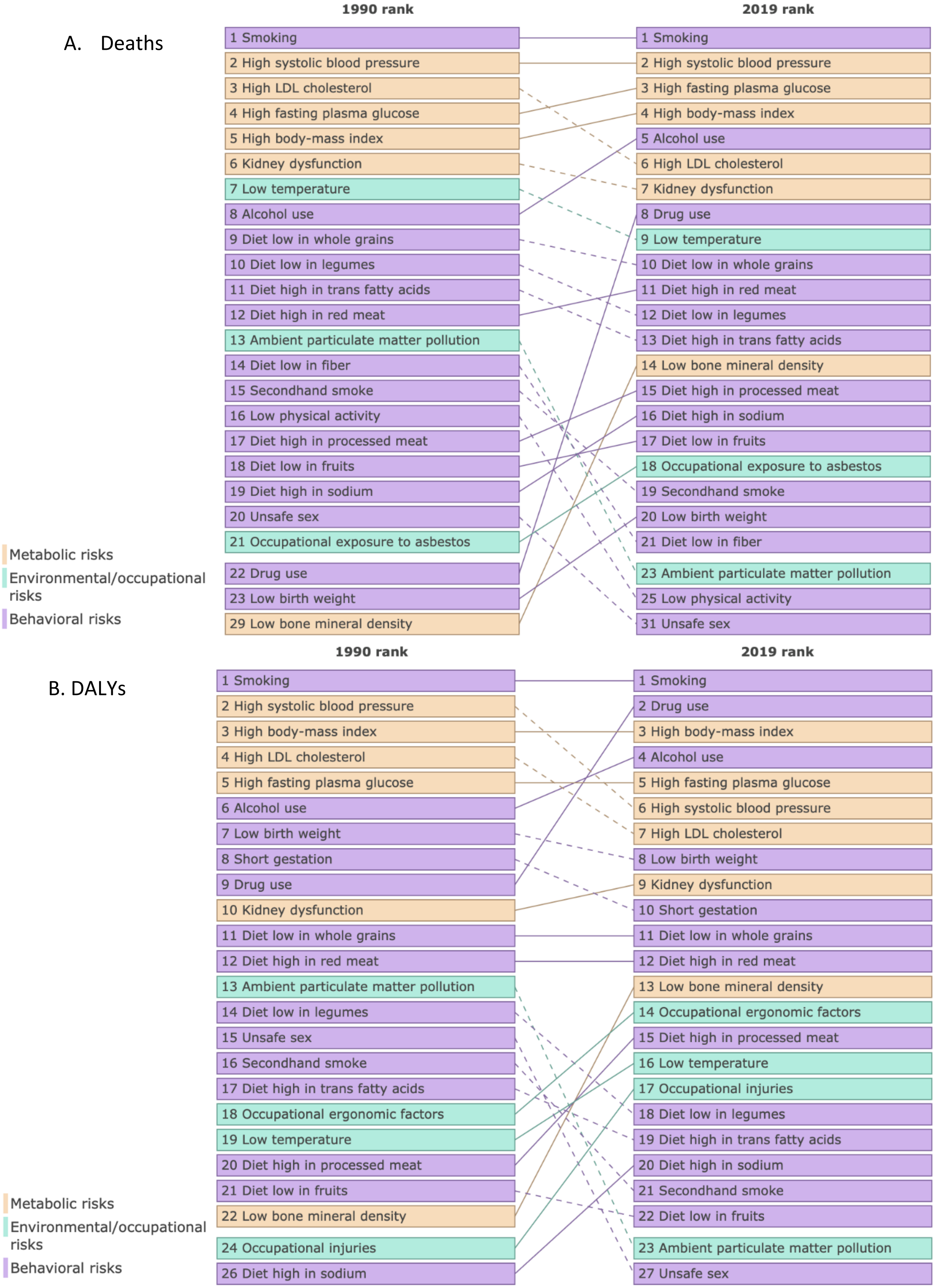

Figure S18. Arrow diagram of top ranked risk factors for deaths in Colorado, in (A) females and (B) males, Level 4, age-standardized, in 2019

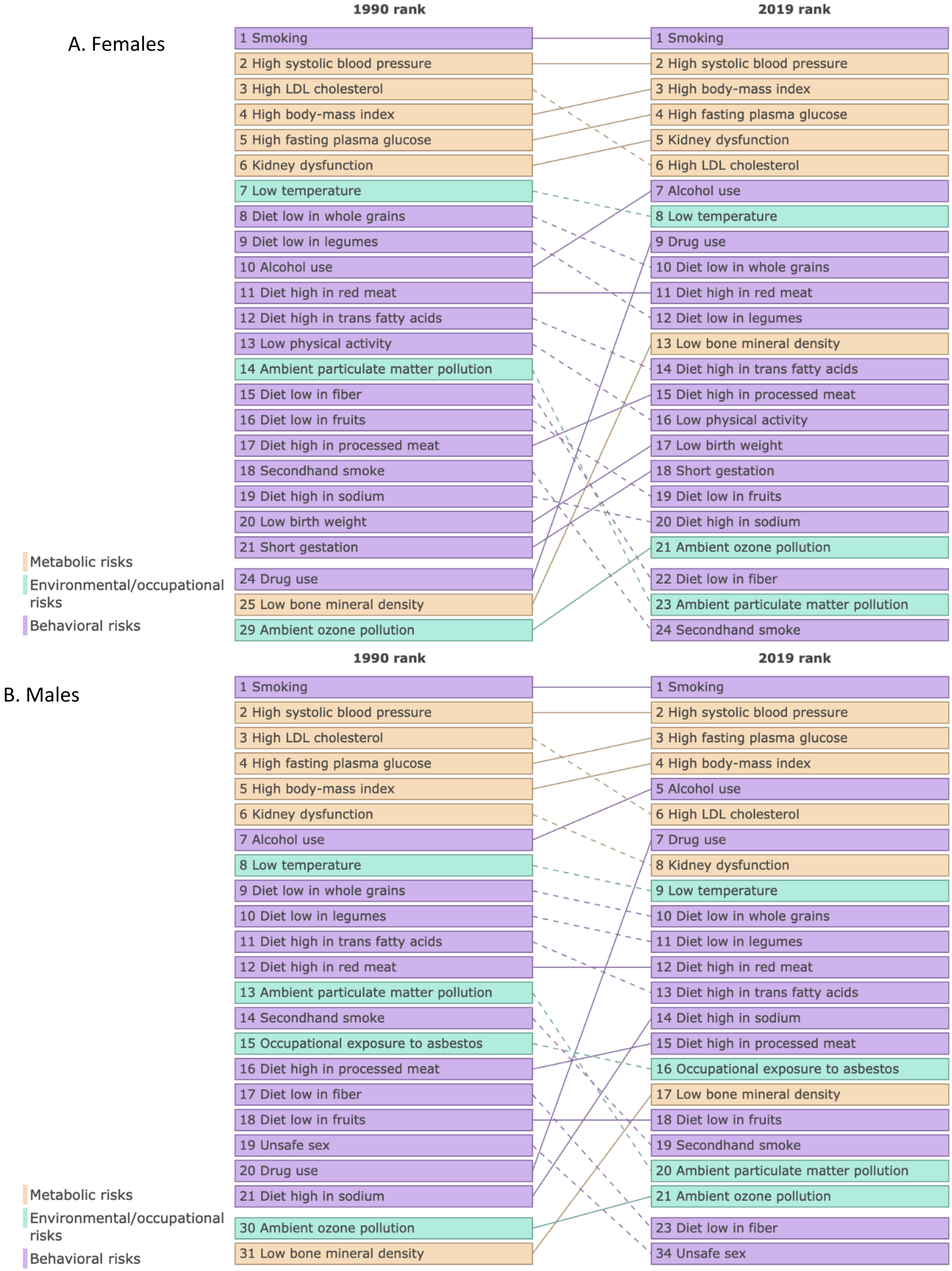

**Figure S19.** Arrow diagram of top ranked risk factors contributing to disability adjusted life years (DALYs) in Colorado, in (A) females and (B) males, Level 4, age-standardized, 2019

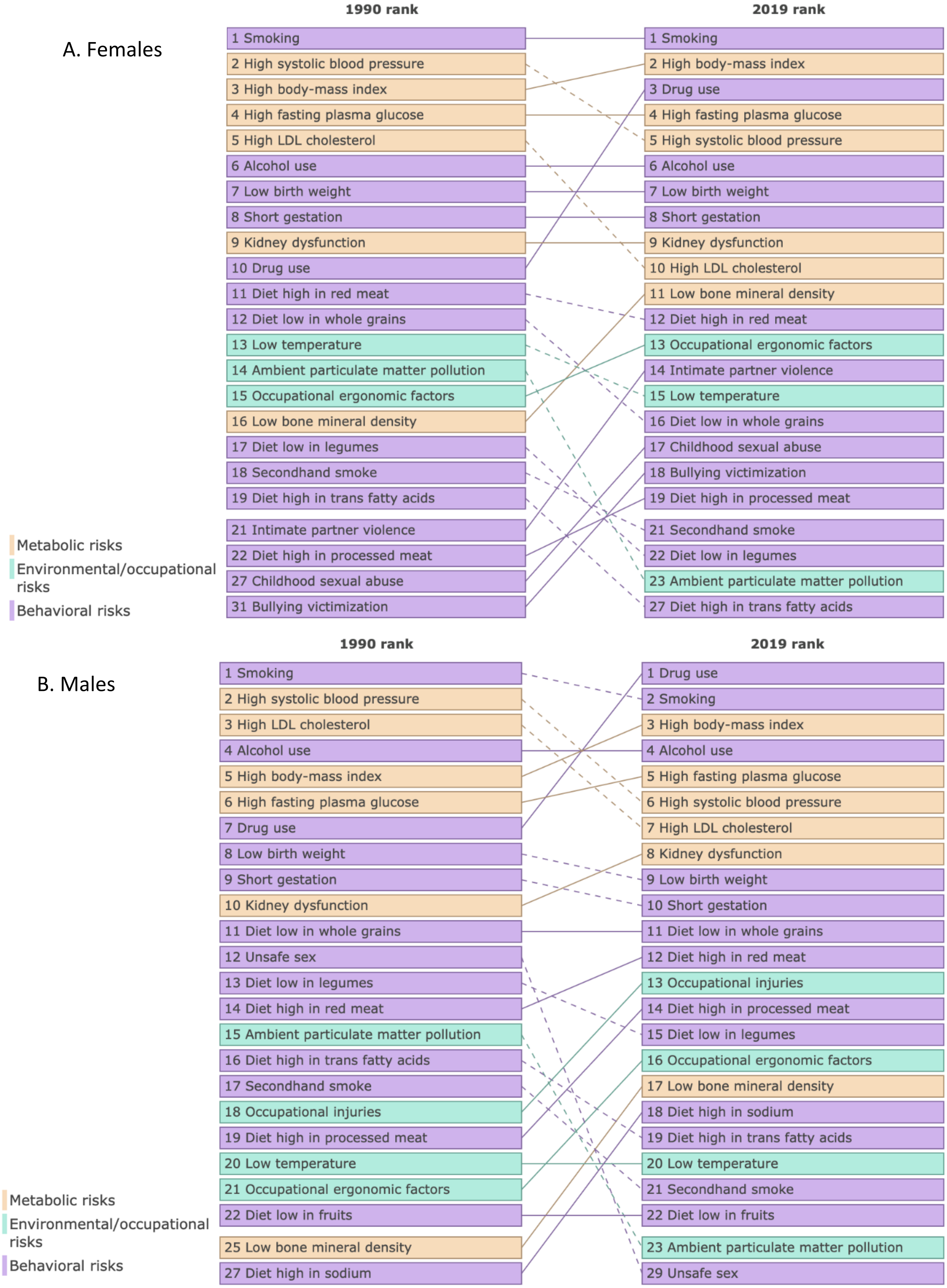

Figure S20. Top ranked risk factors contributing to disability adjusted life years (DALYs) in percent in Colorado by (A) both sexes (B) female (C) males, Level 4, age-standardized, in 2019

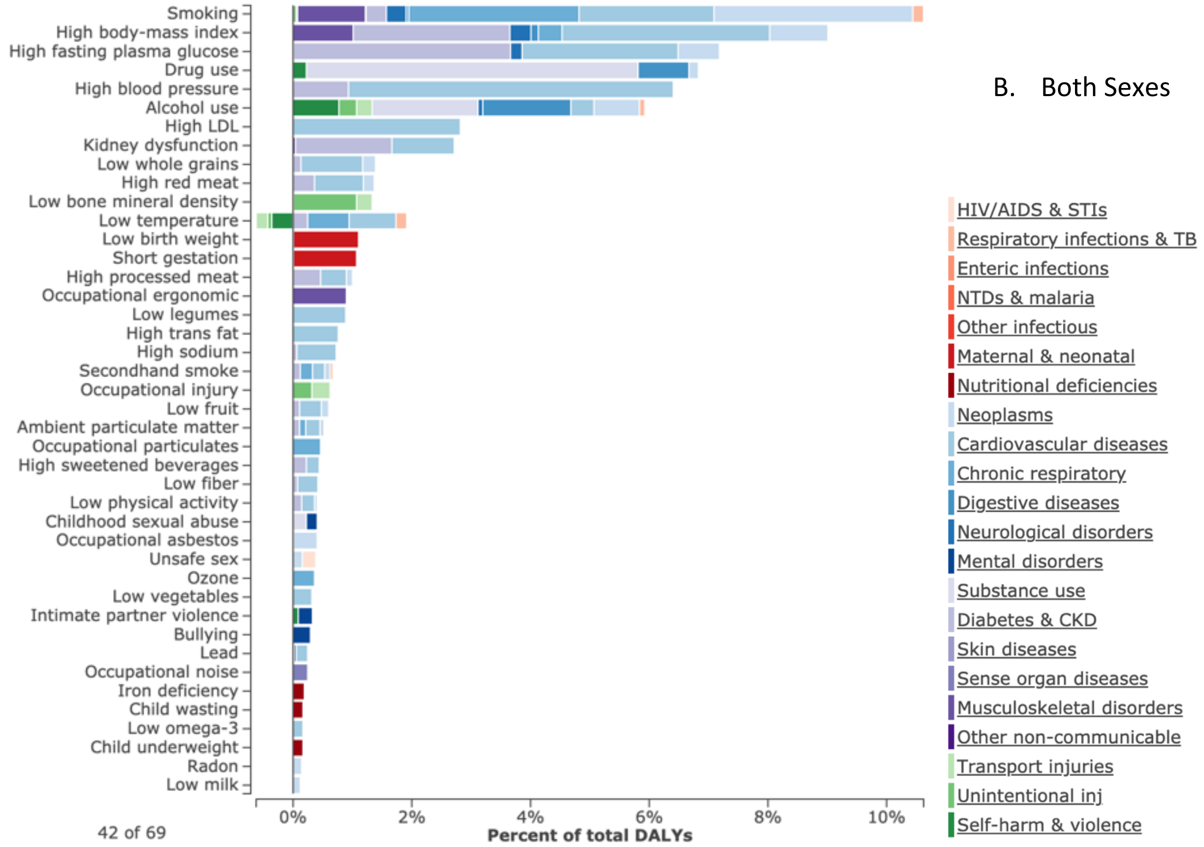


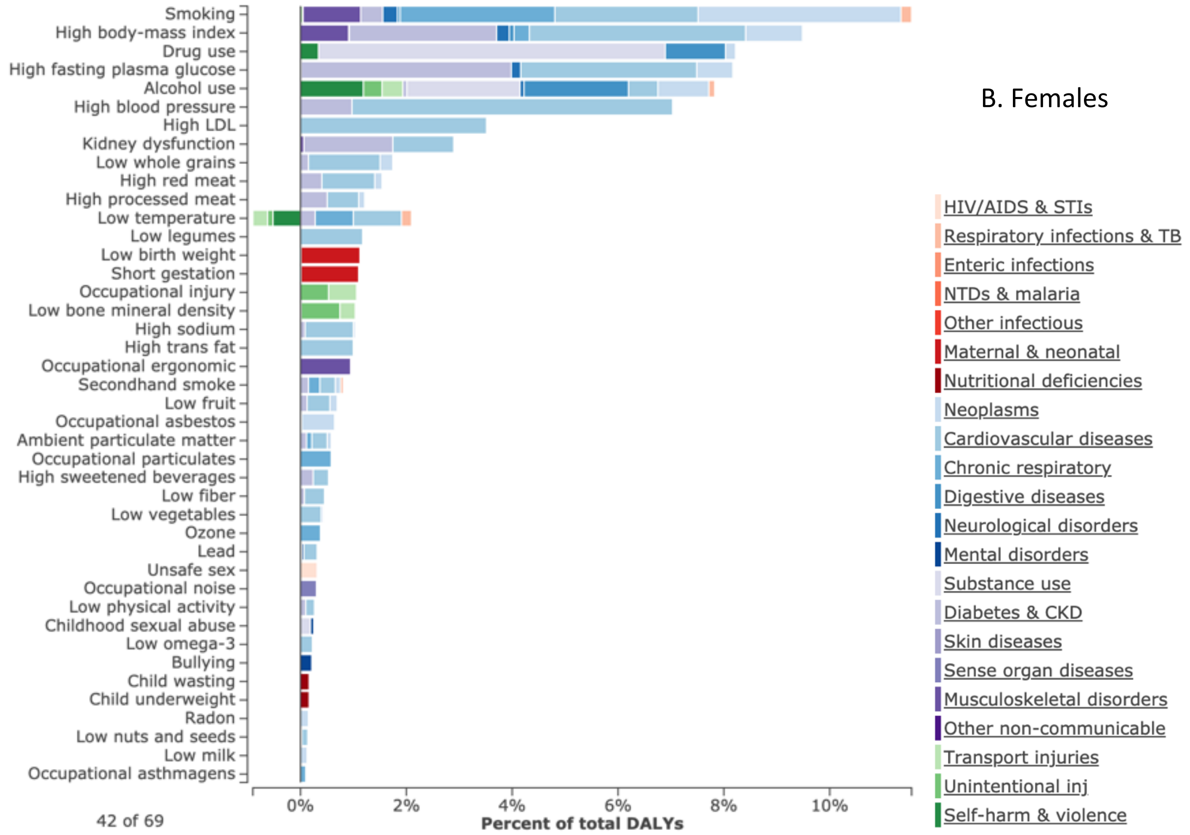

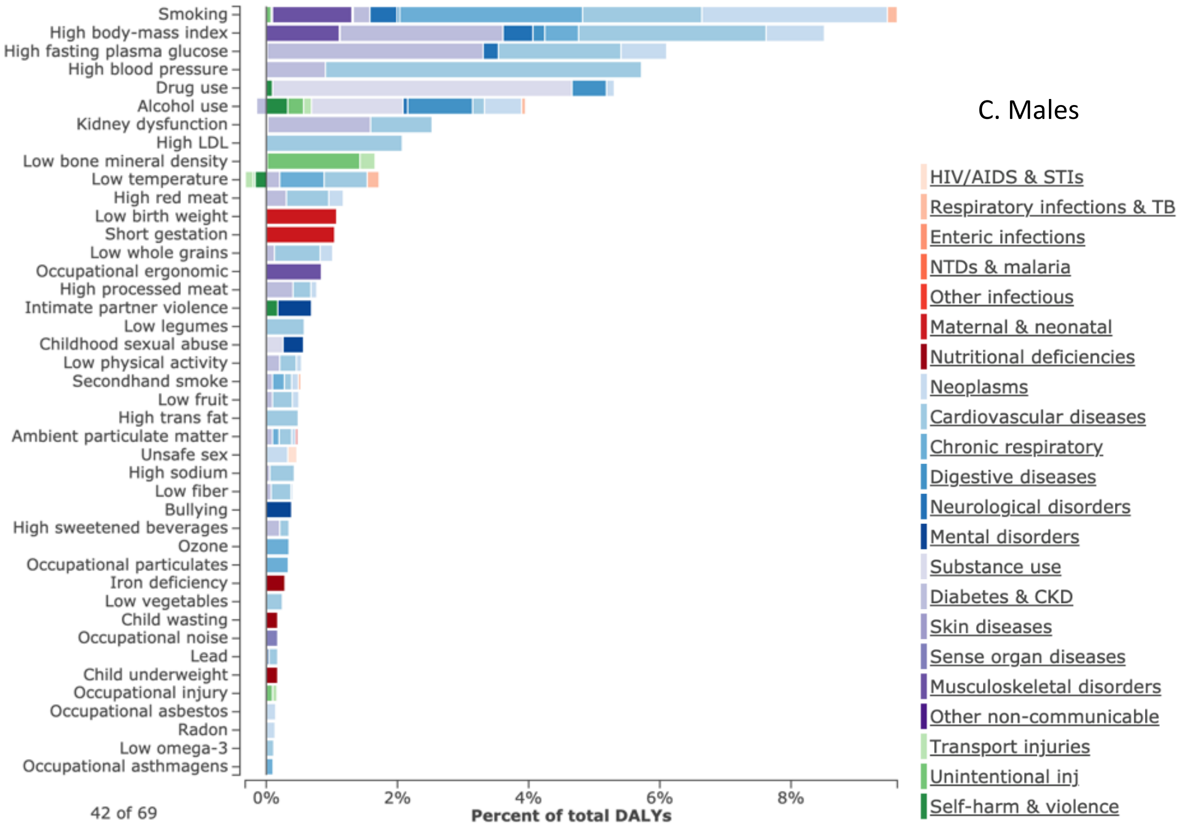
Smoking
High body-mass index
High fasting plasma glucose
High blood pressure
Drug use
Alcohol use
Kidney dysfunction
High LDL
Low bone mineral density
Low temperature
High red meat
Low birth weight
Short gestation
Low whole grains
Occupational ergonomic
High processed meat
Intimate partner violence
Low legumes
Childhood sexual abuse
Low physical activity
Secondhand smoke
Low fruit
High trans fat
Ambient particulate matter
Unsafe sex
High sodium
Low fiber
Bullying
High sweetened beverages
Ozone
Occupational particulates
Iron deficiency
Low vegetables
Child wasting
Occupational noise
Lead
Child underweight
Occupational injury
Occupational asbestos
Radon
Low omega-3
Occupational asthmagens
42 of 69
0%
2%
4%
6%
8%
Percent of total DALYs
C. Males
HIV/AIDS & STIs
Respiratory infections & TB
Enteric infections
NTDs & malaria
Other infectious
Maternal & neonatal
Nutritional deficiencies
Neoplasms
Cardiovascular diseases
Chronic respiratory
Digestive diseases
Neurological disorders
Mental disorders
Substance use
Diabetes & CKD
Skin diseases
Sense organ diseases
Musculoskeletal disorders
Other non-communicable
Transport injuries
Unintentional inj
Self-harm & violence

**Figure S21.** Top ranked risk factors contributing to disability-adjusted life years (DALYs) per 100,000 people, attributable to major risk factors in Colorado in (A) females and (B) males, Level 4, age standardized, in 2019

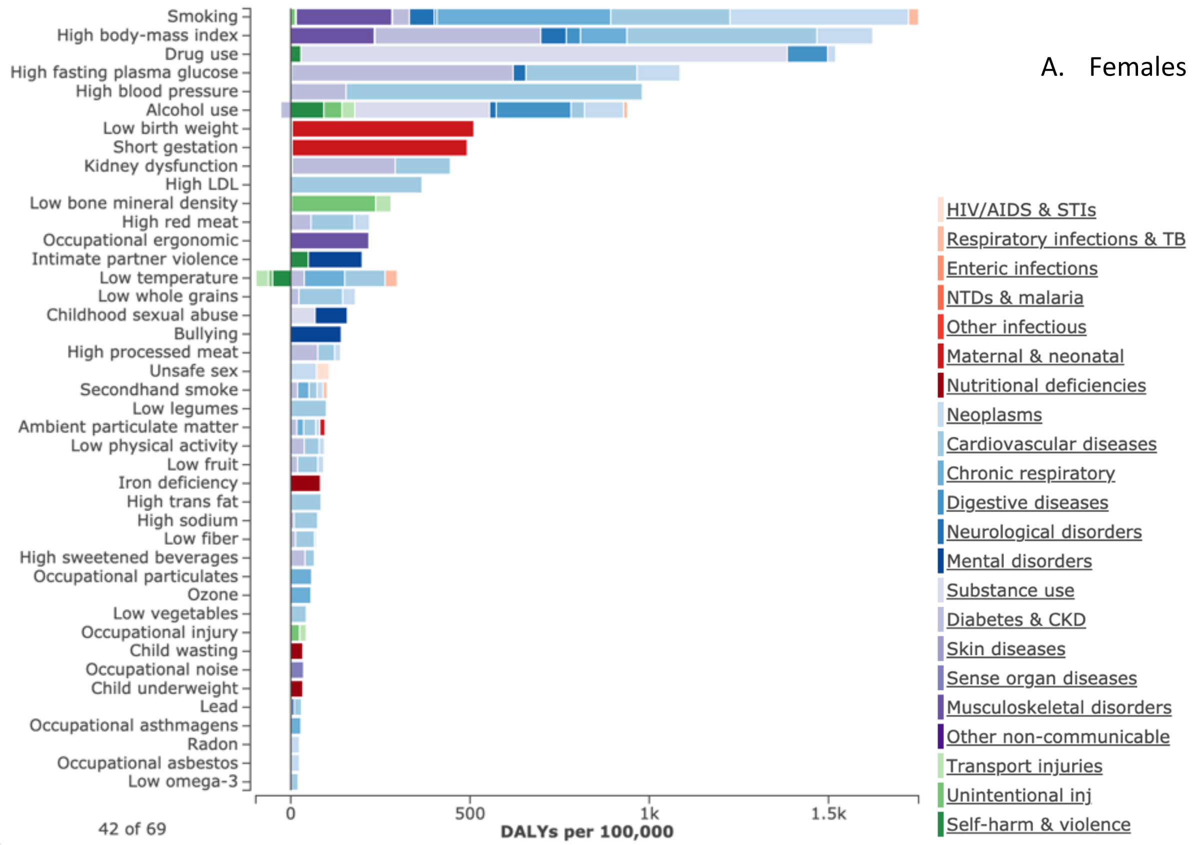


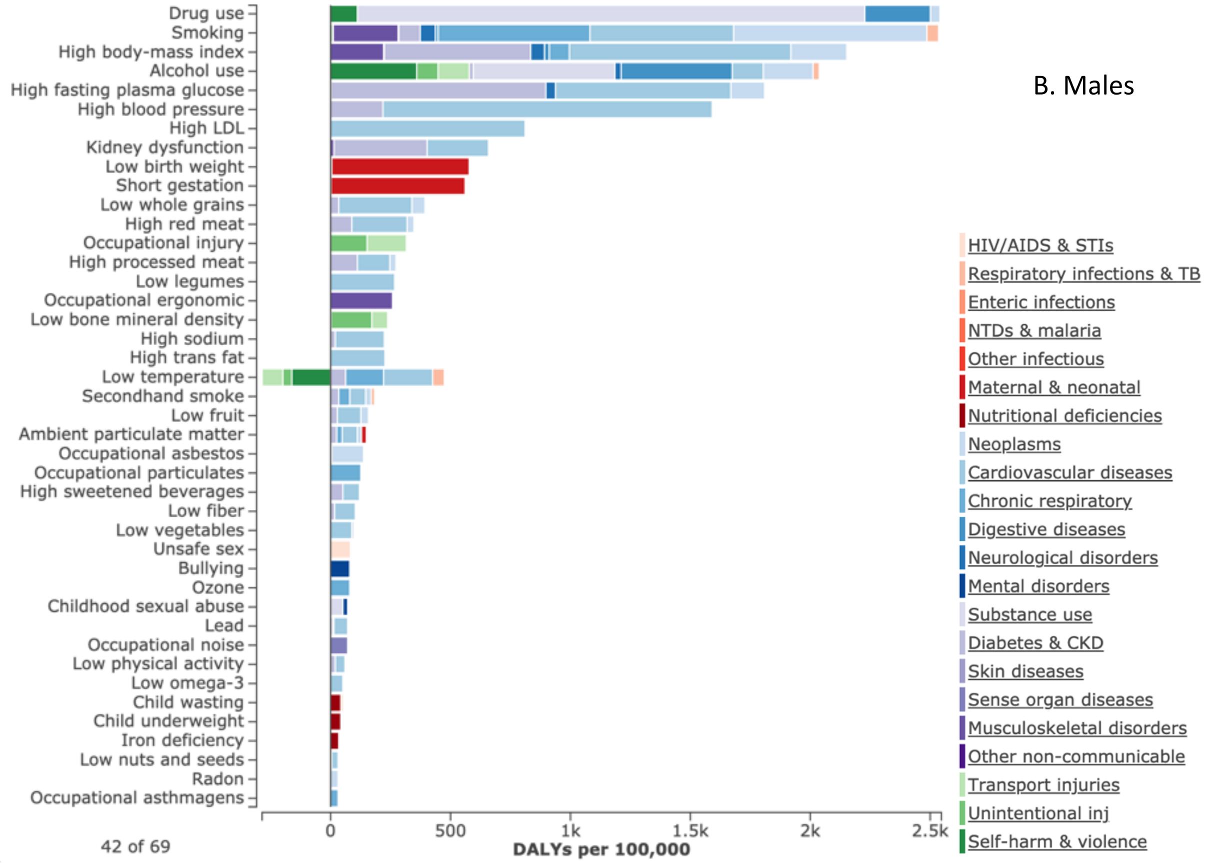

**Table S5.** Colorado age-standardized summary exposure values (SEV), for both sexes, in 1990, 2000, 2010, 2019

| | SEV 1990 | SEV 2000 | SEV 2010 | SEV 2019 |
|---|---|---|---|---|
| **All risk factors** | 20.29 (16.94 – 23.63) | 20.20 (16.83 – 23.48) | 20.37 (16.65 – 24.00) | 20.91 (17.12 – 24.58) |
| **Environmental/occupational risks** | 30.65 (24.55 – 36.87) | 26.64 (21.72 – 31.85) | 23.89 (19.51 – 28.73) | 22.22 (18.34 – 26.69) |
| **Unsafe water, sanitation, and handwashing** | 15.83 (8.61 – 27.05) | 10.52 (6.74 – 15.98) | 7.57 (4.87 – 11.27) | 6.51 (4.44 – 10.03) |
| Unsafe water source | 1.30 (0.72 – 2.19) | 1.23 (0.67 – 2.16) | 0.37 (0.22 – 0.60) | 0.31 (0.19 – 0.48) |
| Unsafe sanitation | 5.43 (2.51 – 9.81) | 4.40 (1.86 – 8.63) | 3.01 (1.27 – 5.73) | 2.33 (1.05 – 4.74) |
| No access to handwashing facility | 3.31 (3.06 – 3.58) | 3.28 (3.03 – 3.55) | 3.09 (2.85 – 3.35) | 3.05 (2.82 – 3.31) |
| **Air pollution** | 4.80 (0.91 – 10.87) | 4.18 (0.96 – 8.45) | 2.80 (1.13 – 4.96) | 2.29 (0.72 – 4.37) |
| Particulate matter pollution | 4.56 (0.75 – 10.55) | 3.91 (0.75 – 8.07) | 2.52 (0.89 – 4.61) | 2.03 (0.52 – 4.05) |
| Ambient particulate matter pollution | 6.63 (1.08 – 15.82) | 5.67 (1.08 – 11.70) | 3.64 (1.35 – 6.35) | 2.94 (0.80 – 5.59) |
| Household air pollution from solid fuels | 0.02 (0.003 – 0.06) | 0.02 (0.003 – 0.06) | 0.01 (0.002 – 0.04) | 0.01 (0.001 – 0.03) |
| Ambient ozone pollution | 66.34 (30.84 – 96.76) | 71.28 (33.77 – 97.80) | 75.28 (39.81 – 98.46) | 72.99 (36.29 – 98.40) |
| **Non-optimal temperature** | 35.46 (31.60 – 40.41) | -- | 34.80 (31.40 – 39.46) | 35.82 (32.48 – 40.33) |
| High temperature | 27.05 (17.89 – 37.63) | -- | 25.20 (16.06 – 36.35) | 25.90 (16.63 – 37.03) |
| Low temperature | 35.95 (31.98 – 40.96) | -- | 35.49 (31.91 – 40.04) | 36.52 (33.01 – 40.94) |
| **Other environmental risks** | 41.72 (20.47 – 63. 35) | 33.55 (14.41 – 55.20) | 26.84 (9.85 – 47.54) | 20.85 (6.55 – 39.70) |
| Residential radon | 23.51 (4.20 – 63.71) | 23.51 (4.20 – 63.71) | 23.51 (4.20 – 63.71) | 23.51 (4.20 – 63.71) |
| Lead exposure | 51.40 (23.60 – 74.48) | 38.78 (17.64 – 62.02) | 28.51 (9.19 – 50.81) | 19.30 (4.87 – 38.45) |
| **Occupational risks** | 2.88 (2.34 – 3.77) | 2.97 (2.42 – 3.92) | 2.83 (2.30 – 3.83) | 2.86 (2.28 – 3.85) |
| **Behavioral risks** | 18.78 (15.34 – 22.62) | 17.96 (14.58 – 21.74) | 17.42 (13.64 – 21.59) | 17.25 (13.19 – 21.62) |
| **Child and maternal malnutrition** | 9.90 (8.26 – 11.62) | 9.84 (8.26 – 11.51) | 10.14 (8.42 – 11.82) | 10.28 (8.43 – 12.12) |
| Suboptimal breastfeeding | 31.88 (31.07 – 32.73) | 31.10 (30.49 – 31.79) | 30.60 (29.75 – 31.48) | 30.39 (29.53 – 31.27) |
| Non-exclusive breastfeeding | 26.19 (21.44 – 30.93) | 28.26 (23.99 – 32.62) | 25.00 (21.42 – 29.15) | 24.96 (20.65 – 29.60) |
| Discontinued breastfeeding | 26.93 (25.99 – 27.88) | 25.91 (24.60 – 27.04) | 25.24 (23.95 – 26.57) | 25.19 (23.93 – 26.38) |
| Child growth failure | 0.39 (0.26 – 0.54) | 0.39 (0.25 – 0.53) | 0.38 (0.25 – 0.52) | 0.38 (0.24 – 0.52) |
| Child underweight | 0.87 (0.49 – 1.30) | 0.88 (0.49 – 1.32) | 0.89 (0.49 – 1.33) | 0.89 (0.49 – 1.33) |
| Child wasting | 0.76 (0.50 – 1.03) | 0.75 (0.49 – 1.02) | 0.74 (0.49 – 1.00) | 0.74 (0.49 – 1.01) |
| Child stunting | 2.12 (1.1 – 3.09) | 2.03 (1.05 – 2.99) | 1.89 (0.98 – 2.77) | 1.86 (0.97 – 2.77) |
| Low birth weight and short gestation | 13.87 (11.93 – 16.16) | 15.11 (13.35 – 17.19) | 15.59 (13.33 – 18.17) | 14.89 (12.70 – 17.44) |
| Short gestation | 20.75 (18.02 – 23.82) | 22.03 (19.87 – 24.45) | 22.39 (19.51 – 25.77) | 20.76 (17.76 – 24.19) |
| Low birth weight | 11 (9.57 – 12.50) | 11.74 (10.81 – 12.80) | 12.03 (10.70 – 13.68) | 11.56 (10.17 – 13.29) |
| Iron deficiency | 11.35 (9.43 – 13.36) | 11.28 (9.48 – 13.23) | 11.62 (9.63 – 13.52) | 11.79 (9.70 – 13.95) |
| Vitamin A deficiency | 1.11 (0.48 – 2.02) | 1.61 (0.73 – 2.97) | 1.25 (0.56 – 2.39) | 0.96 (0.43 – 1.91) |
| Zinc deficiency | 2.11 (0 – 6.79) | 1.21 (0 – 3.85) | 1.21 (0 – 3.54) | 1.11 (0 – 3.53) |
| **Tobacco** | 36.88 (34.72 – 39.36) | 29.98 (28.73 – 31.25) | 23.19 (22.14 – 24.30) | 21.99 (20.47 – 23.68) |
| Smoking | 22.96 (20.64 – 25.40) | 19.61 (17.79 – 21.52) | 16.00 (14.45 – 17.71) | 13.47 (12.01 – 14.99) |
| Chewing tobacco | 2.14 (1.63 – 2.75) | 2.17 (1.68 – 2.81) | 2.11 (1.59 – 2.83) | 2.05 (1.48 – 2.81) |
| Secondhand smoke | 37.50 (35.65 – 39.52) | 29.77 (28.61 – 30.86) | 22.63 (21.69 – 23.69) | 22.76 (21.13 – 24.42) |
| **Alcohol use** | 12.94 (8.12 – 18.65) | 12.73 (7.52 – 18.61) | 13.89 (7.99 – 20.61) | 14.52 (8.43 – 21.55) |
| **Drug use** | 0.48 (0.26 – 0.92) | 0.57 (0.36 – 0.90) | 0.80 (0.55 – 1.18) | 1.10 (0.79 – 1.52) |
| **Dietary risks** | 33.61 (24.07 – 47.59) | 38.28 (26.43 – 52.21) | 40.13 (26.64 – 54.50) | 39.55 (25.95 – 54.40) |
| Diet low in fruits | 52.60 (41.24 – 65.47) | 48.77 (38.37 – 59.59) | 46.53 (34.95 – 59.15) | 45.21 (34.00 – 58.11) |
| Diet low in vegetables | 23.72 (11.08 – 43.48) | 35.01 (20.98 – 54.91) | 38.81 (21.31 – 60.70) | 35.88 (19.62 – 55.86) |

| | | | | |
|---|---|---|---|---|
| Diet low in legumes | 72.63 (34.45 – 98.89) | 74.78 (40.67 – 99.26) | 75.68 (42.22 – 99.56) | 75.53 (43.49 – 99.43) |
| Diet low in whole grains | 77.93 (68.32 – 87.40) | 79.74 (71.23 – 88.12) | 80.43 (71.68 – 88.50) | 80.18 (71.31 – 88.29) |
| Diet low in nuts and seeds | 24.12 (7.14 – 54.38) | 24.40 (8.19 – 55.06) | 23.76 (7.87 – 55.02) | 19.01 (6.30 – 53.18) |
| Diet low in milk | 46.87 (24.12 – 66.65) | 43.91 (21.85 – 63.51) | 41.48 (18.49 – 61.94) | 39.67 (17.70 – 59.80) |
| Diet high in red meat | 85.70 (77.67 – 92.17) | 83.59 (76.11 – 89.70) | 82.38 (72.73 – 89.51) | 82.08 (72.20 – 89.81) |
| Diet high in processed meat | 78.04 (54.74 – 97.57) | 84.20 (66.55 – 98.61) | 86.65 (70.08 – 99.63) | 86.13 (67.76 – 99.61) |
| Diet high in sugar-sweetened beverages | 49.44 (34.32 – 65.76) | 61.46 (48.25 – 74.79) | 66.95 (52.29 – 80.41) | 67.29 (53.27 – 81.05) |
| Diet low in fiber | 38.77 (24.65 – 52.44) | 31.06 (19.31 – 43.36) | 29.03 (17.94 – 41.02) | 24.95 (14.22 – 36.54) |
| Diet low in calcium | 12.35 (5.63 – 22.53) | 8.78 (4.06 – 15.70) | 10.99 (5.21 – 19.66) | 10.00 (4.63 – 18.22) |
| Diet low in seafood omega-3 fatty acids | 81.00 (64.90 – 99.12) | 78.57 (63.53 – 97.49) | 77.04 (60.53 – 96.71) | 76.32 (59.92 – 97.46) |
| Diet low in polyunsaturated fatty acids | 12.90 (4.07 – 33.33) | 8.94 (2.83 – 26.80) | 6.31 (2.03 – 22.10) | 5.85 (1.93 – 21.43) |
| Diet high in trans fatty acids | 87.84 (82.05 – 92.91) | 88.62 (83.41 – 93.29) | 86.23 (79.76 – 92.69) | 85.71 (78.72 – 92.20) |
| Diet high in sodium | 18.53 (4.45 – 41.94) | 33.68 (9.37 – 57.44) | 40.09 (10.42 – 64.35) | 39.49 (10.34 – 64.21) |
| **Intimate partner violence** | 29.03 (16.04 – 37.84) | 28.01 (16.20 – 36.75) | 27.43 (15.23 – 36.06) | 28.41 (15.26 – 37.49) |
| **Childhood sexual abuse and bullying** | 7.70 (5.14 – 11.44) | 10.13 (6.71 – 15.16) | 11.16 (7.20 – 17.00) | 11.13 (7.17 – 17.19) |
| Childhood sexual abuse | 9.79 (7.68 – 12.66) | 11.47 (8.95 – 14.90) | 12.11 (9.47 – 15.73) | 12.28 (9.57 – 16.00) |
| Bullying victimization | 5.08 (2.09 – 10.50) | 7.35 (3.18 – 14.28) | 8.43 (3.36 – 16.75) | 8.31 (3.14 – 17.12) |
| **Unsafe sex** | -- | -- | -- | -- |
| **Low physical activity** | 3.47 (1.64 – 6.90) | 3.00 (1.59 – 5.46) | 3.07 (1.51 – 5.75) | 3.11 (1.47 – 6.04) |
| **Metabolic risks** | 25.82 (20.53 – 31.73) | 30.10 (24.95 – 35.89) | 33.27 (27.31 – 39.41) | 35.89 (29.72 – 42.26) |
| **High fasting plasma glucose** | 9.44 (8.20 – 10.78) | 8.28 (7.20 – 9.38) | 11.58 (10.38 – 12.86) | 13.19 (11.83 – 14.80) |
| **High LDL cholesterol** | 66.61 (63.36 – 69.93) | 39.72 (36.62 – 42.94) | 34.36 (31.06 – 37.65) | 31.36 (27.95 – 34.99) |
| **High systolic blood pressure** | 23.43 (19.27 – 28.06) | 15.96 (14.33 – 17.60) | 13.83 (12.26 – 15.54) | 18.27 (14.75 – 22.34) |
| **High body-mass index** | 25.33 (18.46 – 33.67) | 31.71 (24.19 – 40.40) | 36.71 (28.76 – 45.55) | 39.03 (30.53 – 47.94) |
| **Low bone mineral density** | 14.74 (9.72 – 21.42) | 13.22 (8.40 – 19.55) | 13.49 (8.76 – 19.82) | 15.76 (10.42 – 22.70) |
| **Kidney dysfunction** | 19.98 (14.15 – 26.97) | 20.28 (14.37 – 27.33) | 20.49 (14.51 – 27.67) | 20.95 (14.96 – 28.16) |

Data in parenthesis are 95% uncertainty intervals. SEVs measured 0 to 100 scale, in which 100 is when the entire population is exposed to maximum risk and 0 is when the entire population is at minimum risk. SEVs are shown for all levels of the risk factor hierarchy. SEV = summary exposure values

**Figure S22.** Heat map of top ranked risk factors in Colorado, West States, and the United States in (A) both sexes (B) females and (C) males, Level 4, age standardized, in 2019. *Numbers are rankings, with colors indicating the scale from low (blue: below 17th) to high (red: first and second) rankings.

A. Both Sexes

| | Colorado | United States of America | Arizona | California | Idaho | Montana | Nevada | New Mexico | Oregon | Utah | Washington | Wyoming |
|---|---|---|---|---|---|---|---|---|---|---|---|---|
| Smoking | 1 | 1 | 2 | 2 | 1 | 1 | 1 | 2 | 1 | 4 | 1 | 1 |
| Drug use | 2 | 3 | 1 | 4 | 3 | 2 | 2 | 1 | 2 | 1 | 2 | 2 |
| High body-mass index | 3 | 2 | 3 | 1 | 2 | 3 | 3 | 3 | 3 | 2 | 3 | 3 |
| Alcohol use | 4 | 6 | 6 | 6 | 6 | 5 | 6 | 5 | 5 | 6 | 6 | 5 |
| High fasting plasma glucose | 5 | 4 | 4 | 3 | 4 | 4 | 4 | 4 | 4 | 3 | 4 | 4 |
| High blood pressure | 6 | 5 | 5 | 5 | 5 | 6 | 5 | 6 | 6 | 5 | 5 | 6 |
| High LDL | 7 | 7 | 7 | 7 | 7 | 7 | 7 | 7 | 7 | 8 | 7 | 7 |
| Low birth weight | 8 | 9 | 9 | 9 | 9 | 9 | 9 | 9 | 9 | 9 | 9 | 9 |
| Kidney dysfunction | 9 | 8 | 8 | 8 | 8 | 8 | 8 | 8 | 8 | 7 | 8 | 8 |
| Short gestation | 10 | 10 | 10 | 10 | 10 | 10 | 10 | 10 | 10 | 10 | 10 | 10 |
| Low whole grains | 11 | 11 | 11 | 11 | 11 | 12 | 11 | 11 | 11 | 11 | 11 | 11 |
| High red meat | 12 | 12 | 12 | 12 | 12 | 11 | 13 | 12 | 12 | 12 | 12 | 13 |
| Low bone mineral density | 13 | 19 | 16 | 19 | 14 | 14 | 21 | 15 | 13 | 15 | 15 | 15 |
| Occupational ergonomic | 14 | 17 | 14 | 16 | 13 | 13 | 16 | 16 | 14 | 14 | 13 | 12 |
| High processed meat | 15 | 14 | 13 | 14 | 15 | 15 | 15 | 13 | 16 | 16 | 14 | 14 |
| Low temperature | 16 | 13 | 15 | 15 | 17 | 26 | 12 | 14 | 15 | 13 | 16 | 22 |
| Occupational injury | 17 | 23 | 22 | 18 | 18 | 21 | 22 | 21 | 18 | 18 | 17 | 19 |
| Low legumes | 18 | 15 | 17 | 17 | 16 | 16 | 14 | 17 | 17 | 19 | 18 | 16 |
| High trans fat | 19 | 18 | 18 | 20 | 20 | 17 | 18 | 18 | 21 | 21 | 19 | 17 |
| High sodium | 20 | 21 | 21 | 21 | 23 | 19 | 19 | 20 | 22 | 20 | 20 | 20 |
| Secondhand smoke | 21 | 20 | 19 | 22 | 22 | 18 | 17 | 19 | 19 | 23 | 22 | 18 |
| Low fruit | 22 | 22 | 23 | 24 | 19 | 20 | 23 | 22 | 23 | 22 | 21 | 21 |
| Ambient particulate matter | 23 | 16 | 20 | 13 | 21 | 22 | 20 | 30 | 20 | 17 | 23 | 35 |
| Childhood sexual abuse | 24 | 33 | 27 | 28 | 28 | 24 | 29 | 23 | 25 | 28 | 25 | 26 |
| Bullying | 25 | 29 | 30 | 27 | 26 | 27 | 32 | 28 | 29 | 25 | 26 | 28 |

B. Females

| | Colorado | United States of America | Arizona | California | Idaho | Montana | Nevada | New Mexico | Oregon | Utah | Washington | Wyoming |
|---|---|---|---|---|---|---|---|---|---|---|---|---|
| Smoking | 1 | 2 | 1 | 2 | 1 | 1 | 1 | 3 | 1 | 4 | 1 | 1 |
| High body-mass index | 2 | 1 | 2 | 1 | 2 | 2 | 2 | 2 | 2 | 1 | 2 | 2 |
| Drug use | 3 | 4 | 3 | 4 | 3 | 3 | 3 | 1 | 3 | 2 | 3 | 3 |
| High fasting plasma glucose | 4 | 3 | 4 | 3 | 4 | 4 | 4 | 4 | 4 | 3 | 4 | 4 |
| High blood pressure | 5 | 5 | 5 | 5 | 5 | 5 | 5 | 5 | 5 | 5 | 5 | 5 |
| Alcohol use | 6 | 6 | 6 | 6 | 6 | 6 | 6 | 6 | 6 | 6 | 6 | 6 |
| Low birth weight | 7 | 8 | 8 | 8 | 8 | 8 | 9 | 8 | 8 | 8 | 8 | 9 |
| Short gestation | 8 | 9 | 9 | 9 | 9 | 9 | 10 | 9 | 9 | 9 | 9 | 10 |
| Kidney dysfunction | 9 | 7 | 7 | 7 | 7 | 7 | 7 | 7 | 7 | 7 | 7 | 7 |
| High LDL | 10 | 10 | 10 | 10 | 10 | 10 | 8 | 10 | 10 | 10 | 10 | 8 |
| Low bone mineral density | 11 | 14 | 11 | 15 | 12 | 12 | 16 | 11 | 11 | 11 | 11 | 12 |
| High red meat | 12 | 11 | 13 | 11 | 11 | 11 | 13 | 12 | 13 | 13 | 12 | 13 |
| Occupational ergonomic | 13 | 15 | 15 | 16 | 14 | 13 | 15 | 16 | 14 | 15 | 15 | 11 |
| Intimate partner violence | 14 | 18 | 14 | 23 | 16 | 15 | 14 | 15 | 15 | 14 | 14 | 14 |
| Low temperature | 15 | 13 | 16 | 14 | 13 | 16 | 11 | 13 | 12 | 12 | 13 | 16 |
| Low whole grains | 16 | 12 | 12 | 13 | 15 | 14 | 12 | 14 | 16 | 16 | 16 | 15 |
| Childhood sexual abuse | 17 | 26 | 18 | 21 | 20 | 18 | 21 | 18 | 18 | 19 | 17 | 18 |
| Bullying | 18 | 25 | 22 | 20 | 21 | 20 | 26 | 20 | 19 | 20 | 19 | 20 |
| High processed meat | 19 | 16 | 17 | 17 | 17 | 17 | 17 | 17 | 17 | 17 | 18 | 17 |
| Unsafe sex | 20 | 19 | 24 | 18 | 24 | 24 | 23 | 19 | 22 | 24 | 24 | 23 |
| Secondhand smoke | 21 | 22 | 23 | 22 | 22 | 19 | 18 | 23 | 21 | 26 | 22 | 19 |
| Low legumes | 22 | 20 | 20 | 19 | 23 | 22 | 20 | 22 | 25 | 23 | 25 | 21 |
| Ambient particulate matter | 23 | 17 | 19 | 12 | 19 | 23 | 19 | 30 | 20 | 18 | 23 | 33 |
| Low physical activity | 24 | 21 | 21 | 25 | 25 | 25 | 22 | 21 | 24 | 21 | 21 | 24 |
| Low fruit | 25 | 23 | 25 | 26 | 18 | 21 | 24 | 24 | 23 | 22 | 20 | 22 |

C. Males

| | Colorado | United States of America | Arizona | California | Idaho | Montana | Nevada | New Mexico | Oregon | Utah | Washington | Wyoming |
|---|---|---|---|---|---|---|---|---|---|---|---|---|
| Drug use | 1 | 3 | 1 | 4 | 3 | 2 | 2 | 1 | 2 | 1 | 1 | 2 |
| Smoking | 2 | 1 | 2 | 2 | 1 | 1 | 1 | 2 | 1 | 4 | 2 | 1 |
| High body-mass index | 3 | 2 | 3 | 1 | 2 | 3 | 3 | 4 | 3 | 2 | 3 | 3 |
| Alcohol use | 4 | 6 | 5 | 5 | 6 | 4 | 6 | 3 | 5 | 6 | 5 | 4 |
| High fasting plasma glucose | 5 | 4 | 4 | 3 | 4 | 5 | 5 | 5 | 4 | 3 | 4 | 5 |
| High blood pressure | 6 | 5 | 6 | 6 | 5 | 6 | 4 | 6 | 6 | 5 | 6 | 6 |
| High LDL | 7 | 7 | 7 | 7 | 7 | 7 | 7 | 7 | 7 | 7 | 7 | 7 |
| Kidney dysfunction | 8 | 8 | 8 | 8 | 8 | 8 | 8 | 8 | 8 | 8 | 8 | 8 |
| Low birth weight | 9 | 9 | 9 | 9 | 9 | 9 | 10 | 9 | 9 | 9 | 9 | 9 |
| Short gestation | 10 | 10 | 10 | 10 | 10 | 10 | 11 | 10 | 10 | 10 | 10 | 10 |
| Low whole grains | 11 | 11 | 11 | 11 | 11 | 11 | 9 | 11 | 11 | 11 | 11 | 11 |
| High red meat | 12 | 12 | 12 | 12 | 12 | 12 | 14 | 12 | 12 | 13 | 13 | 13 |
| Occupational injury | 13 | 16 | 15 | 14 | 13 | 15 | 17 | 16 | 13 | 12 | 12 | 15 |
| High processed meat | 14 | 14 | 14 | 15 | 15 | 14 | 15 | 13 | 14 | 15 | 14 | 16 |
| Low legumes | 15 | 13 | 13 | 16 | 14 | 13 | 13 | 14 | 15 | 18 | 15 | 14 |
| Occupational ergonomic | 16 | 20 | 17 | 19 | 16 | 16 | 20 | 18 | 16 | 14 | 16 | 12 |
| Low bone mineral density | 17 | 24 | 22 | 22 | 20 | 19 | 24 | 20 | 21 | 21 | 21 | 20 |
| High sodium | 18 | 17 | 19 | 18 | 18 | 18 | 16 | 19 | 18 | 19 | 19 | 18 |
| High trans fat | 19 | 18 | 16 | 20 | 17 | 17 | 18 | 17 | 19 | 20 | 18 | 17 |
| Low temperature | 20 | 15 | 18 | 17 | 25 | 33 | 12 | 15 | 17 | 16 | 17 | 27 |
| Secondhand smoke | 21 | 21 | 20 | 21 | 21 | 20 | 19 | 21 | 22 | 23 | 22 | 19 |
| Low fruit | 22 | 22 | 23 | 24 | 19 | 21 | 22 | 22 | 24 | 22 | 23 | 22 |
| Ambient particulate matter | 23 | 19 | 21 | 13 | 22 | 23 | 21 | 27 | 23 | 17 | 24 | 30 |
| Occupational asbestos | 24 | 23 | 24 | 26 | 24 | 22 | 23 | 24 | 20 | 25 | 20 | 21 |
| Occupational particulates | 25 | 29 | 28 | 29 | 27 | 26 | 29 | 28 | 27 | 27 | 28 | 23 |

**Figure S23.** Heat map of top ranked risk factors in Colorado, West States, and the United States, both sexes, Level 4, age standardized, in 1990. *Numbers are rankings, with colors indicating the scale from low (blue: below 17th) to high (red: first and second) rankings.

| | **Colorado** | United States of America | Arizona | California | Idaho | Montana | Nevada | New Mexico | Oregon | Utah | Washington | Wyoming |
|---|---|---|---|---|---|---|---|---|---|---|---|---|
| Smoking | 1 | 1 | 1 | 1 | 1 | 1 | 1 | 1 | 1 | 1 | 1 | 1 |
| High blood pressure | 2 | 2 | 2 | 2 | 2 | 2 | 2 | 2 | 2 | 2 | 2 | 2 |
| High body-mass index | 3 | 3 | 3 | 3 | 3 | 3 | 3 | 4 | 3 | 3 | 3 | 3 |
| High LDL | 4 | 4 | 4 | 4 | 4 | 4 | 4 | 6 | 4 | 5 | 4 | 4 |
| High fasting plasma glucose | 5 | 5 | 5 | 5 | 5 | 5 | 5 | 3 | 5 | 4 | 5 | 5 |
| Alcohol use | 6 | 6 | 6 | 6 | 6 | 6 | 6 | 5 | 6 | 6 | 6 | 6 |
| Low birth weight | 7 | 7 | 8 | 9 | 7 | 8 | 10 | 8 | 9 | 10 | 9 | 7 |
| Short gestation | 8 | 8 | 9 | 10 | 8 | 10 | 12 | 9 | 10 | 11 | 10 | 9 |
| Drug use | 9 | 11 | 7 | 7 | 10 | 9 | 8 | 7 | 7 | 8 | 7 | 11 |
| Kidney dysfunction | 10 | 9 | 11 | 12 | 9 | 7 | 7 | 10 | 8 | 7 | 8 | 8 |
| Low whole grains | 11 | 12 | 12 | 13 | 11 | 11 | 9 | 11 | 11 | 9 | 11 | 10 |
| High red meat | 12 | 13 | 14 | 14 | 12 | 12 | 16 | 12 | 13 | 13 | 12 | 12 |
| Ambient particulate matter | 13 | 10 | 10 | 8 | 17 | 14 | 11 | 17 | 16 | 12 | 16 | 22 |
| Low legumes | 14 | 14 | 13 | 15 | 13 | 13 | 14 | 13 | 12 | 14 | 13 | 13 |
| Unsafe sex | 15 | 15 | 17 | 11 | 27 | 27 | 19 | 22 | 22 | 27 | 18 | 30 |
| Secondhand smoke | 16 | 17 | 15 | 18 | 15 | 15 | 13 | 14 | 15 | 20 | 15 | 15 |
| High trans fat | 17 | 16 | 16 | 17 | 14 | 16 | 17 | 16 | 14 | 16 | 14 | 14 |
| Occupational ergonomic | 18 | 22 | 19 | 20 | 16 | 17 | 22 | 18 | 20 | 17 | 21 | 16 |
| Low temperature | 19 | 18 | 18 | 16 | 22 | 26 | 15 | 15 | 17 | 15 | 17 | 24 |
| High processed meat | 20 | 19 | 20 | 19 | 19 | 18 | 18 | 19 | 18 | 18 | 19 | 17 |
| Low fruit | 21 | 20 | 22 | 21 | 18 | 20 | 21 | 21 | 19 | 22 | 20 | 19 |
| Low bone mineral density | 22 | 26 | 23 | 26 | 21 | 21 | 28 | 23 | 24 | 23 | 24 | 20 |
| Low fiber | 23 | 21 | 21 | 22 | 20 | 19 | 20 | 20 | 21 | 19 | 22 | 18 |
| Occupational injury | 24 | 24 | 24 | 23 | 23 | 22 | 26 | 24 | 25 | 21 | 26 | 21 |
| Low physical activity | 25 | 23 | 25 | 27 | 24 | 24 | 23 | 25 | 26 | 24 | 25 | 25 |

Figure S24. Arrow diagram of top risk factors for years of life lived with disability (YLDs) per 100,000 people, in Colorado in (A) both, (B) females, (C) males, Level 4, 1990 - 2019

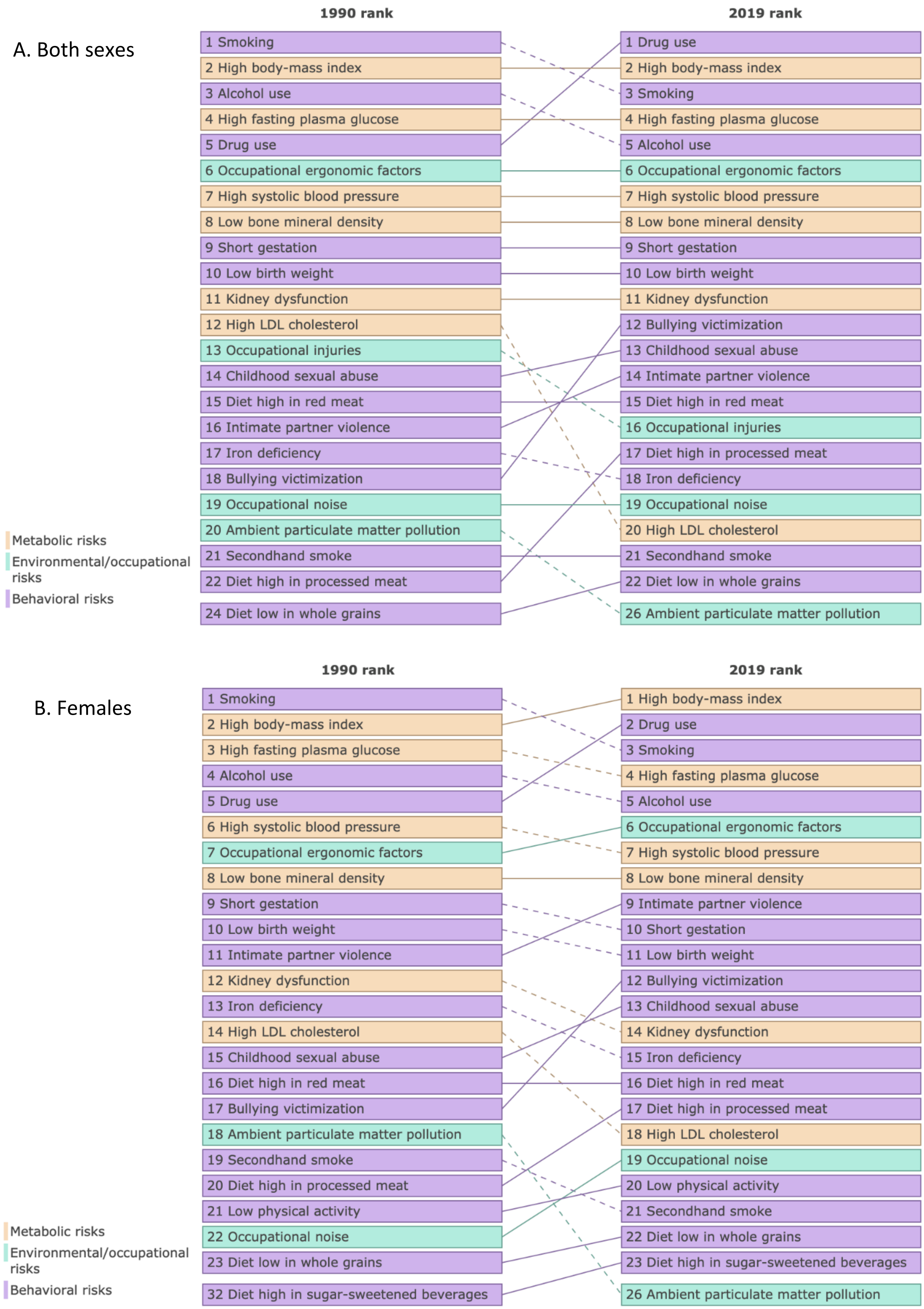

C. Males

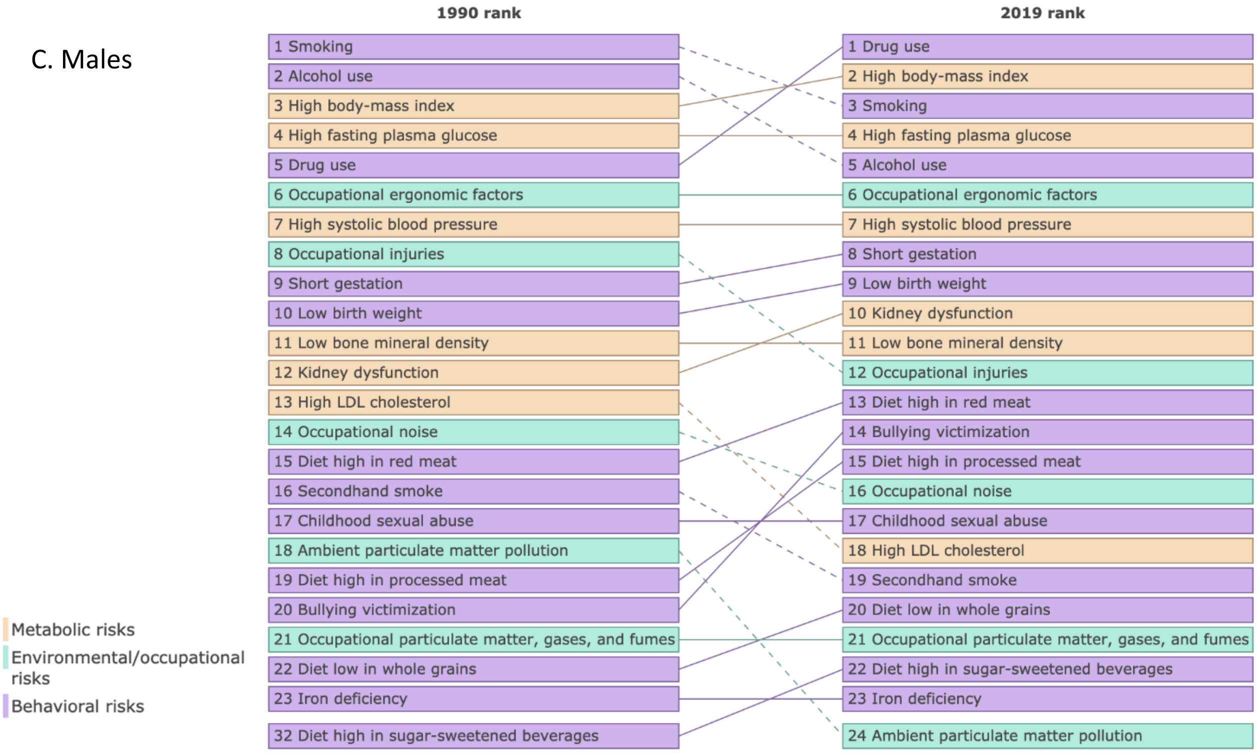

1990 rank
2019 rank
1 Smoking
2 Alcohol use
3 High body-mass index
4 High fasting plasma glucose
5 Drug use
6 Occupational ergonomic factors
7 High systolic blood pressure
8 Occupational injuries
9 Short gestation
10 Low birth weight
11 Low bone mineral density
12 Kidney dysfunction
13 High LDL cholesterol
14 Occupational noise
15 Diet high in red meat
16 Secondhand smoke
17 Childhood sexual abuse
18 Ambient particulate matter pollution
19 Diet high in processed meat
20 Bullying victimization
21 Occupational particulate matter, gases, and fumes
22 Diet low in whole grains
23 Iron deficiency
32 Diet high in sugar-sweetened beverages
1 Drug use
2 High body-mass index
3 Smoking
4 High fasting plasma glucose
5 Alcohol use
6 Occupational ergonomic factors
7 High systolic blood pressure
8 Short gestation
9 Low birth weight
10 Kidney dysfunction
11 Low bone mineral density
12 Occupational injuries
13 Diet high in red meat
14 Bullying victimization
15 Diet high in processed meat
16 Occupational noise
17 Childhood sexual abuse
18 High LDL cholesterol
19 Secondhand smoke
20 Diet low in whole grains
21 Occupational particulate matter, gases, and fumes
22 Diet high in sugar-sweetened beverages
23 Iron deficiency
24 Ambient particulate matter pollution
Metabolic risks
Environmental/occupational risks
Behavioral risks

Figure S25. Arrow diagram of top risk factors for years of life lost (YLLs) per 100,000 people in Colorado in (A) both sexes, (B) females, and (C) males, Level 4, 1990 – 2019

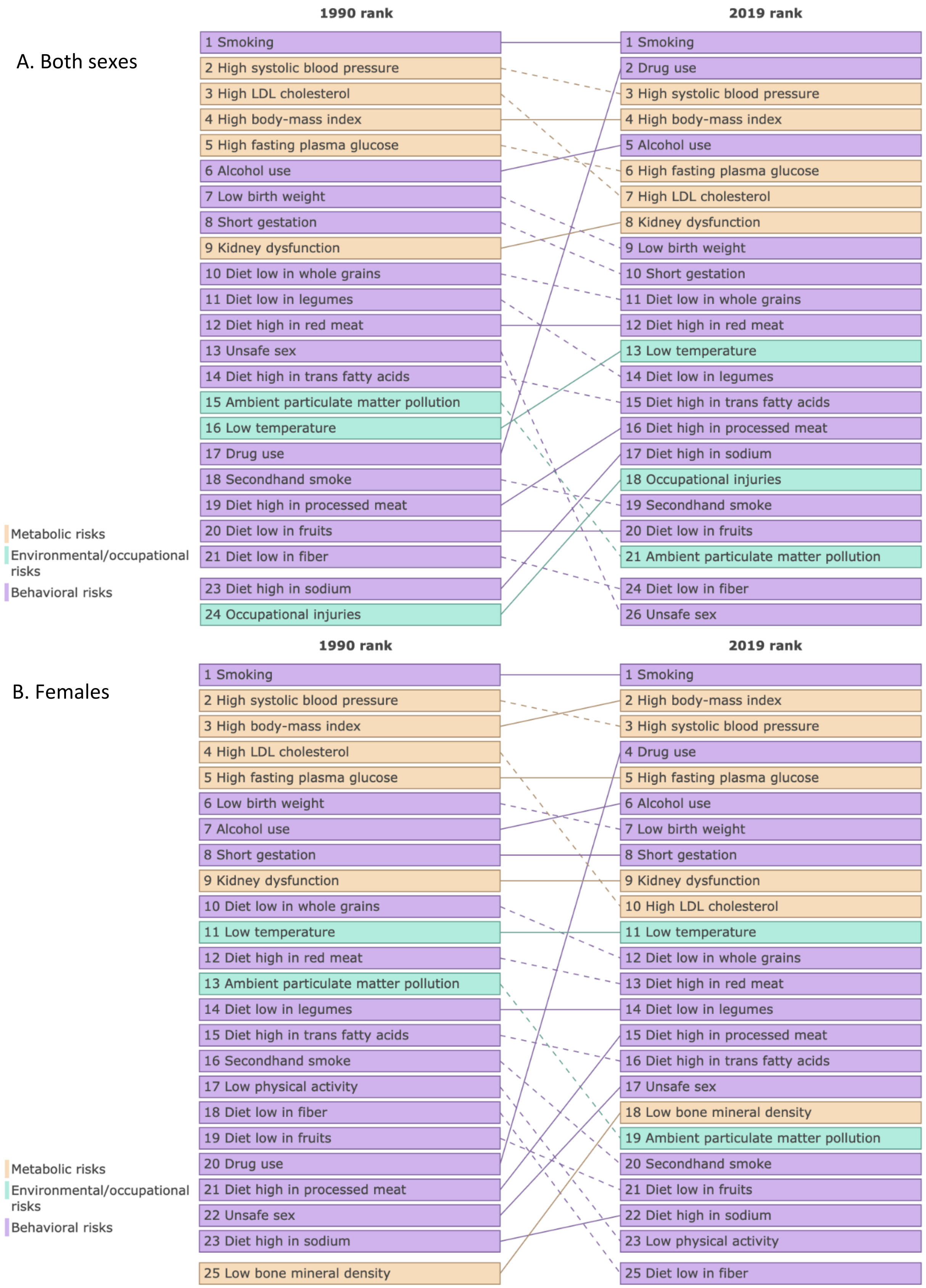

C. Males

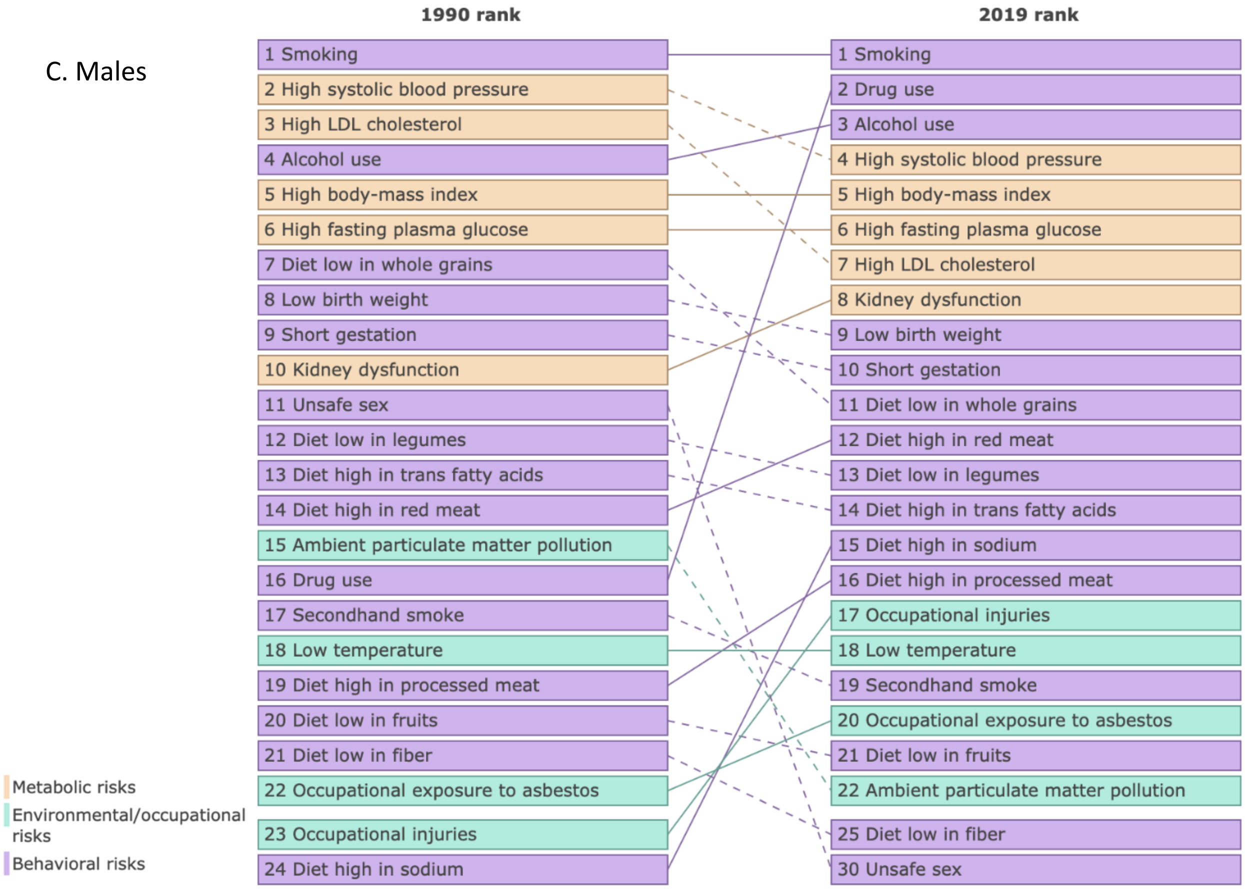
1990 rank
2019 rank
1 Smoking
2 High systolic blood pressure
3 High LDL cholesterol
4 Alcohol use
5 High body-mass index
6 High fasting plasma glucose
7 Diet low in whole grains
8 Low birth weight
9 Short gestation
10 Kidney dysfunction
11 Unsafe sex
12 Diet low in legumes
13 Diet high in trans fatty acids
14 Diet high in red meat
15 Ambient particulate matter pollution
16 Drug use
17 Secondhand smoke
18 Low temperature
19 Diet high in processed meat
20 Diet low in fruits
21 Diet low in fiber
22 Occupational exposure to asbestos
23 Occupational injuries
24 Diet high in sodium
1 Smoking
2 Drug use
3 Alcohol use
4 High systolic blood pressure
5 High body-mass index
6 High fasting plasma glucose
7 High LDL cholesterol
8 Kidney dysfunction
9 Low birth weight
10 Short gestation
11 Diet low in whole grains
12 Diet high in red meat
13 Diet low in legumes
14 Diet high in trans fatty acids
15 Diet high in sodium
16 Diet high in processed meat
17 Occupational injuries
18 Low temperature
19 Secondhand smoke
20 Occupational exposure to asbestos
21 Diet low in fruits
22 Ambient particulate matter pollution
25 Diet low in fiber
30 Unsafe sex
Metabolic risks
Environmental/occupational risks
Behavioral risks

Table S6. Main causes of death and disability-adjusted life-years (DALYs), both sexes and all ages, Colorado, 2019.

| Cause | Deaths | | | DALYs | | |
|---|---|---|---|---|---|---|
| | Central Value | Lower Uncertainty Level | Upper Uncertainty Level | Central Value | Lower Uncertainty Level | Upper Uncertainty Level |
| Acute hepatitis A | 1 | 0 | 1 | 144 | 98 | 206 |
| Acute hepatitis B | 1 | 0 | 1 | 29 | 21 | 40 |
| Acute hepatitis C | 0 | 0 | 1 | 14 | 9 | 22 |
| Acute hepatitis E | 0 | 0 | 0 | 13 | 9 | 19 |
| Acute lymphoid leukemia | 28 | 23 | 35 | 1,076 | 884 | 1,305 |
| Acute myeloid leukemia | 204 | 163 | 250 | 4,517 | 3,660 | 5,479 |
| Alcoholic cardiomyopathy | 64 | 42 | 95 | 1,931 | 1,372 | 2,757 |
| Amphetamine use disorders | 66 | 51 | 83 | 3,927 | 3,086 | 4,847 |
| Anorexia nervosa | 1 | 0 | 1 | 1,318 | 792 | 2,043 |
| Asbestosis | 11 | 7 | 15 | 179 | 131 | 240 |
| Ascariasis | 0 | 0 | 0 | 0 | 0 | 0 |
| Bulimia nervosa | 0 | 0 | 1 | 3,383 | 2,000 | 5,246 |
| Cellulitis | 49 | 22 | 78 | 1,228 | 706 | 1,690 |
| Chlamydial infection | 0 | 0 | 0 | 33 | 22 | 48 |
| Chronic kidney disease | 1262 | 1037 | 1499 | 27,217 | 22,997 | 31,429 |
| Chronic kidney disease due to diabetes mellitus type 1 | 24 | 14 | 40 | 922 | 593 | 1,378 |
| Chronic kidney disease due to diabetes mellitus type 2 | 341 | 244 | 460 | 7,274 | 5,446 | 9,202 |
| Chronic kidney disease due to glomerulonephritis | 128 | 88 | 182 | 3,617 | 2,773 | 4,647 |
| Chronic kidney disease due to hypertension | 545 | 418 | 685 | 8,954 | 7,209 | 10,900 |

| | | | | | | |
|---|---|---|---|---|---|---|
| Chronic kidney disease due to other and unspecified causes | 226 | 154 | 312 | 6,449 | 5,016 | 7,996 |
| Chronic lymphoid leukemia | 89 | 68 | 116 | 1,618 | 1,259 | 2,090 |
| Chronic myeloid leukemia | 24 | 19 | 32 | 540 | 425 | 685 |
| Cirrhosis and other chronic liver diseases due to alcohol use | 311 | 236 | 401 | 8,778 | 6,595 | 11,404 |
| Cirrhosis and other chronic liver diseases due to hepatitis B | 30 | 23 | 39 | 879 | 664 | 1,140 |
| Cirrhosis and other chronic liver diseases due to hepatitis C | 542 | 427 | 686 | 15,854 | 12,355 | 20,105 |
| Cirrhosis and other chronic liver diseases due to NAFLD | 107 | 73 | 153 | 2,830 | 1,856 | 4,118 |
| Cirrhosis and other chronic liver diseases due to other causes | 199 | 142 | 267 | 5,078 | 3,633 | 6,883 |
| Coal workers pneumoconiosis | 4 | 2 | 6 | 67 | 45 | 94 |
| Cocaine use disorders | 121 | 96 | 151 | 9,192 | 7,370 | 11,389 |
| Congenital heart anomalies | 41 | 33 | 50 | 3,041 | 2,501 | 3,712 |
| Congenital musculoskeletal and limb anomalies | 4 | 3 | 5 | 2,089 | 1,469 | 2,850 |
| Cyclist road injuries | 20 | 15 | 25 | 2,085 | 1,669 | 2,577 |
| Diabetes mellitus type 1 | 57 | 43 | 78 | 5,147 | 3,901 | 6,640 |
| Diabetes mellitus type 2 | 841 | 696 | 992 | 47,861 | 37,370 | 60,056 |
| Digestive congenital anomalies | 6 | 4 | 8 | 643 | 460 | 831 |
| Down syndrome | 16 | 12 | 20 | 803 | 646 | 975 |
| Drug-susceptible tuberculosis | 12 | 9 | 14 | 299 | 238 | 357 |
| Ectopic pregnancy | 0 | 0 | 0 | 15 | 10 | 21 |
| Endometriosis | 0 | 0 | 0 | 903 | 550 | 1,414 |
| Extensively drug-resistant tuberculosis | 0 | 0 | 0 | 2 | 0 | 9 |
| Foreign body in other body part | 1 | 1 | 2 | 925 | 668 | 1,238 |

| | | | | | | |
|---|---|---|---|---|---|---|
| G6PD deficiency | 6 | 4 | 7 | 264 | 203 | 332 |
| Gastritis and duodenitis | 11 | 8 | 14 | 885 | 643 | 1,201 |
| Genital prolapse | 0 | 0 | 1 | 318 | 155 | 592 |
| Gonococcal infection | 1 | 1 | 1 | 37 | 29 | 47 |
| Hemolytic disease and other neonatal jaundice | 0 | 0 | 0 | 110 | 84 | 139 |
| HIV/AIDS - Drug-susceptible Tuberculosis | 1 | 1 | 2 | 52 | 32 | 81 |
| HIV/AIDS - Extensively drug-resistant Tuberculosis | 0 | 0 | 0 | 0 | 0 | 2 |
| HIV/AIDS - Multidrug-resistant Tuberculosis without extensive drug resistance | 0 | 0 | 0 | 2 | 0 | 8 |
| HIV/AIDS resulting in other diseases | 57 | 54 | 60 | 3,450 | 2,788 | 4,327 |
| Indirect maternal deaths | 2 | 1 | 2 | 86 | 58 | 120 |
| Intracerebral hemorrhage | 740 | 603 | 883 | 14,389 | 11,895 | 17,210 |
| Ischemic stroke | 1528 | 1193 | 1829 | 27,624 | 23,056 | 32,013 |
| Late maternal deaths | 2 | 1 | 3 | 115 | 65 | 187 |
| Liver cancer due to alcohol use | 119 | 88 | 157 | 2,866 | 2,081 | 3,830 |
| Liver cancer due to hepatitis B | 41 | 31 | 56 | 1,106 | 809 | 1,488 |
| Liver cancer due to hepatitis C | 136 | 107 | 172 | 2,957 | 2,303 | 3,843 |
| Liver cancer due to NASH | 36 | 28 | 47 | 757 | 577 | 984 |
| Liver cancer due to other causes | 29 | 22 | 37 | 758 | 594 | 949 |
| Maternal abortion and miscarriage | 0 | 0 | 0 | 15 | 10 | 21 |
| Maternal deaths aggravated by HIV/AIDS | 0 | 0 | 0 | 0 | 0 | 1 |
| Maternal hemorrhage | 0 | 0 | 1 | 108 | 68 | 168 |
| Maternal hypertensive disorders | 1 | 1 | 1 | 149 | 102 | 211 |

| | | | | | | |
|---|---|---|---|---|---|---|
| Maternal obstructed labor and uterine rupture | 0 | 0 | 0 | 34 | 15 | 59 |
| Maternal sepsis and other maternal infections | 0 | 0 | 0 | 63 | 32 | 112 |
| Motor vehicle road injuries | 429 | 356 | 512 | 24,449 | 20,578 | 28,670 |
| Motorcyclist road injuries | 84 | 66 | 104 | 5,702 | 4,696 | 6,827 |
| Multidrug-resistant tuberculosis without extensive drug resistance | 0 | 0 | 1 | 9 | 1 | 34 |
| Myelodysplastic, myeloproliferative, and other hematopoietic neoplasms | 157 | 113 | 216 | 2,698 | 2,059 | 3,776 |
| Myocarditis | 17 | 10 | 23 | 729 | 469 | 966 |
| Neonatal encephalopathy due to birth asphyxia and trauma | 28 | 23 | 33 | 4,020 | 3,393 | 4,771 |
| Neonatal preterm birth | 75 | 64 | 85 | 14,220 | 12,122 | 16,618 |
| Neonatal sepsis and other neonatal infections | 9 | 7 | 11 | 1,053 | 834 | 1,276 |
| Neural tube defects | 10 | 7 | 12 | 855 | 642 | 1,078 |
| Non-rheumatic calcific aortic valve disease | 325 | 253 | 390 | 3,895 | 3,184 | 4,696 |
| Non-rheumatic degenerative mitral valve disease | 54 | 40 | 79 | 1,279 | 979 | 1,715 |
| Non-rheumatic valvular heart disease | 380 | 297 | 453 | 5,197 | 4,350 | 6,300 |
| Non-venomous animal contact | 3 | 3 | 4 | 616 | 405 | 951 |
| Opioid use disorders | 738 | 608 | 891 | 71,887 | 57,703 | 88,062 |
| Orofacial clefts | 0 | 0 | 0 | 62 | 39 | 90 |
| Other cardiomyopathy | 297 | 228 | 423 | 6,450 | 5,035 | 9,230 |
| Other chromosomal abnormalities | 20 | 16 | 27 | 2,002 | 1,639 | 2,518 |
| Other congenital birth defects | 28 | 23 | 35 | 2,512 | 2,036 | 3,159 |

| | | | | | | |
|---|---|---|---|---|---|---|
| Other drug use disorders | 82 | 64 | 101 | 7,027 | 5,595 | 8,834 |
| Other exposure to mechanical forces | 34 | 27 | 42 | 8,438 | 5,835 | 12,050 |
| Other gynecological diseases | 2 | 1 | 2 | 6,416 | 4,298 | 8,944 |
| Other hemoglobinopathies and hemolytic anemias | 20 | 16 | 25 | 592 | 461 | 769 |
| Other leukemia | 105 | 83 | 132 | 2,014 | 1,591 | 2,504 |
| Other malignant neoplasms | 311 | 257 | 371 | 8,491 | 7,147 | 10,016 |
| Other maternal disorders | 3 | 2 | 5 | 239 | 175 | 313 |
| Other neonatal disorders | 30 | 23 | 38 | 2,917 | 2,290 | 3,646 |
| Other non-rheumatic valve diseases | 1 | 0 | 1 | 23 | 15 | 35 |
| Other pneumoconiosis | 1 | 0 | 2 | 38 | 25 | 51 |
| Other road injuries | 4 | 3 | 5 | 450 | 358 | 560 |
| Other sexually transmitted infections | 0 | 0 | 1 | 130 | 83 | 197 |
| Other urinary diseases | 13 | 8 | 19 | 338 | 244 | 435 |
| Paratyphoid fever | 0 | 0 | 0 | 1 | 0 | 1 |
| Pedestrian road injuries | 100 | 82 | 120 | 5,136 | 4,319 | 6,030 |
| Peptic ulcer disease | 86 | 66 | 107 | 1,749 | 1,404 | 2,158 |
| Physical violence by firearm | 123 | 99 | 148 | 7,392 | 6,047 | 8,896 |
| Physical violence by other means | 39 | 33 | 45 | 3,853 | 3,202 | 4,688 |
| Physical violence by sharp object | 26 | 21 | 31 | 1,690 | 1,398 | 2,005 |
| Poisoning by carbon monoxide | 24 | 20 | 30 | 1,135 | 915 | 1,399 |
| Poisoning by other means | 10 | 8 | 12 | 1,053 | 805 | 1,345 |
| Pulmonary aspiration and foreign body in airway | 149 | 126 | 173 | 4,090 | 3,537 | 4,648 |

| | | | | | | |
|---|---|---|---|---|---|---|
| Pyoderma | 34 | 18 | 63 | 593 | 327 | 1,051 |
| Self-harm by firearm | 579 | 452 | 740 | 23,009 | 18,000 | 29,923 |
| Self-harm by other specified means | 519 | 374 | 636 | 24,207 | 17,701 | 29,322 |
| Sickle cell disorders | 2 | 1 | 3 | 101 | 74 | 137 |
| Silicosis | 4 | 3 | 6 | 103 | 75 | 139 |
| Subarachnoid hemorrhage | 272 | 223 | 327 | 7,747 | 6,486 | 9,115 |
| Syphilis | 1 | 0 | 1 | 65 | 50 | 82 |
| Thalassemias | 1 | 1 | 2 | 91 | 70 | 118 |
| Typhoid fever | 0 | 0 | 0 | 1,000 | 800 | 1,197 |
| Unintentional firearm injuries | 9 | 7 | 12 | 670 | 526 | 863 |
| Urinary tract infections and interstitial nephritis | 287 | 231 | 355 | 4,028 | 3,306 | 4,906 |
| Urogenital congenital anomalies | 4 | 2 | 5 | 422 | 307 | 581 |
| Urolithiasis | 8 | 6 | 11 | 295 | 229 | 371 |
| Uterine fibroids | 0 | 0 | 0 | 821 | 395 | 1,585 |
| Venomous animal contact | 1 | 1 | 1 | 280 | 194 | 381 |
| Visceral leishmaniasis | 0 | 0 | 0 | 0 | 0 | 0 |
| **Communicable, maternal, neonatal, and nutritional diseases** | 1,602 | 1,352 | 1,849 | 67,838 | 59,095 | 77,936 |
| **Injuries** | 3,448 | 2,891 | 4,044 | 183,306 | 155,487 | 215,592 |
| **Non-communicable diseases** | 35,675 | 30,805 | 40,965 | 1,425,347 | 1,206,503 | 1,664,944 |
| **All causes** | **40,724** | **35,134** | **46,743** | **1,676,491** | **1,427,654** | **1,953,173** |

Table S7. GATHER checklist

Checklist of information that should be included in new reports of global health estimates

| Item # | Checklist item | Reported on point # |
|---|---|---|
| Objectives and funding | | |
| 1 | Define the indicator(s), populations (including age, sex, and geographic entities), and time period(s) for which estimates were made. | 2 |
| 2 | List the funding sources for the work. | Financial disclosure |
| Data Inputs | | |
| *For all data inputs from multiple sources that are synthesized as part of the study:* | | |
| 3 | Describe how the data were identified and how the data were accessed. | 2 |
| 4 | Specify the inclusion and exclusion criteria. Identify all ad-hoc exclusions. | 2 |
| 5 | Provide information on all included data sources and their main characteristics. For each data source used, report reference information or contact name/institution, population represented, data collection method, year(s) of data collection, sex and age range, diagnostic criteria or measurement method, and sample size, as relevant. | 2 |
| 6 | Identify and describe any categories of input data that have potentially important biases (e.g., based on characteristics listed in item 5). | 2, 4 |
| *For data inputs that contribute to the analysis but were not synthesized as part of the study:* | | |
| 7 | Describe and give sources for any other data inputs. | - |
| *For all data inputs:* | | |
| 8 | Provide all data inputs in a file format from which data can be efficiently extracted (e.g., a spreadsheet rather than a PDF), including all relevant meta-data listed in item 5. For any data inputs that cannot be shared because of ethical or legal reasons, such as third-party ownership, provide a contact name or the name of the institution that retains the right to the data. | 2, figures 1-3 |
| Data analysis | | |
| 9 | Provide a conceptual overview of the data analysis method. A diagram may be helpful. | 2 |
| 10 | Provide a detailed description of all steps of the analysis, including mathematical formulae. This description should cover, as relevant, data cleaning, data pre-processing, data adjustments and weighting of data sources, and mathematical or statistical model(s). | 2 |
| 11 | Describe how candidate models were evaluated and how the final model(s) were selected. | 2 |
| 12 | Provide the results of an evaluation of model performance, if done, as well as the results of any relevant sensitivity analysis. | 2 |
| 13 | Describe methods for calculating uncertainty of the estimates. State which sources of uncertainty were, and were not, accounted for in the uncertainty analysis. | 4 |
| 14 | State how analytic or statistical source code used to generate estimates can be accessed. | 2, 4 |
| Results and Discussion | | |
| 15 | Provide published estimates in a file format from which data can be efficiently extracted. | 2, figures 1-3 |
| 16 | Report a quantitative measure of the uncertainty of the estimates (e.g. uncertainty intervals). | Table 1 |

| | | |
|---|---|---|
| 17 | Interpret results in light of existing evidence. If updating a previous set of estimates, describe the reasons for changes in estimates. | 4 |
| 18 | Discuss limitations of the estimates. Include a discussion of any modelling assumptions or data limitations that affect interpretation of the estimates. | 4 |

*This checklist should be used in conjunction with the GATHER statement and Explanation and Elaboration document, found on gather-statement.org*

**Table S8.** Key Global Burden of Disease (GBD) papers and sources used as a reference in this analysis

| |
|---|
| Vos T, Lim SS, Abbafati C, et al. Global burden of 369 diseases and injuries in 204 countries and territories, 1990–2019: a systematic analysis for the Global Burden of Disease Study 2019. *Lancet*. 2020;396(10258):1204-1222. doi:10.1016/S0140-6736(20)30925-9 |
| Murray CJL, Aravkin AY, Zheng P, et al. Global burden of 87 risk factors in 204 countries and territories, 1990–2019: a systematic analysis for the Global Burden of Disease Study 2019. *Lancet*. 2020;396(10258):1223-1249. doi:10.1016/S0140-6736(20)30752-2 |
| http://vizhub.healthdata.org/gbd-compare |
